# Cryptic diversity in the subgenus *Oxyphortica* (Diptera, Drosophilidae, *Stegana*)

**DOI:** 10.7717/peerj.12347

**Published:** 2021-10-29

**Authors:** Ya-Lian Wang, Nan-Nan Wang, Yuan Zhang, Shun-Chern Tsaur, Hong-Wei Chen

**Affiliations:** 1 Department of Entomology, South China Agricultural University, Guangzhou, Guangdong, China; 2 Center for General Education, National Taiwan University, Taipei, Taiwan, China

**Keywords:** Cryptic diversity, Phylogeny, Oxyphortica, Drosophilidae, Taxonomy

## Abstract

Phylogenetic relationships of the subgenus *Oxyphortica* were reconstructed based on two mitochondrial genes (*COI* and *ND2*). The results revealed the paraphyly of *Oxyphortica* and supported high levels of cryptic diversity within this subgenus. By integrating morphological characteristics and molecular evidence, we identified 17 new species as members of *Oxyphortica*: *S*. (*O*.) *amphigya* **sp. nov.**, *S*. (*O*.) *armillata* **sp. nov.**, *S*. (*O*.) *ashima* **sp. nov.**, *S*. (*O*.) *bawo* **sp. nov.**, *S*. (*O*.) *crypta* **sp. nov.**, *S*. (*O*.) *gelea* **sp. nov.**, *S*. (*O*.) *hengduanmontana* **sp. nov.**, *S*. (*O*.) *jinmingi* **sp. nov.**, *S*. (*O*.) *mengbalanaxi* **sp. nov.**, *S*. (*O*.) *mouig* **sp. nov.**, *S*. (*O*.) *setipes* **sp. nov.**, *S*. (*O*.) *shangrila* **sp. nov.**, *S*. (*O*.) *tsauri* **sp. nov.**, *S*. (*O*.) *valleculata* **sp. nov.**, *S*. (*O*.) *wanhei* **sp. nov.**, *S*. (*O*.) *yangjin* **sp. nov.** and *S*. (*O*.) *hypophaia* **sp. nov.** To test the early morphological identifications and confirm the species boundaries, different species delimitation methods, including Automatic Barcode Gap Discovery (ABGD) and Bayesian Phylogenetics and Phylogeography (BP&P), were used, together with traditional distance. All species boundaries were clearly defined. As *Oxyphortica* species are mainly distributed across Southwest China (*e.g.*, 20 spp. from the Hengduan Mountains), the complex climate and topographic landforms of the area may be responsible for the high levels of species diversity and endemism.

## Introduction

Within the subfamily Steganinae, the genus *Stegana* Meigen, 1830 is one of the most species-rich genera, comprising 247 species (Bächli, 2021; Wang et al., 2021). These species are classified into five subgenera: *Ceratostylus* Enderlein (1922), *Orthostegana* Hendel (1913), *Oxyphortica* Duda (1923), *Stegana* Meigen (1830) and *Steganina* Wheeler (1960). Among them, the subgenus *Oxyphortica* Duda (1923) is a morphologically conservative taxon that can only be distinguished from the closely related subgenus *Orthostegana* Hendel (1913) by the male genitalia.

The subgenus *Oxyphortica* encompasses 38 species (Bächli, 2021), which mainly live near streams and feed on tree trunk sap, moss, or fungi. These species mostly inhabit the tropical to subtropical broad-leaved forests of the Oriental Region. Nevertheless, the habitats of some species extend beyond these areas—the habitats of *S*. (*O*.) *nigripennis* (Hendel, 1914) and *S*. (*O*.) *dendrobium* Chen & Aotsuka, 2004 extend northward into the southern part of the Palaearctic Region (Kyushu, Japan) and the habitat of *S*. (*O*.) *convergens* (de Meijere, 1911) extends southward into the northern part of the Australian Region (New Guinea) (Brake & Bächli, 2008; Wang et al., 2017; Huang et al., 2018). In this context, Southwest China encompasses a centre of diversity of *Oxyphortica* (55.3% of the known species of this subgenus occur in this area, and 39.5% of them are endemic).

Previous species delineation within *Oxyphortica* has relied heavily on morphological data or *COI* sequences from limited taxa (Wang et al., 2017; Huang et al., 2018). Only two species groups of *Oxyphortica* were recognised based on morphological characteristics: the *convergens* and *nigripennis* species groups, with eight and six species, respectively (Chen & Wang, 2004; Cheng, Xu & Chen, 2010). Li et al. (2013) suggested the paraphyly of *Oxyphortica* based on two mitochondrial (*COI* and *ND2*) markers and one nuclear (*28S*) marker, although they only investigated four representative species (*S*. (*O*.) *aotsukai* Chen & Wang, 2004, *S*. (*O*.) *prigenti* Chen & Wang, 2004, *S*. (*O*.) *adentata* Toda & Peng, 1992 and *S*. (*O*.) *latipenis* Xu, Gao & Chen, 2007). None of the previous studies have attempted to examine the extensive species taxonomic status within *Oxyphortica*.

Accurate species identification is essential for a comprehensive phylogenetic assessment. However, complex evolutionary history often limits the inference of reliable phylogenies and the delimitation of species boundaries, especially in rapidly diversified taxa. Complicating factors include morphologically cryptic species (Low et al., 2016; Machado et al., 2017), horizontal gene transfer (Hurst & Jiggins, 2005), incomplete lineage sorting (Yeates et al., 2011) and introgression caused by multiple hybridisation events (Fougère-Danezan et al., 2015). Nevertheless, compared with traditional taxonomy, the application of new molecular analytical approaches has made unveiling cryptic species diversity easier and faster. In fact, molecular data recently revealed high levels of cryptic diversity of *Oxyphortica* along the Hengduan Mountains and its adjacent regions (Wang et al., 2017; Huang et al., 2018), where the complex montane topography may have promoted and maintained species differentiation.

During recent field trips, a large number of *Oxyphortica* species with similar morphological characteristics were collected from Southwest China. Several of these species vary among different mountain gullies, even if the gullies are close. Considering the limited molecular data analysis and unclear taxonomic status of numerous species, in this study, we aim to assess the boundaries and taxonomic status of 17 new species identified by morphological characteristics and molecular data and briefly discuss the cryptic diversity of *Oxyphortica* in Southwest China.

## Materials & methods

### Sampling and morphological evaluations

Specimens were collected by net sweeping from tussocks or tree trunks along streams in forests. Following collection, the specimens were immediately preserved in 75% ethanol. In the laboratory, all samples collected from the field were initially identified based on morphological characteristics, and then targeted specimens were selected for thorough morphologilical examination. Detailed information is listed in Table 1. Genitalia of the specimens were removed for further identification and photographed using a microscope Mshot Camera (Mshot, China). A small piece of tissue was excised for DNA extraction using a TIANGEN™ DNA Extraction Kit (Tiangen Biotech, Beijing, China). Finally, using a combination of morphological characteristics and molecular data, new species were recognised. Type specimens were air-dried and deposited in the Kunming Institute of Zoology, Chinese Academy of Sciences, Kunming, China (KIZ); the Department of Zoology, Tibet Museum of Natural Science, Lasa, China (TMNS); and the Department of Entomology, South China Agricultural University, Guangzhou, China (SCAU). To unify Drosophilidae terminology and facilitate exchanges between research fields, the male terminalia terminology followed that of Rice et al. (2019).

**Table 1:** List of samples used in this study and GenBank accession numbers.

Groups	Species	Collection locality	*COI*	*ND2*
	*L. angusta* Okada, 1956	Hachioji, Tokyo, Japan	HQ842780*	EU180490*
	*L. quadripunctata* (de Meijere, 1908)	Hachioji, Tokyo, Japan	HQ842781*	EU180491*
	*Pa. brevivena* Chen & Zhang, 2007	Hesong, Menghai, Yunnan	KJ813938*	KJ813972*
	*Pa. punctalata* (Chen & Watabe, 2007)	Mengla, Yunnan	HQ842764*	HQ842785*
	*Ps. meiduo* Zhang & Chen, 2018	Beibeng, Motuo, Xizang	KJ813939*	KJ813973*
	*Ps. meiji* Zhang & Chen, 2018	Muyiji Park, Ximeng, Yunnan	KJ813942*	KJ813975*
	*S*. (*St*.) *acantha* Wu, Gao & Chen, 2010 (♂)	Menglun, Mengla, Yunnan	MH373118	MH373186
	*S*. (*St*.) *ancistrophylla* Wu, Gao & Chen, 2010 (♂)	Wangtianshu, Mengla, Yunnan	MH373119	MH373187
	*S*. (s.s.) *apiciprocera* Cao & Chen, 2010 (♂)	Menglun, Mengla, Yunnan	KF670981*	KP752417*
	*S*. (s.s.) *quadrata* Cao & Chen, 2010 (♂)	Kuankuoshui, Shuiyang, Guizhou	KP179318*	MH373224
	*S*. (*Or*.) *flavicauda* Zhang & Chen, 2012 (♂)	Baihualing, Baoshan, Yunnan	JQ901407*	JQ901408*
	*S*. (*Or*.) *hirsutina* Zhang & Chen, 2012 (♂)	Wuangtianshu, Mengla, Yunnan	HQ842770*	HQ842791*
*Convergens*	*S*. (*O*.) *apicopubescens* Cheng, Xu & Chen, 2010 –1 (♂)	Bapen, Fusui, Guangxi	KY596074*	MH372148
	*S*. (*O*.) *apicopubescens* Cheng, Xu & Chen, 2010 –2 (♂)	Mao′ershan, Xing′an, Guangxi	KY596075*	MH372149
	*S*. (*O*.) *apicopubescens* Cheng, Xu & Chen, 2010 –3 (♂)	Longdong, Guangzhou, Guangdong	KY596076*	MH372150
	*S*. (*O*.) *apicopubescens* Cheng, Xu & Chen, 2010 –4 (♂)	Mulun, Huanjiang, Guangxi	KY596077*	MH372151
	*S*. (*O*.) *apicosetosa* Cheng, Xu & Chen, 2010 –1 (♂)	Jiuwanshan, Rongshui, Guangxi	KY596078*	MH372152
	*S*. (*O*.) *apicosetosa* Cheng, Xu & Chen, 2010 –2 (♂)	Nanling, Shaoguan, Guangdong	KY596079*	MH372153
	*S*. (*O*.) *apicosetosa* Cheng, Xu & Chen, 2010 –3 (♂)	Mao′ershan, Xing′an, Guangxi	KY596080*	MH372154
	*S*. (*O*.) *apicosetosa* Cheng, Xu & Chen, 2010 –4 (♀)	Mao′ershan, Xing′an, Guangxi	KY596081*	MH372155
	*S*. (*O*.) *apicosetosa* Cheng, Xu & Chen, 2010 –5 (♂)	Sanchahe, Xishui, Guizhou	KY596082*	MH372156
	*S*. (*O*.) *convergens* (de Meijere, 1911) –1 (♂)	Bahsienshan, Taichung, Taiwan	KF642615*	MH372157
	*S*. (*O*.) *convergens* (de Meijere, 1911) –2 (♂)	Wulai, Hsinpei, Taiwan	MH372086	MH372158
	*S*. (*O*.) *convergens* (de Meijere, 1911) –3 (♀)	Mudan, Pingtung, Taiwan	KY596083*	MH372159
	*S*. (*O*.) *convergens* (de Meijere, 1911) –4 (♂)	SunMoon Lake, Nantou, Taiwan	KY596084*	MH372160
	*S*. (*O*.) *gonglui* Wang & Chen, 2018 –1 (♂)	Husa, Longchuan, Yunnan	KY596100*	MH372161
	*S*. (*O*.) *gonglui* Wang & Chen, 2018 –2 (♂)	Husa, Longchuan, Yunnan	KY596101*	MH372162
	*S*. (*O*.) *gonglui* Wang & Chen, 2018 –3 (♂)	Nabang, Yingjiang, Yunnan	KY596102*	MH372163
	*S*. (*O*.) *mediospinosa* Cheng, Xu & Chen, 2010 –1 (♂)	Wangtianshu, Mengla, Yunnan	KY596085*	MH372164
	*S*. (*O*.) *mediospinosa* Cheng, Xu & Chen, 2010 –2 (♂)	Gnomnolat, Khammouane, Laos	KY596086*	MH372165
	*S*. (*O*.) *mediospinosa* Cheng, Xu & Chen, 2010 –3 (♀)	SunMoon Lake, Nantou, Taiwan	KY596087*	MH372166
	*S*. (*O*.) *mediospinosa* Cheng, Xu & Chen, 2010 –4 (♂)	Wangtianshu, Mengla, Yunnan	KY596088*	MH372167
	*S*. (*O*.) *mediospinosa* Cheng, Xu & Chen, 2010 –5 (♂)	Yixiang, Pu′er, Yunnan	KY596089*	MH372168
	*S*. (*O*.) *mediospinosa* Cheng, Xu & Chen, 2010 –6 (♂)	Yixiang, Pu′er, Yunnan	KY596090*	MH372169
	*S*. (*O*.) *mediospinosa* Cheng, Xu & Chen, 2010 –7 (♂)	Menglun, Mengla, Yunnan	KY596091*	MH372170
	*S*. (*O*.) *mediospinosa* Cheng, Xu & Chen, 2010 –8 (♂)	Guanlei, Mengla, Yunnan	KY596092*	MH372171
	*S*. (*O*.) *mediospinosa* Cheng, Xu & Chen, 2010 –9 (♂)	Longtan Park, Ximeng, Yunnan	KY596093*	MH372172
	*S*. (*O*.) *mediospinosa* Cheng, Xu & Chen, 2010 –10 (♂)	Guanlei, Mengla, Yunnan	KY596094*	MH372173
	*S*. (*O*.) *mediospinosa* Cheng, Xu & Chen, 2010 –11 (♂)	Likan, Ximeng, Yunnan	KY596095*	MH372174
	*S*. (*O*.) *setifrons* Sidorenko, 1997 –1 (♂)	Wuyishan, Fujian and Jiangxi	KY596096*	MH372175
	*S*. (*O*.) *setifrons* Sidorenko, 1997 –2 (♂)	Haoping, Ziyang, Shaanxi	KY596097*	MH372176
	*S*. (*O*.) *setifrons* Sidorenko, 1997 –3 (♀)	Tianmushan, Linan, Zhejiang	KY596099*	MH372177
	*S*. (*O*.) *setifrons* Sidorenko, 1997 –4 (♂)	Haoping, Ziyang, Shaanxi	KY596098*	MH372178
	*S*. (*O*.) *xiaoyangae* Zhang & Chen, 2018 –1 (♂)	Qizimeishan, Xuan′en, Hubei	KY596103*	MH372179
	*S*. (*O*.) *xiaoyangae* Zhang & Chen, 2018 –2 (♂)	Qizimeishan, Xuan′en, Hubei	KY596104*	MH372180
	*S*. (*O*.) *zhulinae* Wang & Chen, 2018 –1 (♂)	Nabang, Yingjiang, Yunnan	KY596105*	MH372181
	*S*. (*O*.) *zhulinae* Wang & Chen, 2018 –2 (♂)	Husa, Longchuan, Yunnan	KY596106*	MH372182
	*S*. (*O*.) *zhulinae* Wang & Chen, 2018 –3 (♂)	Husa, Longchuan, Yunnan	KY596107*	MH372183
	*S*. (*O*.) *zhulinae* Wang & Chen, 2018 –4 (♂)	Husa, Longchuan, Yunnan	KY596108*	MH372184
*Nigripennis*	*S*. (*O*.) *aotsukai* Chen & Wang, 2004 –1 (♂)	Maoershan, Xing′an, Guangxi	HQ842776*	HQ842797*
	*S*. (*O*.) *aotsukai* Chen & Wang, 2004 –2 (♂)	Jianfengling, Ledong, Hainan	KY596071*	MH372103
	*S*. (*O*.) *aotsukai* Chen & Wang, 2004 –3 (♂)	Mao′ershan, Xing′an, Guangxi	KY596072*	MH372104
	*S*. (*O*.) *aotsukai* Chen & Wang, 2004 –4 (♂)	Maolan, Libo, Guizhou	KY596073*	MH372105
	*S*. (*O*.) *nigripennis* (Hendel, 1914) –1 (♂)	Wulai, Hsinpei, Taiwan	KF642623*	MH372106
	*S*. (*O*.) *nigripennis* (Hendel, 1914) –2 (♂)	Guanghua, Chiayi, Taiwan	KF642624*	MH372107
	*S*. (*O*.) *prigenti* Chen & Wang, 2004 –1 (♂)	Wangtianshu, Mengla, Yunnan	HQ842775*	MH372108
	*S*. (*O*.) *prigenti* Chen & Wang, 2004 –2 (♂)	Wangtianshu, Mengla, Yunnan	MH372081	MH372109
Ungrouped species	*S*. (*O*.) *acutipenis* Xu, Gao & Chen, 2007 –1 (♂)	Wangtianshu, Mengla, Yunnan	KY829362*	MH372110
	*S*. (*O*.) *acutipenis* Xu, Gao & Chen, 2007 –2 (♂)	Menglun, Mengla, Yunnan	KY829364*	MH372111
	*S*. (*O*.) *acutipenis* Xu, Gao & Chen, 2007 –3 (♂)	Menglun, Mengla, Yunnan	KY829363*	MH372112
	*S*. (*O*.) *adentata* Toda & Peng, 1992 –1 (♂)	Guanlei, Mengla, Yunnan	KY829370*	MH372113
	*S*. (*O*.) *adentata* Toda & Peng, 1992 –2 (♂)	Yixiang, Pu′er, Yunnan	KY829366*	MH372114
	*S*. (*O*.) *adentata* Toda & Peng, 1992 –3 (♂)	Yixiang, Pu′er, Yunnan	KY829367*	MH372115
	*S*. (*O*.) *adentata* Toda & Peng, 1992 –4 (♂)	Nanling, Shaoguan, Guangdong	HQ842774*	HQ842795*
	*S*. (*O*.) *adentata* Toda & Peng, 1992 –5 (♂)	Conghua, Guangzhou, Guangdong	KY829368*	MH372116
	*S*. (*O*.) *adentata* Toda & Peng, 1992 –6 (♂)	Diaoluoshan, Lingshui, Hainan	KY829369*	MH372117
	*S*. (*O*.) *adentata* Toda & Peng, 1992 –7 (♂)	Mulun, Huanjiang, Yunnan	KY829371*	MH372118
	*S*. (*O*.) *adentata* Toda & Peng, 1992 –8 (♂)	Yixiang, Pu′er, Yunnan	MH372082	MH372119
	*S*. (*O*.) *chuanjiangi* Zhang & Chen, 2017 –1 (♀)	Wuliangshan, Mengla, Yunnan	KY829430*	MH372120
	*S*. (*O*.) *chuanjiangi* Zhang & Chen, 2017 –2 (♀)	Wantianshu, Mengla, Yunnan	KY829429*	MH372121
	*S*. (*O*.) *chuanjiangi* Zhang & Chen, 2017 –3 (♀)	Hesong, Menghai, Yunnan	KY829428*	MH372122
	*S*. (*O*.) *chuanjiangi* Zhang & Chen, 2017 –4 (♂)	Hesong, Menghai, Yunnan	MH372083	MH372123
	*S*. (*O*.) *chuanjiangi* Zhang & Chen, 2017 –5 (♂)	Hesong, Menghai, Yunnan	KY829432*	MH372124
	*S*. (*O*.) *chuanjiangi* Zhang & Chen, 2017 –6 (♂)	Baihualing, Baoshan, Yunnan	KY829433*	MH372125
	*S*. (*O*.) *chuanjiangi* Zhang & Chen, 2017 –7 (♂)	Baihualing, Baoshan, Yunnan	KY829434*	MH372126
	*S*. (*O*.) *curvata* Wang, Gao & Chen, 2010 –1 (♂)	Mengdong, Guanyuan, Yunnan	MH235746	MH372240
	*S*. (*O*.) *curvata* Wang, Gao & Chen, 2010 –2 (♂)	Qimaba, Lüchun, Yunnan	MH235747	MH372241
	*S*. (*O*.) *curvata* Wang, Gao & Chen, 2010 –3 (♂)	Husa, Longchuan, Yunnan	MH235745	MH372242
	*S*. (*O*.) *dainuo* Wang & Chen, 2017 –1 (♂)	Mengdong, Cangyuan, Yunnan	KY829425*	MH372127
	*S*. (*O*.) *dainuo* Wang & Chen, 2017 –2 (♂)	Husa, Longchuan, Yunnan	KY829431*	MH372128
	*S*. (*O*.) *dawa* Zhang & Chen, 2017 (♂)	Beibeng, Motuo, Xizang	KY829437*	MH372129
	*S*. (*O*.) *hirtipenis* Xu, Gao & Chen, 2007 –1 (♂)	Wangtianshu, Mengla, Yunnan	KY829390*	MH372130
	*S*. (*O*.) *hirtipenis* Xu, Gao & Chen, 2007 –2 (♂)	Menglun, Mengla, Yunnan	KY829395*	MH372131
	*S*. (*O*.) *hirtipenis* Xu, Gao & Chen, 2007 –3 (♂)	Guanlei, Mengla, Yunnan	KY829394*	MH372132
	*S*. (*O*.) *laohlie* Zhang & Chen, 2017 –1 (♂)	Muyiji Park, Ximeng, Yunnan	KY829417*	MH372133
	*S*. (*O*.) *laohlie* Zhang & Chen, 2017 –2 (♂)	Muyiji Park, Ximeng, Yunnan	KY829418*	MH372134
	*S*. (*O*.) *laohlie* Zhang & Chen, 2017 –3 (♂)	Muyiji Park, Ximeng, Yunnan	KY829419*	MH372135
	*S*. (*O*.) *laohlie* Zhang & Chen, 2017 –4 (♂)	Muyiji Park, Ximeng, Yunnan	KY829422*	MH372136
	*S*. (*O*.) *latipenis* Xu et al., 2007 –1 (♂)	Wangtianshu, Mengla, Yunnan	HQ842773*	HQ842794*
	*S*. (*O*.) *latipenis* Xu et al., 2007 –2 (♂)	Wangtianshu, Mengla, Yunnan	KY829396*	MH372137
	*S*. (*O*.) *luchun* Wang & Chen, 2017 –1 (♂)	Xiaoheijiang, Lüchuan, Yunnan	KY829423*	MH372138
	*S*. (*O*.) *luchun* Wang & Chen, 2017 –2 (♂)	Xiaoheijiang, Lüchuan, Yunnan	KY829424*	MH372139
	*S*. (*O*.) *maichouensis* Sidorenko, 1998 –1 (♂)	Menglun, Mengla, Yunnan	KY829399*	MH372140
	*S*. (*O*.) *maichouensis* Sidorenko, 1998 –2 (♂)	Menglun, Mengla, Yunnan	MH372084	MH372141
	*S*. (*O*.) *maichouensis* Sidorenko, 1998 –3 (♂)	Menglun, Mengla, Yunnan	MH372085	MH372142
	*S*. (*O*.) *maichouensis* Sidorenko, 1998 –4 (♂)	Mengyuan, Mengla, Yunnan	KY829398*	MH372143
	*S*. (*O*.) *mengwan* Wang & Chen, 2017 –1 (♂)	Husa, Longchuan, Yunnan	KY829439*	MH372144
	*S*. (*O*.) *mengwan* Wang & Chen, 2017 –2 (♂)	Botanic Garden, Ruili, Yunnan	KY829438*	–
	*S*. (*O*.) *triodonta* Cheng & Chen, 2017 –1 (♂)	Muyiji Park, Ximeng, Yunnan	KY829416*	MH372145
	*S*. (*O*.) *triodonta* Cheng & Chen, 2017 –2 (♂)	Menglun, Mengla, Yunnan	KY829427*	MH372146
	*S*. (*O*.) *triodonta* Cheng & Chen, 2017 –3 (♂)	Huanglianshan, Lüchun, Yunnan	KY829426*	MH372147
	*S*. (*O*.) *wanglei* Wang, Gao & Chen, 2010 –1 (♂)	Wuliangshan, Jingdong, Yunnan	MH235778	MH372195
	*S*. (*O*.) *wanglei* Wang, Gao & Chen, 2010 –2 (♂)	Niuluohe, Jiangcheng, Yunnan	MH235779	MH372196
	*S*. (*O*.) *wanglei* Wang, Gao & Chen, 2010 –3 (♂)	Zhengxing, Jinggu, Yunnan	MH235777	MH372197
	*S*. (*O*.) *wanglei* Wang, Gao & Chen, 2010 –4 (♂)	Kangping, Jiangcheng, Yunnan	MH235781	MH372198
	*S*. (*O*.) *wanglei* Wang, Gao & Chen, 2010 –5 (♂)	Tongbiguan, Yingjiang, Yunnan	MH235780	MH372199
	*S*. (*O*.) *wuliangi* Wang, Gao & Chen, 2010 (♂)	Niuluohe, Jiangcheng, Yunnan	MH235789	MH372200
	*S*. (*O*.) *amphigya* sp. nov. –1 (♂)	Mengdong, Guanyuan, Yunnan	MH235736	MH372236
	*S*. (*O*.) *amphigya* sp. nov. –2 (♂)	Mengdong, Guanyuan, Yunnan	MH235737	MH372237
	*S*. (*O*.) *amphigya* sp. nov. –3 (♂)	Husa, Longchuan, Yunnan	MH235738	MH372238
	*S*. (*O*.) *amphigya* sp. nov. –4 (♂)	Baihualing, Baoshan, Yunnan	MH235739	MH372239
	*S*. (*O*.) *armillata* sp. nov. –1 (♂)	Dashuigou, Lüchun, Yunnan	MH235740	MH372244
	*S*. (*O*.) *armillata* sp. nov. –2 (♂)	Sanmeng, Lüchun, Yunnan	MH235741	MH372245
	*S*. (*O*.) *ashima* sp. nov. (♂)	Cangshan, Yangbi, Yunnan	MH235742	MH372201
	*S*. (*O*.) *bawo* sp. nov. –1 (♂)	Hutiaoxia, Shanglira, Yunnan	MH235744	MH372202
	*S*. (*O*.) *bawo* sp. nov. –2 (♀)	Zhengxing, Jinggu, Yunnan	MH235743	MH372203
	*S*. (*O*.) *crypta* sp. nov. (♂)	Dashuigou, Lüchun, Yunnan	MH235749	MH372246
	*S*. (*O.) gelea* sp. nov. –1 (♂)	Tongmai, Bomi, Xizang	MH235750	MH372204
	*S*. (*O*.) *gelea* sp. nov. –2 (♀)	Beibeng, Motuo, Xizang	MH235751	MH372205
	*S*. (*O*.) *gelea* sp. nov. –3 (♀)	Tongmai, Bomi, Xizang	MH235752	MH372206
	*S*. (*O*.) *hengduanmontana* sp. nov. –1 (♂)	Yixiang, Pu′er, Yunnan	MH235753	MH372207
	*S*. (*O*.) *hengduanmontana* sp. nov. –2 (♂)	Yixiang, Pu′er, Yunnan	MH235754	MH372208
	*S*. (*O*.) *hypophaia* sp. nov. (♂)	Xinling, Badong, Hubei	MH235791	MH372235
	*S*. (*O*.) *jinmingi* sp. nov. –1 (♂)	Yixiang, Pu′er, Yunnan	MH235760	MH372209
	*S*. (*O*.) *jinmingi* sp. nov. –2 (♂)	Yixiang, Pu′er, Yunnan	MH235759	MH372210
	*S*. (*O*.) *jinmingi* sp. nov. –3 (♂)	Hesong, Menghai, Yunnan	MH235755	MH372211
	*S*. (*O*.) *mengbalanaxi* sp. nov. –1 (♂)	Baihualing, Baoshan, Yunnan	MH235762	MH372212
	*S*. (*O*.) *mengbalanaxi* sp. nov. –2 (♂)	Huanglianshan, Lüchun, Yunnan	MH235761	MH372213
	*S*. (*O*.) *mouig* sp. nov. –1 (♂)	Yixiang, Pu′er, Yunnan	MH235765	MH372214
	*S*. (*O*.) *mouig* sp. nov. –2 (♂)	Yixiang, Pu′er, Yunnan	MH235764	MH372215
	*S*. (*O*.) *mouig* sp. nov. –3 (♂)	Wuliangshan, Jingdong, Yunnan	MH235763	MH372216
	*S*. (*O*.) *mouig* sp. nov. –4 (♂)	Huangcaoling, Jingdong, Yunnan	MH235769	MH372217
	*S*. (*O*.) *mouig* sp. nov. –5 (♂)	Mengdong, Guanyuan, Yunnan	MH235768	MH372218
	*S*. (*O*.) *mouig* sp. nov. –6 (♂)	Mengdong, Guanyuan, Yunnan	MH235767	MH372219
	*S*. (*O*.) *mouig* sp. nov. –7 (♂)	Huanglianshan, Lüchun, Yunnan	MH235770	MH372220
	*S*. (*O*.) *mouig* sp. nov. –8 (♂)	Husa, Longchuan, Yunnan	MH235766	MH372221
	*S*. (*O*.) *setipes* sp. nov. (♂)	Hesong, Menghai, Yunnan	MH235771	MH372243
	*S*. (*O*.) *shangrila* sp. nov. (♂)	Hutiaoxia, Shangrila, Yunnan	MH235772	MH372222
	*S*. (*O*.) *tsauri* sp. nov. (♂)	Dabang, Chiayi, Taiwan	MH235773	MH372223
	*S*. (*O*.) *valleculata* sp. nov. –1 (♂)	Muyiji Park, Ximeng, Yunnan	MH235776	MH372231
	*S*. (*O*.) *valleculata* sp. nov. –2 (♂)	Yixiang, Pu′er, Yunnan	MH235775	MH372232
	*S*. (*O*.) *valleculata* sp. nov. –3 (♂)	Mengdong, Guanyuan, Yunnan	MH235774	MH372233
	*S*. (*O*.) *wanhei* sp. nov. –1 (♂)	Hutiaoxia, Shanglira, Yunnan	MH235782	MH372224
	*S. (O.) wanhei* sp. nov. –2 (♀)	Menglun, Mengla, Yunnan	MH235783	MH372225
	*S. (O.) wanhei* sp. nov. –3 (♀)	Niuluohe, Jiangcheng, Yunnan	MH235784	MH372226
	*S*. (*O*.) *wanhei* sp. nov. –4 (♂)	Xianheping, Xingyi, Guizhou	MH235788	MH372227
	*S. (O.) wanhei* sp. nov. –5 (♀)	Huangcaoling, Jingdong, Yunnan	MH235787	MH372228
	*S*. (*O*.) *wanhei* sp. nov. –6 (♂)	Nanling, Lancang, Yunnan	MH235785	MH372229
	*S*. (*O*.) *wanhei* sp. nov. –7 (♂)	Nanling, Lancang, Yunnan	MH235786	MH372230
	*S*. (*O*.) *yangjin* sp. nov. (♂)	Beibeng, Motuo, Xizang	MH235790	MH372234

**Note:**

The asterisk sequences obtained from Li et al. (2010), Li et al. (2013), Zhang et al. (2012), Zhang, Tsaur & Chen (2014), Wang et al. (2017), Huang et al. (2018). Abbreviations: *L.*, genus *Leucophenga*; *Pa.*, genus *Parastegana*; *Ps.*, genus *Pseudostegana*; *S*., genus *Stegana*; *St*., subgenus *Steganina*; s.s., *sensu* stricto; *Or*., subgenus *Orthostegana*; *O*., subgenus *Oxyphortica*. All specimens were collected from China except *S*. (*O*.) *mediospinosa* Cheng, Xu & Chen, 2010 –2, which was collected from Laos.

The electronic version of this article in portable document format will represent a published work according to the International Commission on Zoological Nomenclature (ICZN), and hence the new names contained in the electronic version are effectively published under that Code from the electronic edition alone. This published work and the nomenclatural acts it contains have been registered in ZooBank, the online registration system for the ICZN. The ZooBank LSIDs (Life Science Identifiers) can be resolved and the associated information viewed through any standard web browser by appending the LSID to the prefix http://zoobank.org/. The LSID for this publication is: urn:lsid:zoobank.org:pub:1D19FB6F-DBF2-4982-9C9A-D8FBFC1F3F1E. The online version of this work is archived and available from the following digital repositories: PeerJ, PubMed Central and CLOCKSS.

### PCR amplification and sequencing

Two mitochondrial (cytochrome *c* oxidase subunit I (*COI*) and NADH dehydrogenase subunit 2 (*ND2*)) markers were employed for PCR amplification, which involved the following steps: initial pre-denaturation for 3 min at 94 °C, followed by 35 cycles of 30 s at 94 °C, 1 min at 49–56 °C and 1 min at 72 °C, and subsequent post-extension for 5 min at 72 °C. The amplification and sequencing primers are listed in Table 2. Sequencing of the PCR products was performed using an ABI 3700 sequencer; a standard sequencing reaction using a BigDye™ Terminator Kit (Perkin-Elmer, Waltham, MA, USA) was performed with 25 cycles of denaturation for 10 s at 96 °C, annealing for 5 s at 50 °C and extension for 4 min at 60 °C, subsequently followed by purification with 75% isopropyl alcohol. The sequence accession numbers are listed in Table 1.

**Table 2:** Primers used for PCR and sequencing in this study.

Primer name	Primer sequence (5′-3′)	References
*COI*-F1	CGCCTAAACTTCAGCCACTT	He et al. (2009)
LCO1490	GGTCAACAAATCATAAAGATATTGG	Folmer et al. (1994)
HCO2198	TAAACTTCAGGGTGACCAAAAAATCA	
*ND2*-H	AAGCTACTGGGTTCATACC	Park (1999)
*ND2*-T3	AGGCGATAGATTGTAAATC	He et al. (2009)

### Phylogenetic analyses

The mitochondrial sequences were aligned separately for each gene using the programme MAFFT v. 7 (http://mafft.cbrc.jp/alignment/server/) and then concatenated (*COI* + *ND2*). A total of six data blocks of the concatenated data (*i.e.*, first-, second- and third-codon positions of two mitochondrial genes) were subjected to an evaluation of the best partitioning scheme and substitution models (GTR+I+G: *COI*^1st^ + *COI*^2nd^ + *ND2*^1st^ + *ND2*^2nd^; TIM+I+G: *COI*^3rd^ + *ND2*^3rd^) for phylogenetic analysis in PartitionFinder 2 (Lanfear et al., 2017) using the ′greedy′ algorithm and the Bayesian information criterion. Phylogenetic analyses were then performed using Bayesian inference and maximum likelihood (ML) methods. Twelve outgroup species (*Leucophenga angusta* Okada, 1956; *L. quadripunctata* (de Meijere, 1908); *Parastegana brevivena* Chen & Zhang, 2007; *Pa. punctalata* (Chen & Watabe, 2007); *Pseudostegana meiduo* Zhang & Chen, 2018; *Ps. meiji* Zhang & Chen, 2018; *S*. (*Steganina*) *acantha* Wu, Gao & Chen, 2010; *S*. (*St*.) *ancistrophylla* Wu, Gao & Chen, 2010; *S*. (*sensu* stricto) *apiciprocera* Cao & Chen, 2010; *S*. (s.s.) *quadrata* Cao & Chen, 2010; *S*. (*Orthostegana*) *flavicauda* Zhang & Chen, 2012 and *S*. (*Or*.) *hirsutina* Zhang & Chen, 2012) were selected from three Steganinae genera (*Leucophenga*, *Parastegana* and *Pseudostegana*) and three closely related subgenera (*Stegana*, *Steganina* and *Orthostegana*), respectively. Several sequences from the Steganinae genera and subgenera as well as from *Stegana* genus *sensu* stricto were downloaded from GenBank.

Bayesian inference was performed using MrBayes v.3.2.1 (Huelsenbeck & Ronquist, 2001; Ronquist & Huelsenbeck, 2003) on the CIPRES Science Gateway (http://www.phylo.org; Miller, Pfeiffer & Schwartz, 2010). Two independent runs were implemented in parallel, each with 20,000,000 generations. A sampling frequency of every 1,000 generations was employed, and 5,000 early-phase samples were discarded as burn-in. When the average standard deviation of split frequencies was <0.01, the analysis was considered convergent. The ML analysis was performed using IQ-Tree (Nguyen et al., 2015) under the aforementioned substitution models. Node support was assessed *via* ultrafast bootstrap (UFBP) analysis with 1,000 replicates. MEGA 5 was used to estimate pairwise genetic distances (*p*-distance) (Tamura et al., 2011).

### Species delimitation

Species delineation was explored using Automatic Barcode Gap Discovery (ABGD; Puillandre et al., 2012) for each of the two single-locus data, and using Bayesian Phylogenetics and Phylogeography (BP&P; Rannala & Yang, 2003; Yang & Rannala, 2010) for the concatenated dataset. The ABGD analysis was performed at the web interface (http://www.abi.snv.jussieu.fr/public/abgd/) with parameters that included *P* from 0.001 to 0.1, relative gap width (X = 1.0) and the Kimura-2-parameter (K2P; Kimura, 1980) model. The BP&P analysis was implemented in the BP&P v.3.2 programme with the method A11 (Yang, 2015). In the Bayesian analysis, the ancestral population size (θ), root age (τ0) and key priors of the Bayesian species delimitation may strongly affect the posterior probabilities of the speciation modes (Yang & Rannala, 2010); therefore, four different gamma prior (G) combinations were considered for the BP&P coalescent analysis: (1) large ancestral population sizes and deep divergences, θ~G (1, 10) and τ0~G (1, 10); (2) small ancestral population sizes and shallow divergences, θ~G (2, 2,000) and τ0~G (2, 2,000); (3) large ancestral population sizes and shallow divergences, θ~G (1, 10) and τ0~G (2, 2,000); (4) small ancestral population sizes and deep divergences, θ~G (2, 2,000) and τ0~G (1, 10). All analyses were performed twice to confirm consistency. The starting guide tree was first generated using BEAST v.1.10.4 (Suchard et al., 2018) with the GTR + G + I substitution model and the Yule speciation model. We set the length of the chain to 20,000,000, sampled every 2,000 generations and discarded the first 25% as burn-in. The convergence was evaluated using Tracer v.1.7 (Rambaut et al., 2018). When the effective sample size was >200, the analysis was considered convergent.

## Results

Both phylogenetic analyses and the species delimitations were performed using 138 *COI* and 137 *ND2* sequences. Within this dataset, 620 nucleotide sites of *COI* sequences contained 366 conserved, 254 variable and 234 parsimony-informative sites, whereas 1,034 nucleotide sites of *ND2* sequences contained 411 conserved, 620 variable and 577 parsimony-informative sites. All new sequences characterised in this study were uploaded to GenBank. The accession numbers are listed in Table 1.

**Figure 1:**
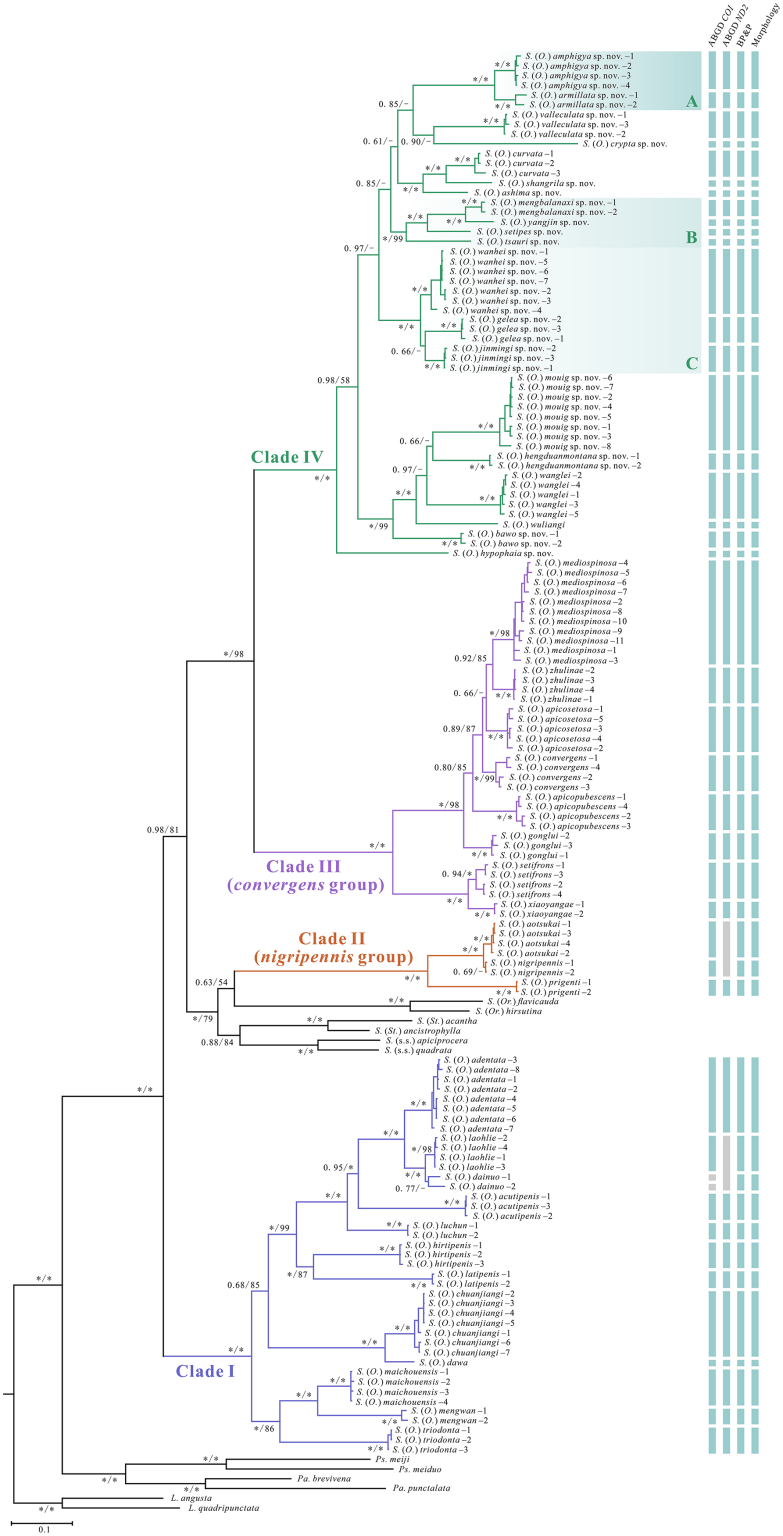
Phylogenetic tree constructed from the Bayesian analysis based on the concatenated dataset. Numbers around the nodes indicate the Bayesian posterior probability (PP) values and ultrafast bootstrap (UFBP) values from maximum likelihood analysis. An asterisk (*) indicates PP = 1.00/UFBP = 100. Abbreviations: *L.*, genus *Leucophenga*; *Pa.*, genus *Parastegana*; *Ps.*, genus *Pseudostegana*; *S*., genus *Stegana*; s.s., *sensu* stricto; *St*., subgenus *Steganina*; *O*., subgenus *Oxyphortica*; *Or*., subgenus *Orthostegana*.

The topology of the phylogenetic tree varied among the different datasets and methods (Figs 1, S1–S5). The concatenated data (*COI* + *ND2*) analyses supported the reciprocal monophyly of all species, whereas three subgenera (*Stegana*, *Steganina* and *Orthostegana*) was found to be nested in the subgenus *Oxyphortica*, rendering the latter paraphyletic. The *Oxyphortica* is mainly composed of four clades (Fig. 1). Clade I comprises 12 known species located at the base of the phylogenetic trees (PP = 1.00; UFBP = 100). Clade II [represented by *S*. (*O*.) *aotsukai*, *S*. (*O*.) *nigripennis* and *S*. (*O*.) *prigenti* of the *nigripennis* group] forms a sister group of the subgenus *Orthostegana* (represented by *S*. (*Or*.) *flavicauda* Zhang & Chen, 2012 and *S*. (*Or*.) *hirsutina* Zhang & Chen, 2012) (PP = 0.63; UFBP = 54). Clade IV comprises all new species and three known species (*S*. (*O*.) *curvata* Wang, Gao & Chen, 2010, *S*. (*O*.) *wuliangi* Wang, Gao & Chen, 2010 and *S*. (*O*.) *wanglei* Wang, Gao & Chen, 2010) and is a sister group to Clade III of the *convergens* group with strong support (PP = 1.00; UFBP = 98). Within Clade IV, species of Clades IV-A, -B and -C share highly similar male genitalia structures. All new species were supported by the morphological characteristics and molecular evidence.

The species delimitation analyses suggested that the boundaries of all sampled species were well-resolved. The number of recognised molecular operational taxonomic units (MOTUs) ranged from 41 to 44 across the datasets and methods used in the present study (Fig. 1). For ABGD, 44 and 41 MOTUs were defined by the *COI* and *ND2* alignments, respectively. *S*. (*O*.) *dainuo* Wang & Chen, 2017 was split into two MOTUs according to *COI*, whereas *S*. (*O*.) *dainuo* and *S*. (*O*.) *laohlie* Zhang & Chen, 2017 formed one MOTU and *S*. (*O*.) *aotsukai* and *S*. (*O*.) *nigripennis* formed another MOTU according to *ND2*. For BP&P, 43 MOTUs were defined by the concatenated *COI* and *ND2* alignments (Table S1), consistent with the species definition based on morphological characteristics.

**Figure 2:**
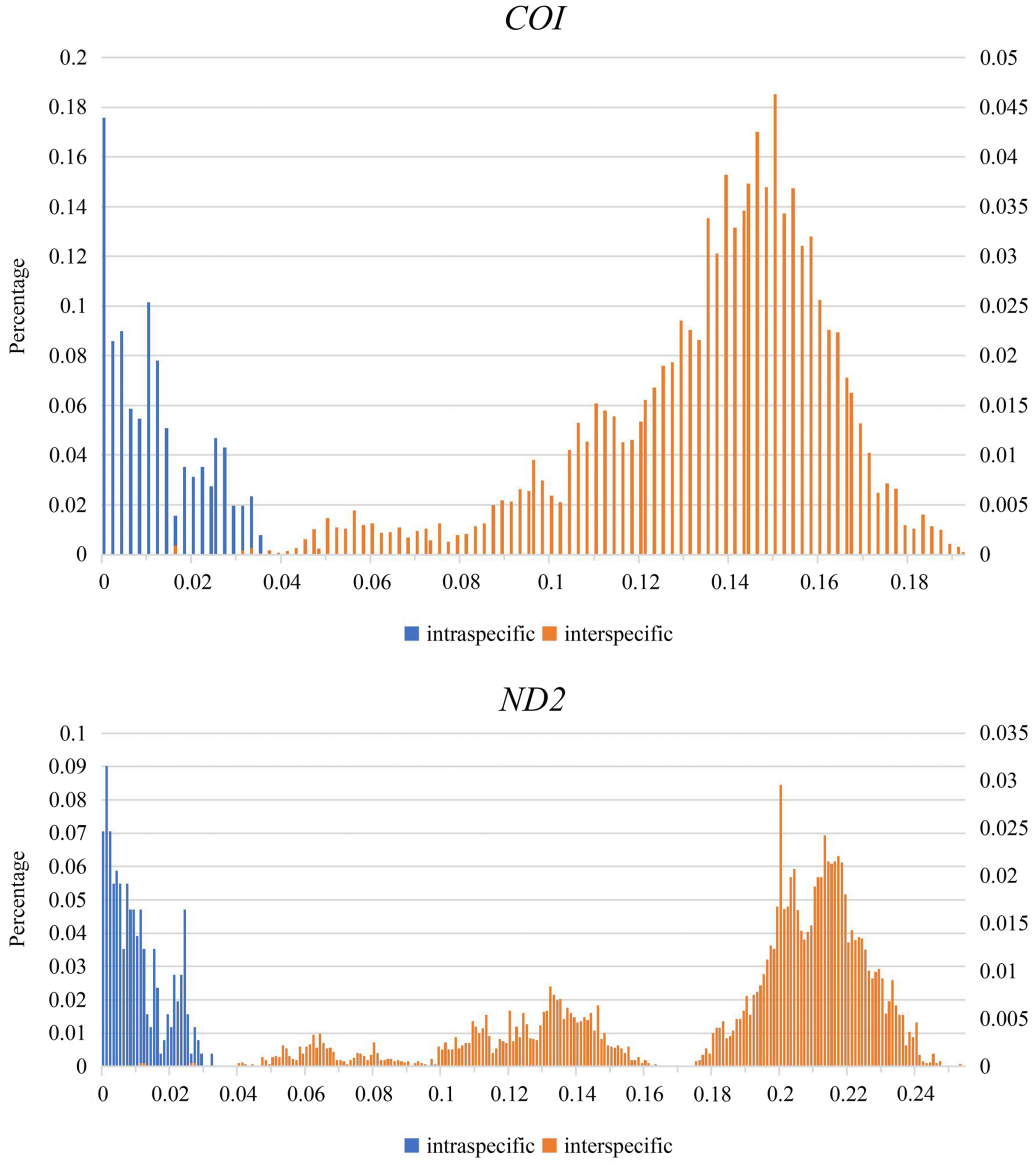
Distribution of intra- and interspecific pairwise genetic *p*-distances.

Partial overlaps occurred between the intra- and interspecific genetic divergences for *COI* and *ND2* (Fig. 2). Although the maximum intraspecific genetic divergence (3.5% in *S*. (*O*.) *convergens* and *S*. (*O*.) *mediospinosa* Cheng, Xu & Chen, 2010 for *COI* sequences, 3.1% in *S*. (*O*.) *dainuo* for *ND2* sequences) was greater than the minimum interspecific genetic divergence (1.5% and 0.9% in *S*. (*O*.) *aotsukai* and *S*. (*O*.) *nigripennis* for *COI* and *ND2* sequences, respectively), relatively large interspecific divergences (≥ 3.3% and ≥ 4.4% from *COI* and *ND2* sequences, respectively) were observed among the 17 new species (Tables S2 and S3, respectively).

### Taxonomy

**Subgenus *Stegana* (*Oxyphortica*) Duda, 1923**

*Phortica* (*Oxyphortica*) Duda, 1923: 34. Type species: *Drosophila convergens* de Meijere, 1911.

*Chaetocnema* Duda, 1926: 243. Type species: *Chaetocnema poeciloptera* Duda, 1926 (= *Orthostega nigripennis* Hendel, 1914).

*Stegana* (*Oxyphortica*): Okada, 1971: 90; Okada, 1978: 398; Toda & Peng, 1992: 210; Sidorenko, 1998: 297; Xu et al., 2007: 43; Wang et al., 2010: 566.

*Diagnosis*. Mesoscutum with *ca*. 12 irregular rows of acrostichal setulae; first tarsomere of foreleg at least basally and apically, each with two or three black, long, spine-shaped setae on ventral surface; pregonites (parameres in McAlpine, 1981) mostly inconspicuous or absent, if present, usually symmetrical; aedeagus diverse, not annular, occasionally absent; phallapodeme (aedeagal apodeme in McAlpine, 1981) without process subapically.

*Description*. Eyes brownish red. Ocellar triangle black. Postocellar setae divergent, absent in the *convergens* group. Frons brown to dark brown, slightly narrowed below, mostly with sparse interfrontal setulae. Pedicel yellow, with one long seta and several minute setulae; first flagellomere mostly greyish yellow. Face brown. Clypeus narrow, yellow to black. Gena dark brown. Palpus mostly yellow and slender, occasionally dark brown, with several setulae. Mesoscutum yellow to brown, with variable pattern. Postpronotal lobe yellow, with one long seta. Pleuron with a brown longitudinal stripe above (running from propleuron to base of haltere). Katepisternum yellow to brown, with two long and some small setae. Wing greyish brown to dark brown; costal vein between R_2+3_ and R_4+5_ with five to eight peg-like spinules on ventral surface; vein M_1_ distally variably convergent to vein R_4+5_. Haltere brownish on stem, greyish yellow on knob. Legs mainly yellow, with occasional brown tinge or darker brown markings; forefemur with a row of long setae on ventral surface; midtibia with two strong setae on dorsal surface (as Fig. 3D in Zhang et al., 2012). Abdominal tergites yellow to black; sternites mostly yellow. Male terminalia: Cercus mostly lacking pubescence. Surstylus with or without diverse prensisetae. Hypandrial phragma (Hypandrium in McAlpine, 1981) mostly expanded anteriorly. Aedeagal sheaths (Gonopods in McAlpine, 1981) fused, mostly basolaterally extended, fused with lateral gonocoxite (hypandrium in McAlpine, 1981).

**Figure 3:**
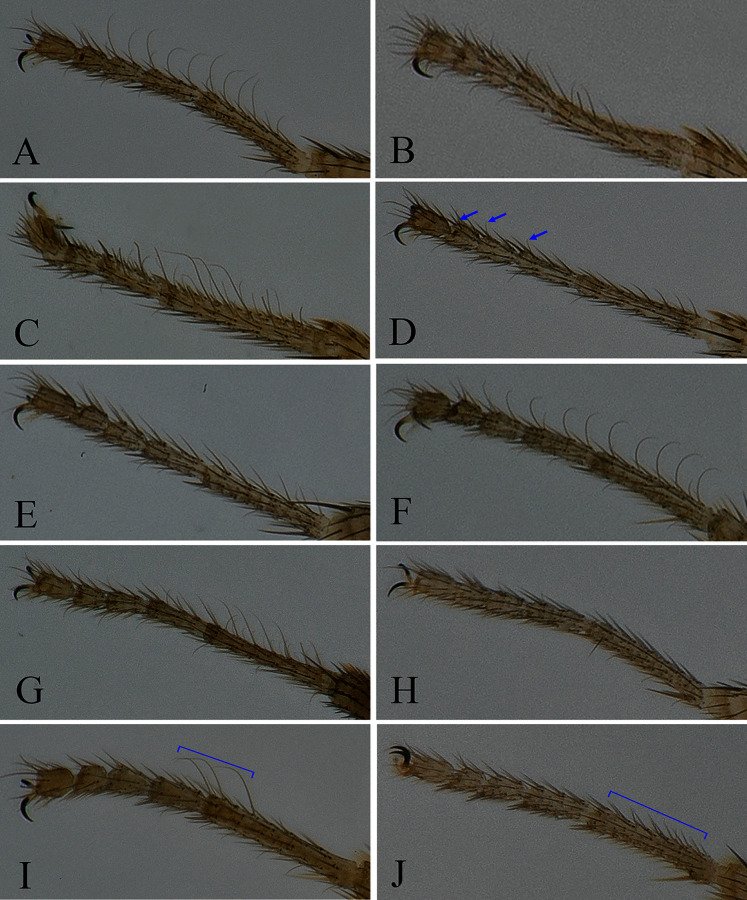
Fore tarsus of male in dorsal view. (A) *Stegana* (*Oxyphortica*) *curvata* Wang, Gao & Chen, 2010; (B) *S*. (*O*.) *wanglei* Wang, Gao & Chen, 2010; (C) *S*. (*O*.) *wuliangi* Wang, Gao & Chen, 2010; (D) *S*. (*O*.) *amphigya* Wang & Chen, sp. nov.; (E) *S*. (*O*.) *armillata* Wang & Chen, sp. nov.; (F) *S*. (*O*.) *ashima* Zhang & Chen, sp. nov.; (G) *S*. (*O*.) *bawo* Zhang & Chen, sp. nov.; (H) *S*. (*O*.) *crypta* Wang & Chen, sp. nov.; (I) *S*. (*O*.) *gelea* Zhang & Chen, sp. nov.; (J) *S*. (*O*.) *hengduanmontana* Zhang & Chen, sp. nov.

Seventeen new species that resemble seven known species (*S*. (*O*.) *crassiforcipata* Wang, Gao & Chen, 2010; *S*. (*O*.) *curvata* Wang, Gao & Chen, 2010; *S*. (*O*.) *enigma* Sidorenko, 1998; *S*. (*O*.) *monoacantha* Wang & Chen, 2010; *S*. (*O*.) *subconvergens* Okada, 1988; *S*. (*O*.) *wanglei* Wang, Gao & Chen, 2010 and *S*. (*O*.) *wuliangi* Wang, Gao & Chen, 2010) in some morphological characteristics (Figs 3–5) (*e.g.*, foretarsi mostly with fringe-like setae on anterior surface; surstylus narrowed and curved, dorsally mostly with one to two stout (occasionally a row of small) prensisetae (Figs 6–22B)) are described.

**Figure 4:**
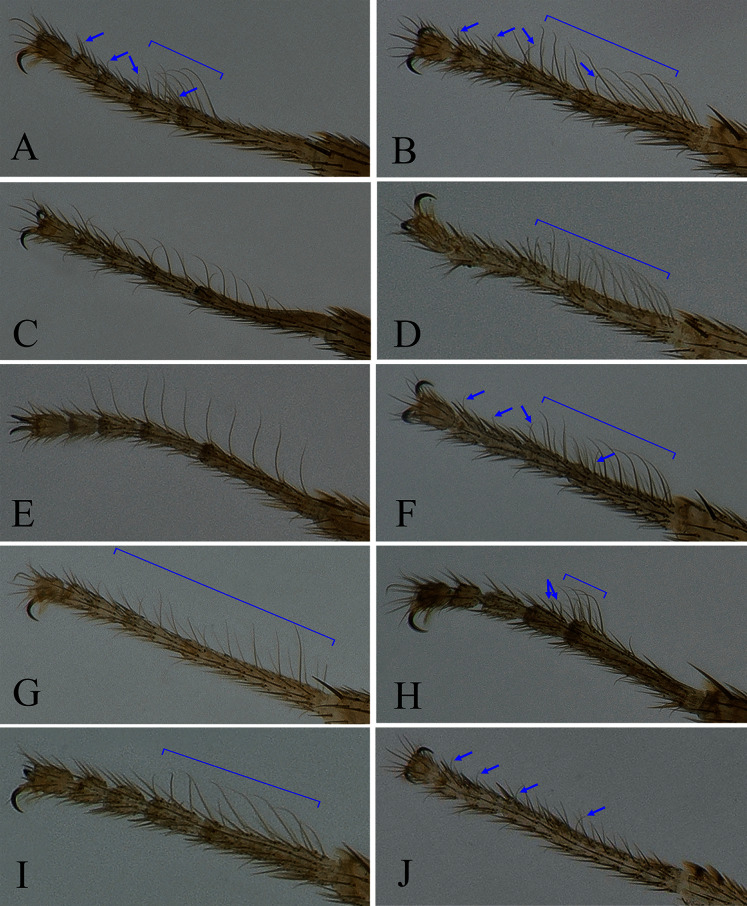
Fore tarsus of male in dorsal view. (A) *Stegana* (*Oxyphortica*) *jinmingi* Zhang & Chen, sp. nov.; (B) *S*. (*O*.) *mengbalanaxi* Zhang & Chen, sp. nov.; (C) *S*. (*O*.) *mouig* Zhang & Chen, sp. nov.; (D) *S*. (*O*.) *setipes* Wang & Chen, sp. nov.; (E) *S*. (*O*.) *shangrila* Zhang & Chen, sp. nov.; (F) *S*. (*O*.) *tsauri* Zhang & Chen, sp. nov.; (G) *S*. (*O*.) *valleculata* Zhang & Chen, sp. nov.; (H) *S*. (*O*.) *wanhei* Zhang & Chen, sp. nov.; (I) *S*. (*O*.) *yangjin* Zhang & Chen, sp. nov.; (J) *S*. (*O*.) *hypophaia* Zhang & Chen, sp. nov.

**Figure 5:**
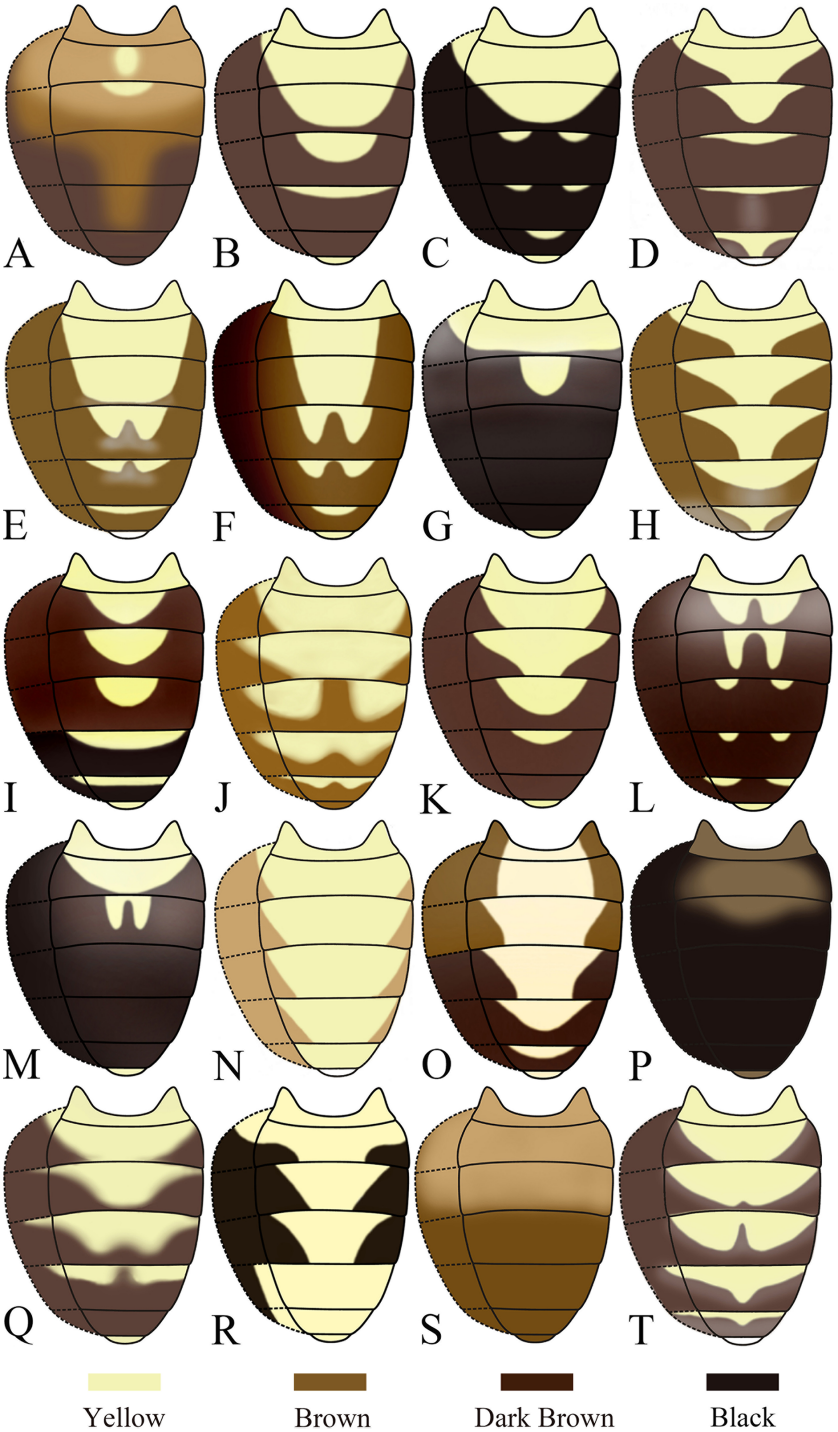
Male abdominal tergites patterns. (A) *Stegana* (*Oxyphortica*) *curvata* Wang, Gao & Chen, 2010; (B) *S*. (*O*.) *wanglei* Wang, Gao & Chen, 2010; (C) *S*. (*O*.) *wuliangi* Wang, & and Chen, 2010; (D) *S*. (*O*.) *amphigya* Wang & Chen, sp. nov.; (E) *S*. (*O*.) *armillata* Wang & Chen, sp. nov.; (F) *S*. (*O*.) *ashima* Zhang & Chen, sp. nov.; (G) *S*. (*O*.) *bawo* Zhang & Chen, sp. nov.; (H) *S*. (*O*.) *crypta* Wang & Chen, sp. nov.; (I) *S*. (*O*.) *gelea* Zhang & Chen, sp. nov.; (J) *S*. (*O*.) *hengduanmontana* Zhang & Chen, sp. nov.; (K) *S*. (*O*.) *jinmingi* Zhang & Chen, sp. nov.; (L) *S*. (*O*.) *mengbalanaxi* Zhang & Chen, sp. nov.; (M) *S*. (*O*.) *mouig* Zhang & Chen, sp. nov.; (N) *S*. (*O*.) *setipes* Wang & Chen, sp. nov.; (O) *S*. (*O*.) *shangrila* Zhang & Chen, sp. nov.; (P) *S*. (*O*.) *tsauri* Zhang & Chen, sp. nov.; (Q) *S*. (*O*.) *valleculata* Zhang & Chen, sp. nov.; (R) *S*. (*O*.) *wanhei* Zhang & Chen, sp. nov.; (S) *S*. (*O*.) *yangjin* Zhang & Chen, sp. nov.; (T) *S*. (*O*.) *hypophaia* Zhang & Chen, sp. nov.

**Figure 6:**
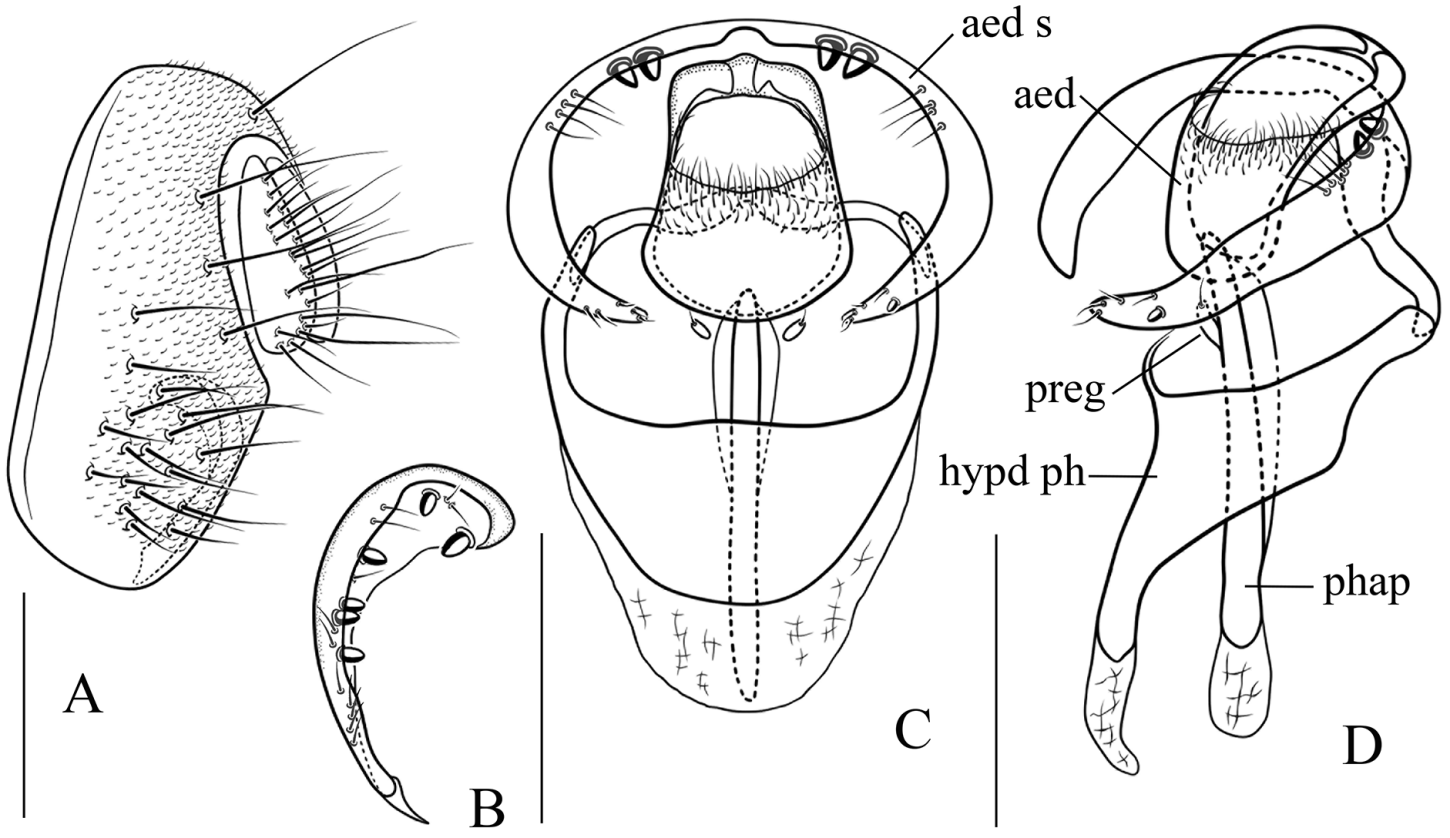
*Stegana* (*Oxyphortica*) *amphigya* Wang & Chen, sp. nov. (A) Epandrium, surstylus and cercus (lateral view); (B) surstylus (ventral view); (C, D) hypandrial phragma (hypd ph), pregonites (preg), aedeagal sheaths (aed s), aedeagus (aed) and phallapodeme (phap; ventral and lateral views). Scale bar = 0.1 mm. Drawing credit: Nan-Nan Wang.

**Figure 7:**
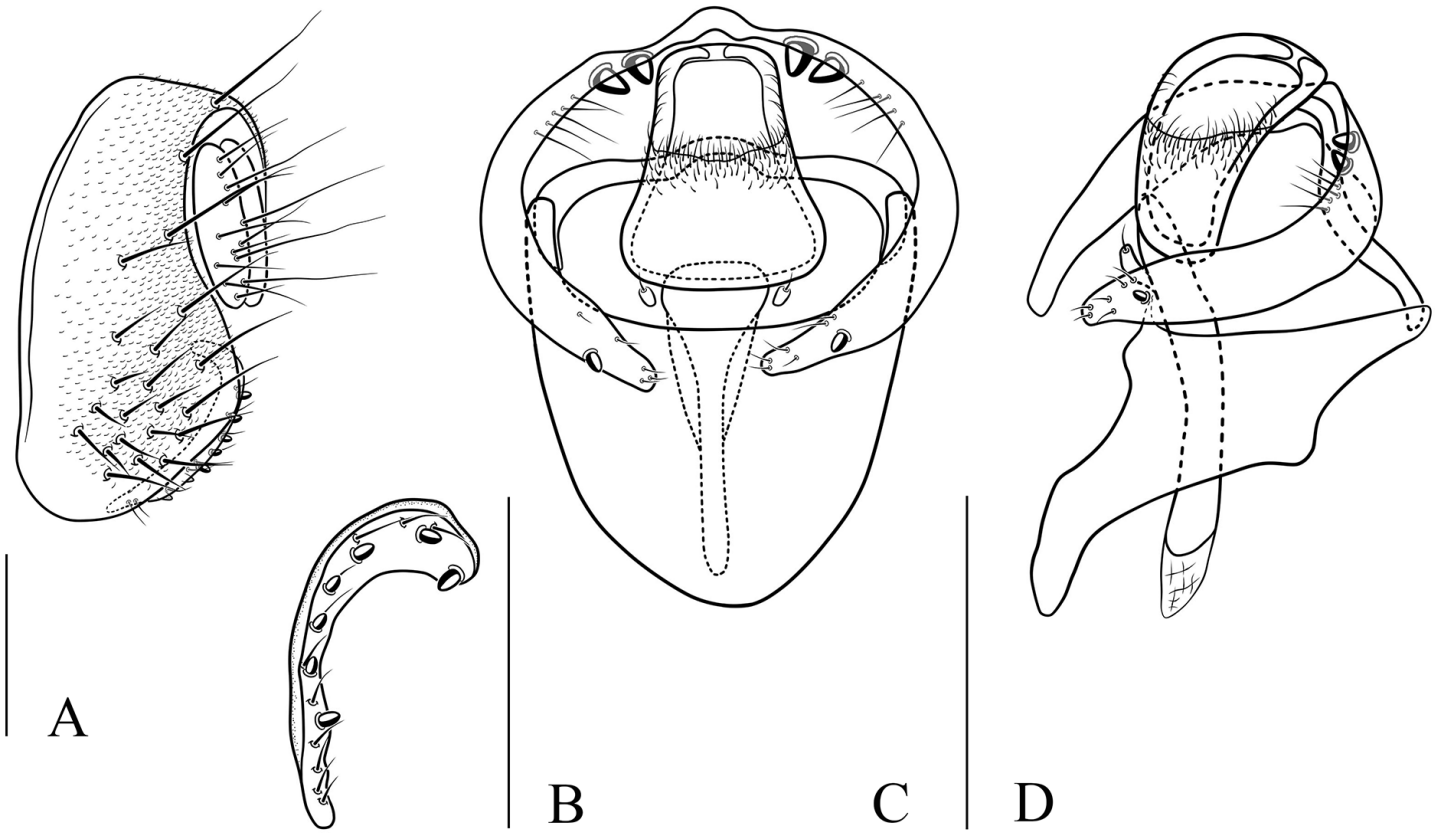
*Stegana* (*Oxyphortica*) *armillata* Wang & Chen, sp. nov. (A) Epandrium, surstylus and cercus (lateral view); (B) surstylus (ventral view); (C, D) hypandrial phragma, pregonites, aedeagal sheaths, aedeagus and phallapodeme (ventral and lateral views). Scale bar = 0.1 mm. Drawing credit: Nan-Nan Wang.

**Figure 8:**
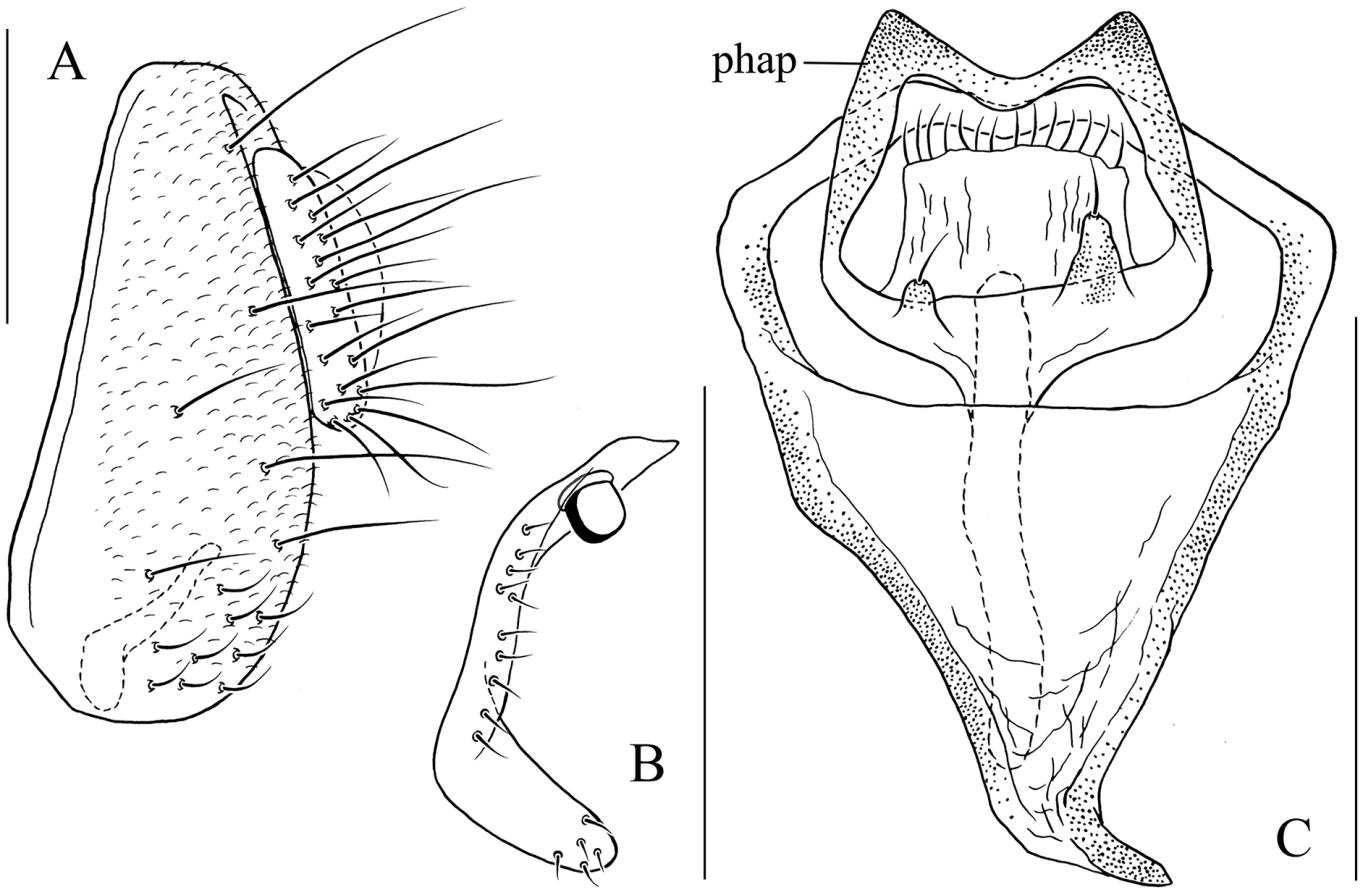
*Stegana* (*Oxyphortica*) *ashima* Zhang & Chen, sp. nov. (A) Epandrium, surstylus and cercus (lateral view); (B) surstylus (ventral view); (C) hypandrial phragma, pregonites, aedeagal sheaths, aedeagus and phallapodeme (ventral view). Scale bar = 0.1 mm. Drawing credit: Yuan Zhang.

**Figure 9:**
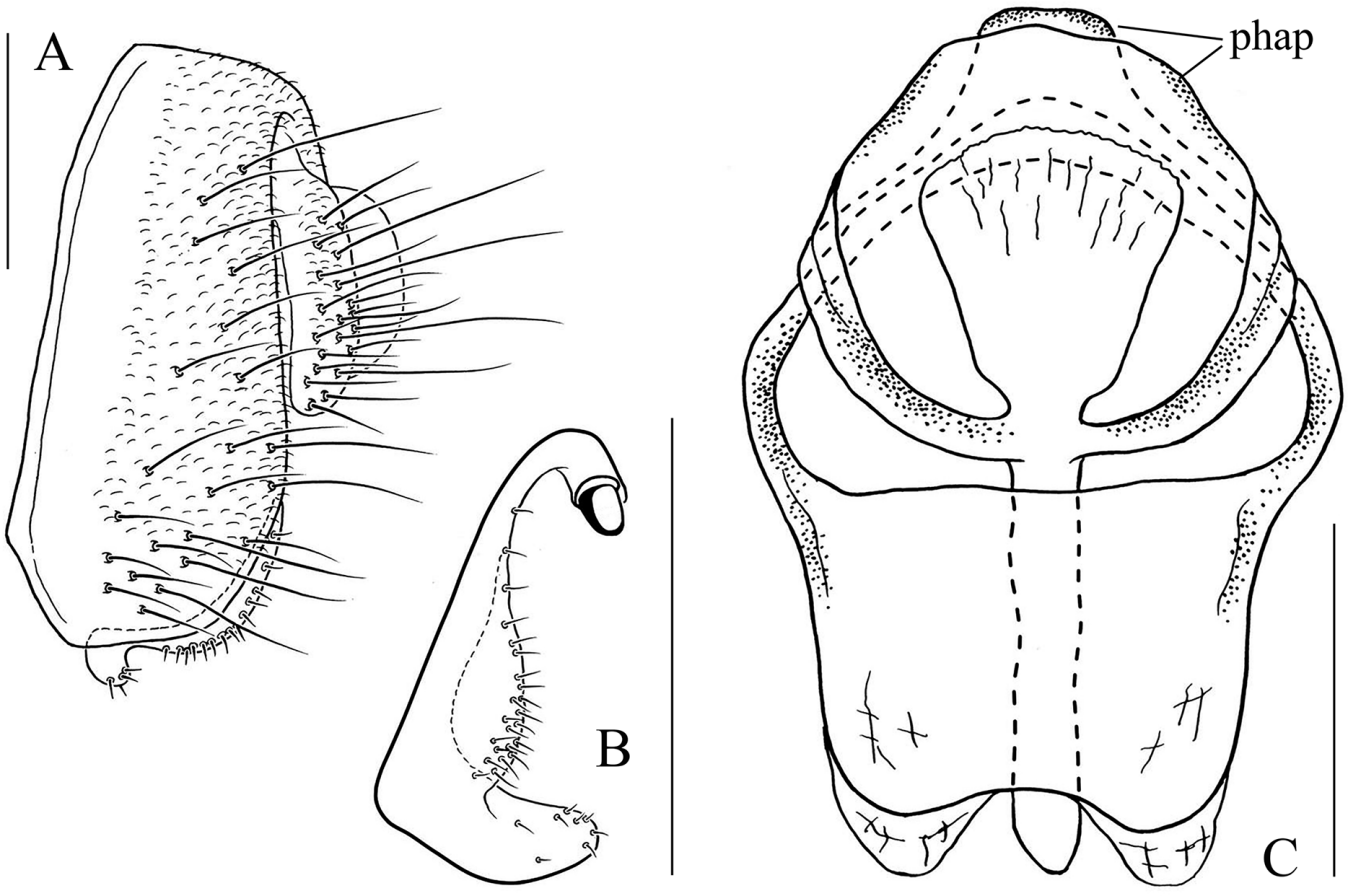
*Stegana* (*Oxyphortica*) *bawo* Zhang & Chen, sp. nov. (A) Epandrium, surstylus and cercus (lateral view); (B) surstylus (ventral view); (C) hypandrial phragma, aedeagal sheaths, aedeagus and phallapodeme (ventral view). Scale bar = 0.1 mm. Drawing credit: Yuan Zhang.

**Figure 10:**
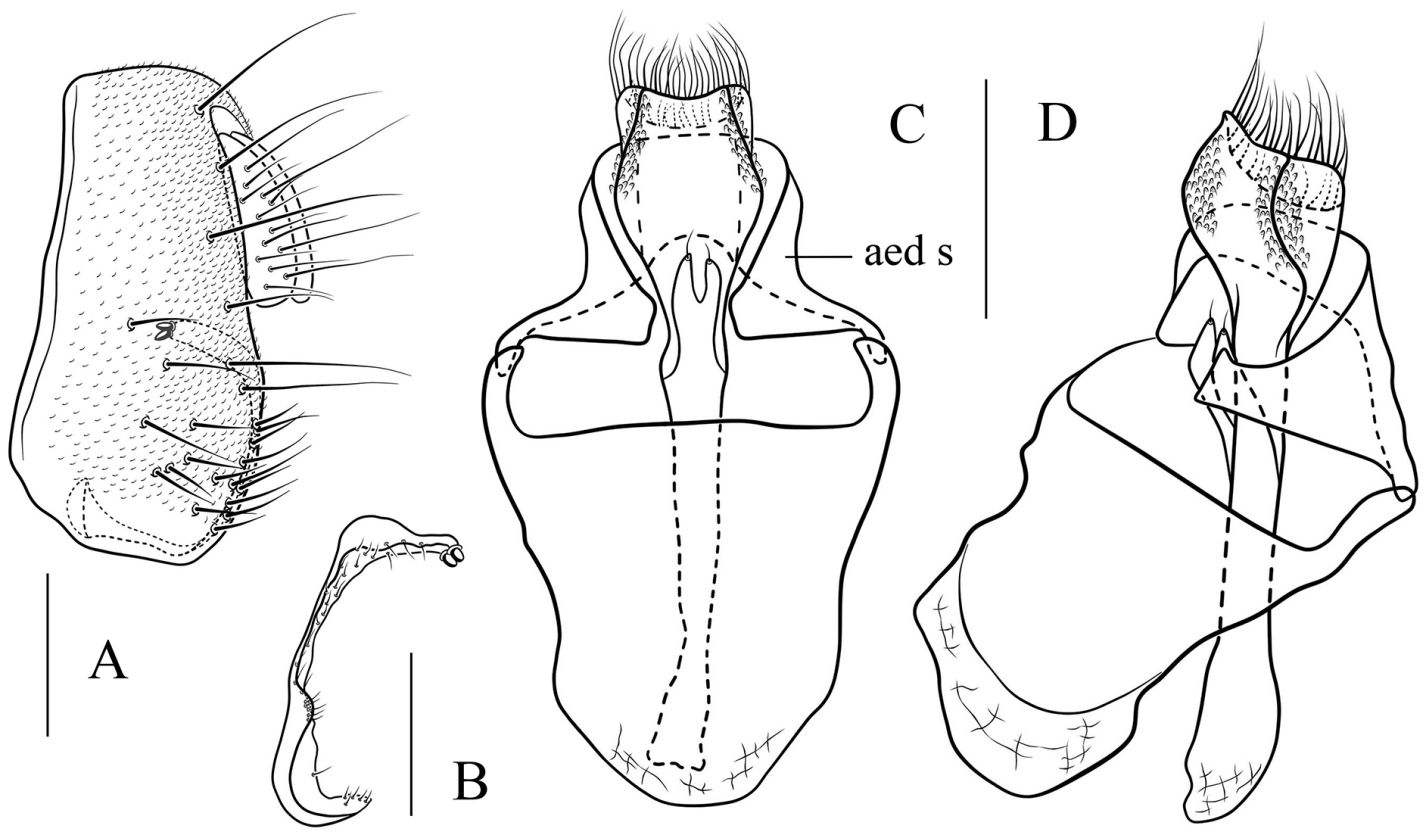
*Stegana* (*Oxyphortica*) *crypta* Zhang & Chen, sp. nov. (A) Epandrium, surstylus and cercus (lateral view); (B) surstylus (ventral view); (C, D) hypandrial phragma, pregonites, aedeagal sheaths, aedeagus and phallapodeme (ventral and lateral views). Scale bar = 0.1 mm. Drawing credit: Yuan Zhang.

**Figure 11:**
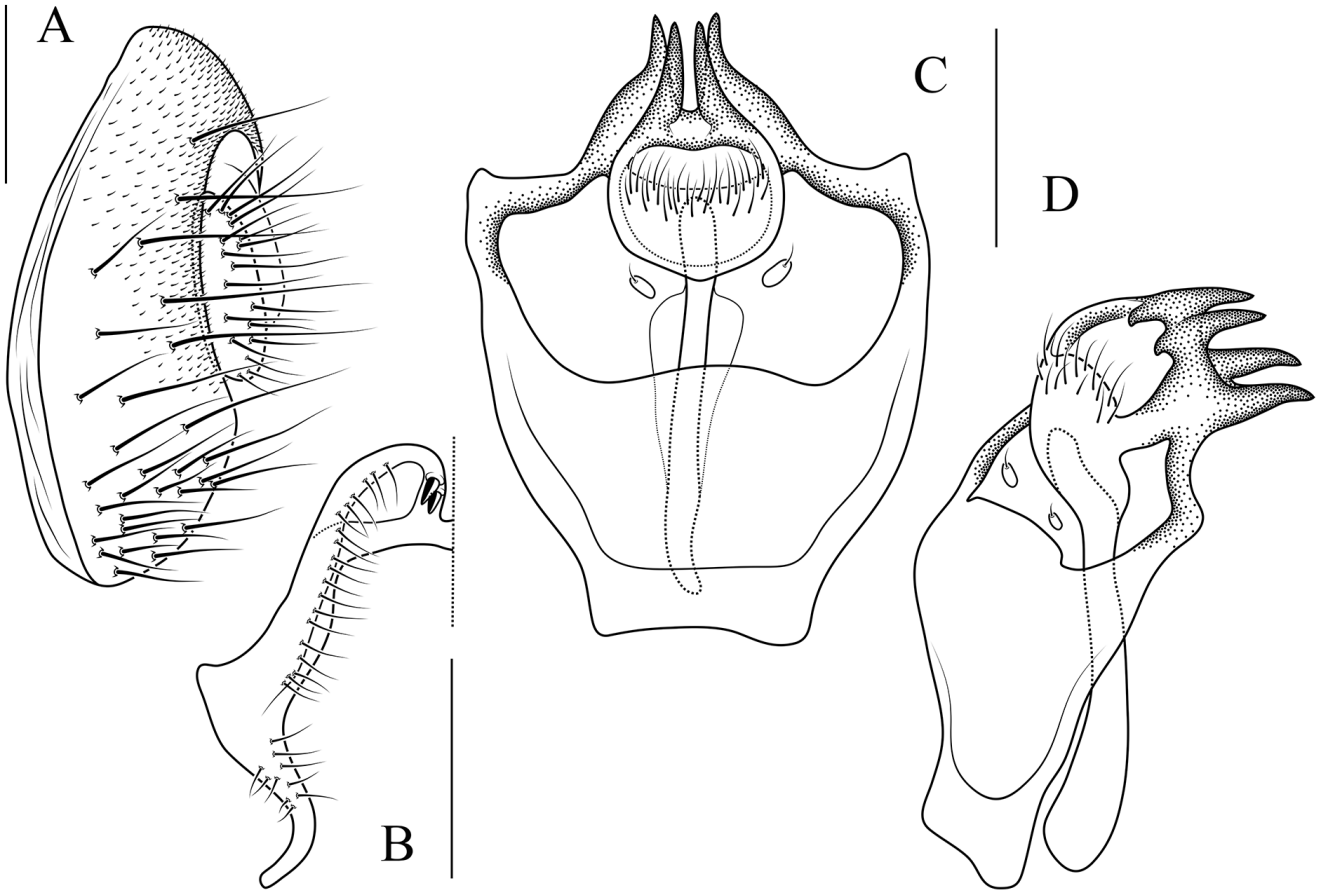
*Stegana* (*Oxyphortica*) *gelea* Zhang & Chen, sp. nov. (A) Epandrium, surstylus and cercus (lateral view); (B) surstylus (ventral view); (C, D) hypandrial phragma, pregonites, aedeagal sheaths, aedeagus and phallapodeme (ventral and lateral views). Scale bar = 0.1 mm. Drawing credit: Yuan Zhang.

**Figure 12:**
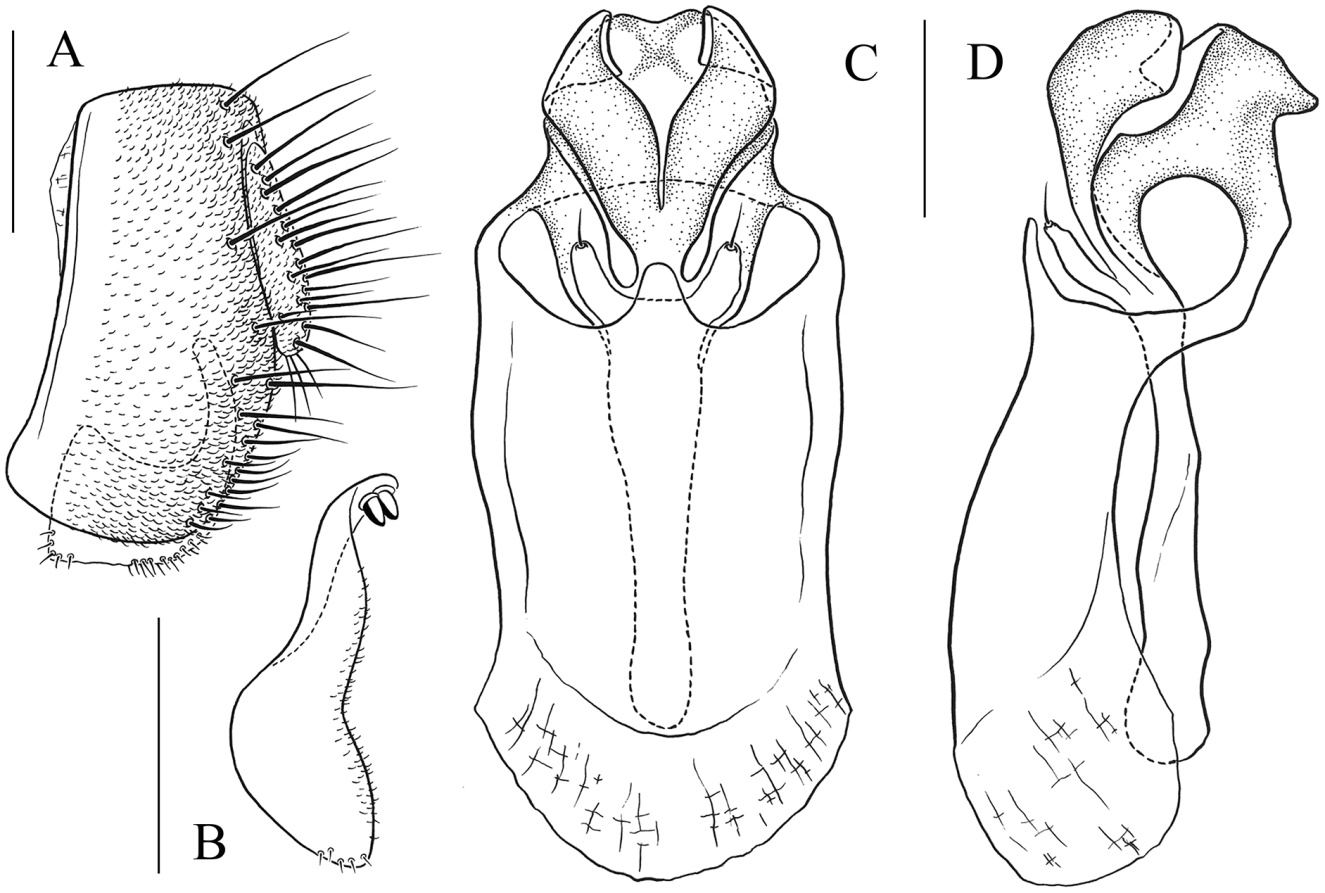
*Stegana* (*Oxyphortica*) *hengduanmontana* Zhang & Chen, sp. nov. (A) Epandrium, surstylus and cercus (lateral view); (B) surstylus (ventral view); (C, D) hypandrial phragma, pregonites, aedeagal sheaths, aedeagus and phallapodeme (ventral views). Scale bar = 0.1 mm. Drawing credit: Yuan Zhang.

**Figure 13:**
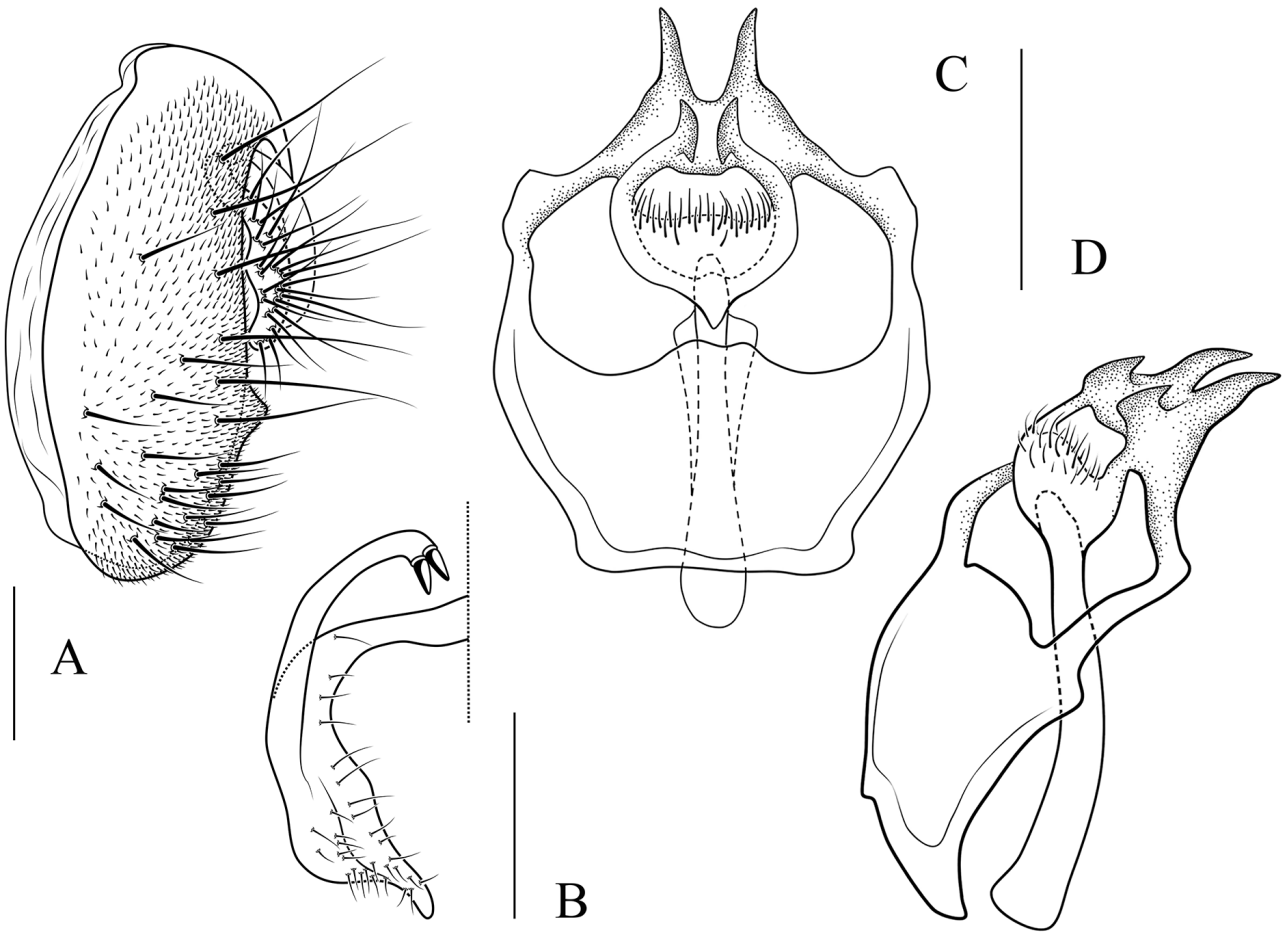
*Stegana* (*Oxyphortica*) *jinmingi* Zhang & Chen, sp. nov. (A) Epandrium, surstylus and cercus (lateral view); (B) surstylus (ventral view); (C, D) hypandrial phragma, aedeagal sheaths, aedeagus and phallapodeme (ventral views). Scale bar = 0.1 mm. Drawing credit: Yuan Zhang.

**Figure 14:**
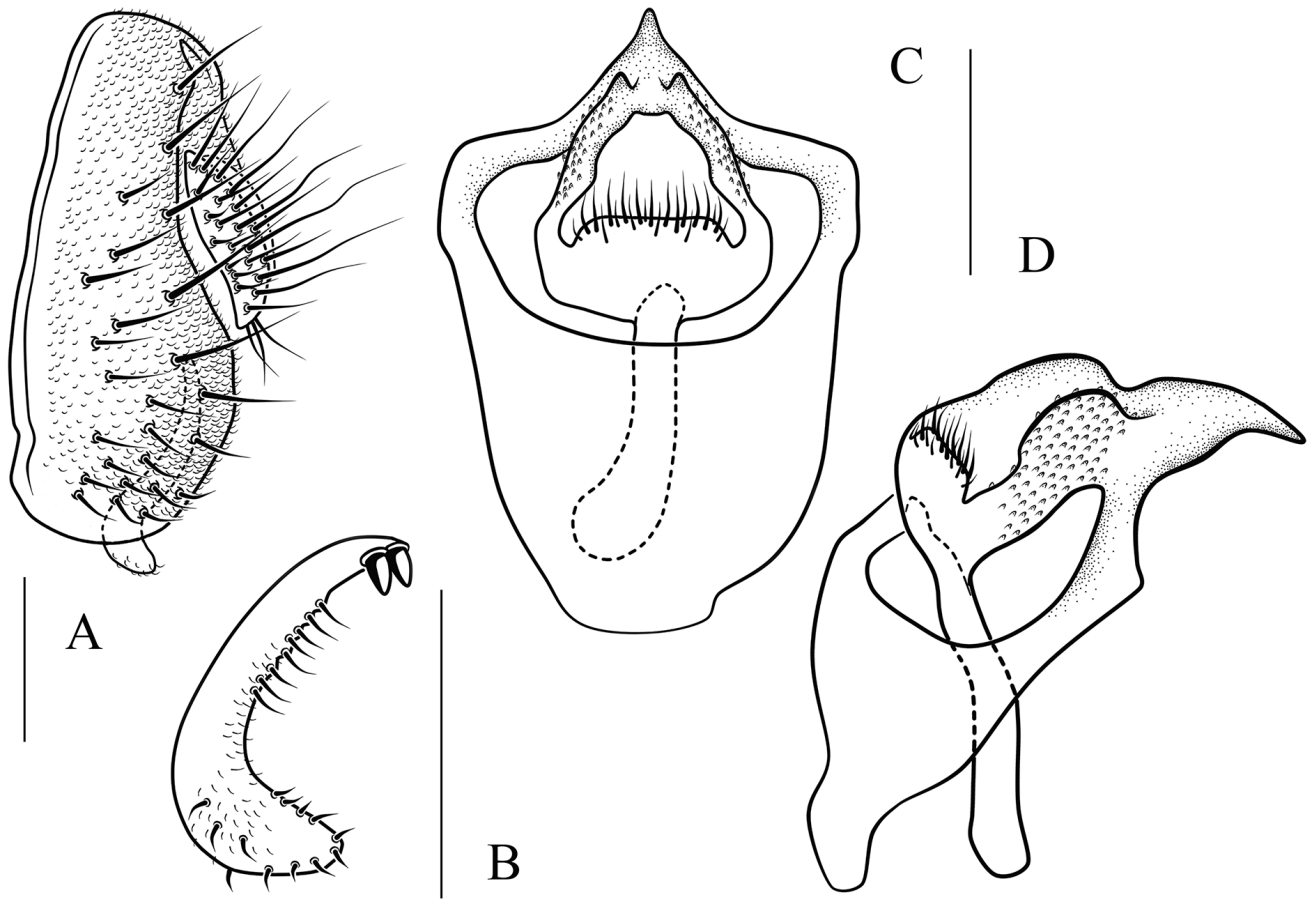
*Stegana* (*Oxyphortica*) *mengbalanaxi* Zhang & Chen, sp. nov. (A) Epandrium, surstylus and cercus (lateral view); (B) surstylus (ventral view); (C, D) hypandrial phragma, pregonites, aedeagal sheaths, aedeagus and phallapodeme (ventral views). Scale bar = 0.1 mm. Drawing credit: Yuan Zhang.

**Figure 15:**
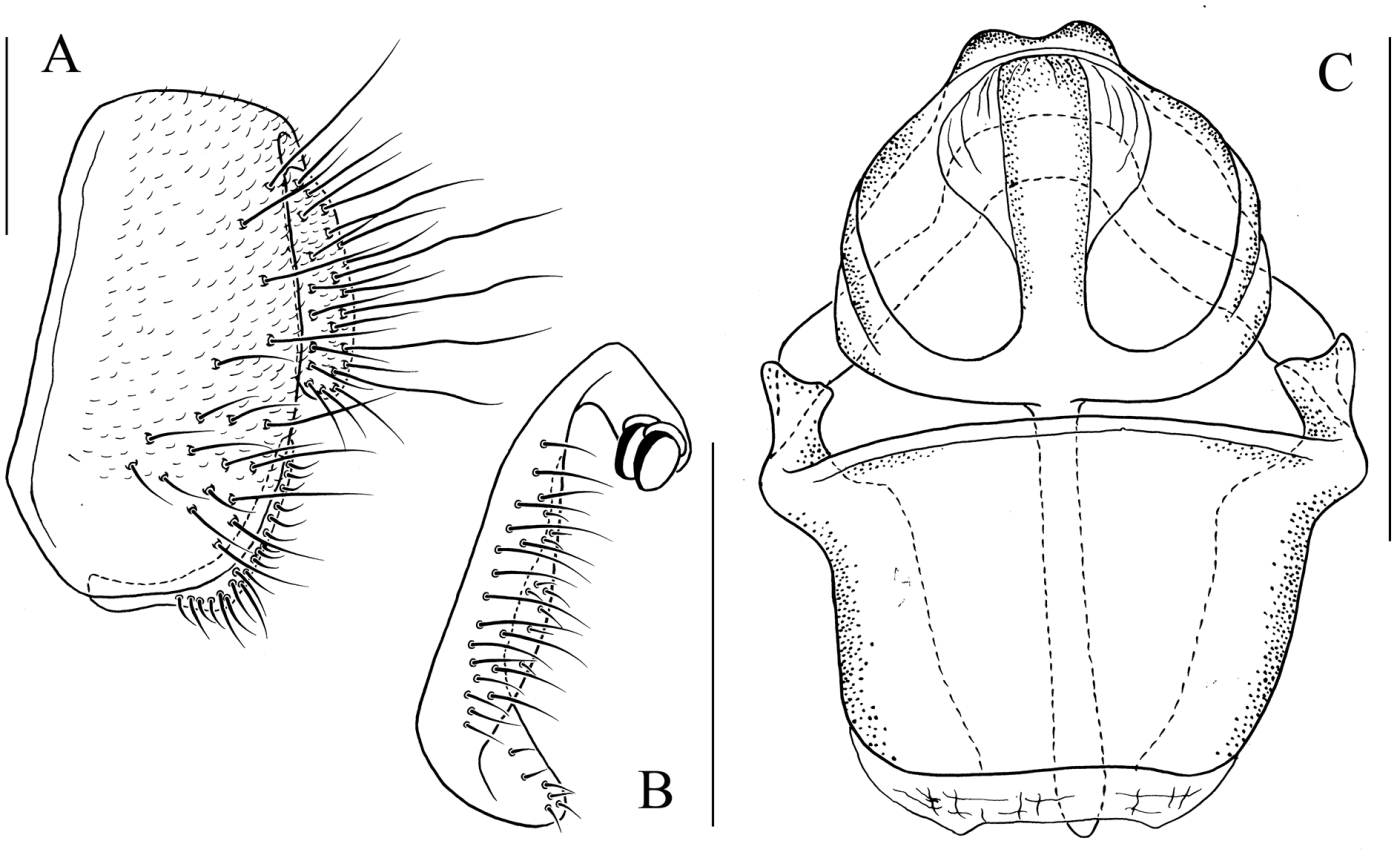
*Stegana* (*Oxyphortica*) *mouig* Zhang & Chen, sp. nov. (A) Epandrium, surstylus and cercus (lateral view); (B) surstylus (ventral view); (C) hypandrial phragma, aedeagal sheaths, aedeagus and phallapodeme (ventral view). Scale bar = 0.1 mm. Drawing credit: Yuan Zhang.

**Figure 16:**
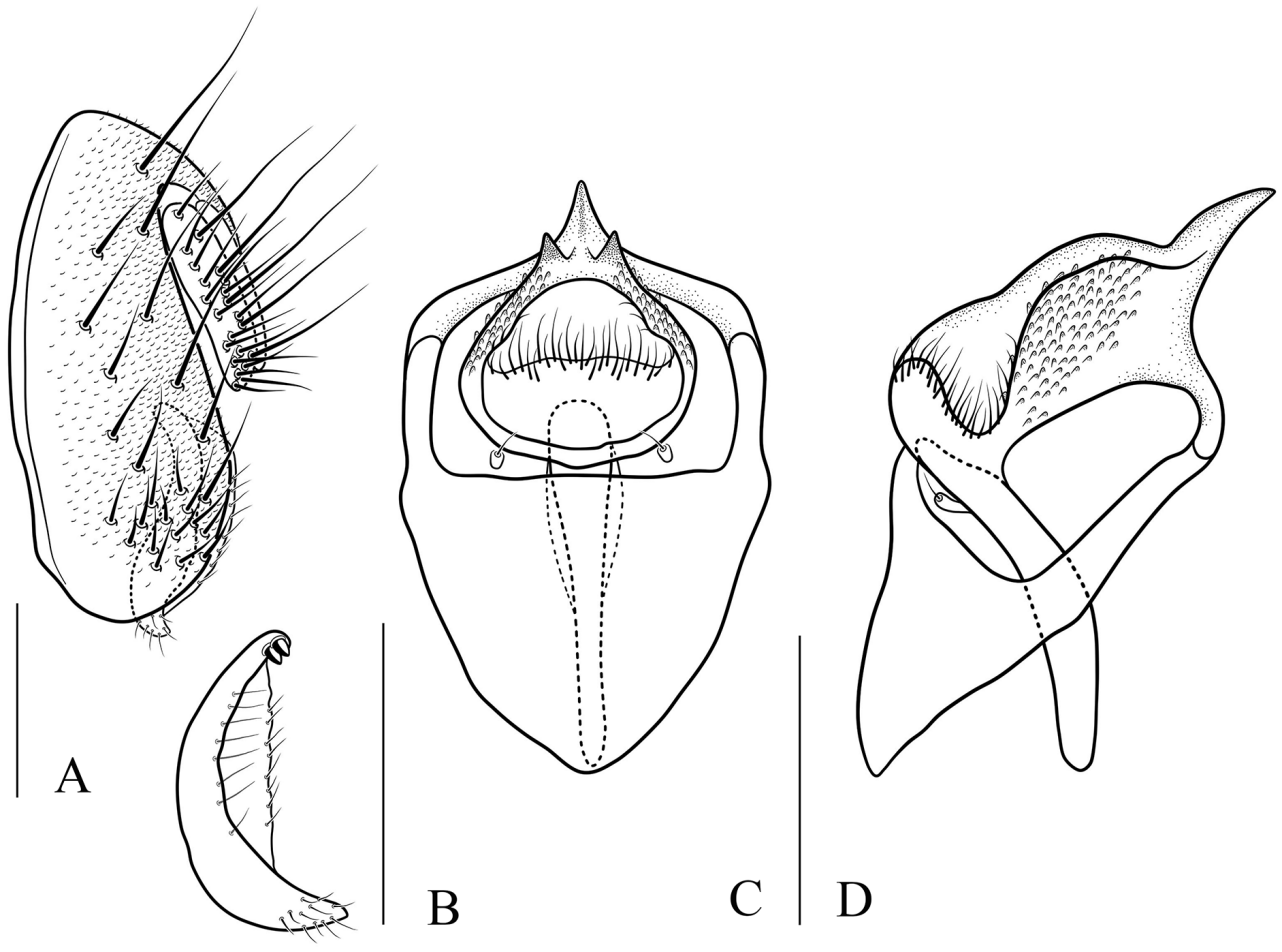
*Stegana* (*Oxyphortica*) *setipes* Zhang & Chen, sp. nov. (A) Epandrium, surstylus and cercus (lateral view); (B) surstylus (ventral view); (C, D) hypandrial phragma, pregonites, aedeagal sheaths, aedeagus and phallapodeme (ventral views). Scale bar = 0.1 mm. Drawing credit: Yuan Zhang.

**Figure 17:**
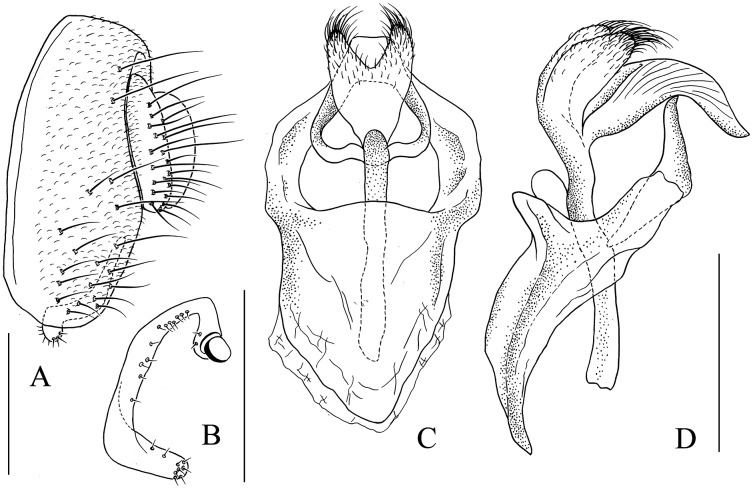
*Stegana* (*Oxyphortica*) *shangrila* Zhang & Chen, sp. nov. (A) Epandrium, surstylus and cercus (lateral view); (B) surstylus (ventral view); (C, D) hypandrial phragma, aedeagal sheaths, aedeagus and phallapodeme (ventral views). Scale bar = 0.1 mm. Drawing credit: Yuan Zhang.

**Figure 18:**
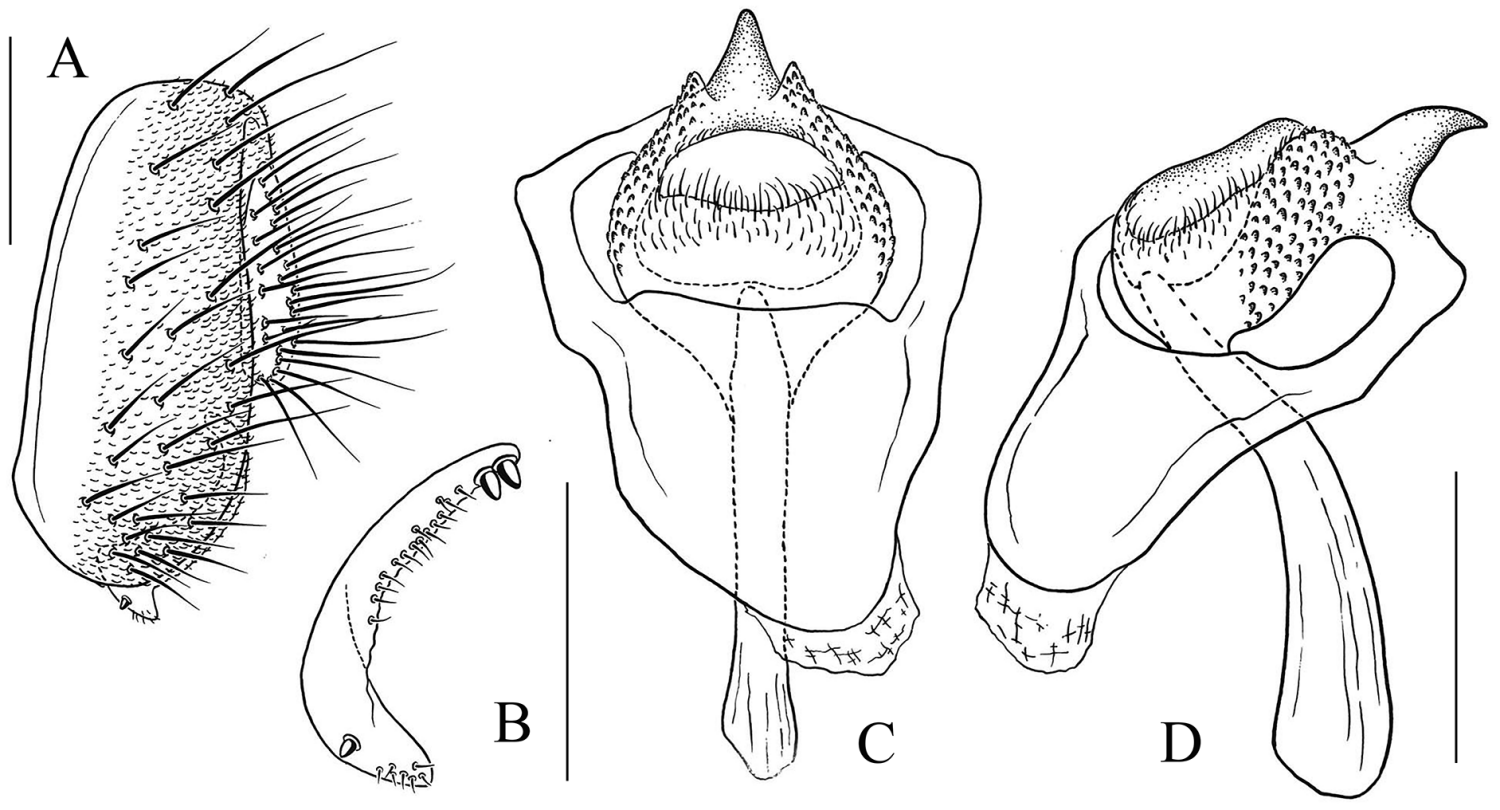
*Stegana* (*Oxyphortica*) *tsauri* Zhang & Chen, sp. nov. (A) Epandrium, surstylus and cercus (lateral view); (B) surstylus (ventral view); (C, D) hypandrial phragma, aedeagal sheaths, aedeagus and phallapodeme (ventral views). Scale bar = 0.1 mm. Drawing credit: Yuan Zhang.

**Figure 19:**
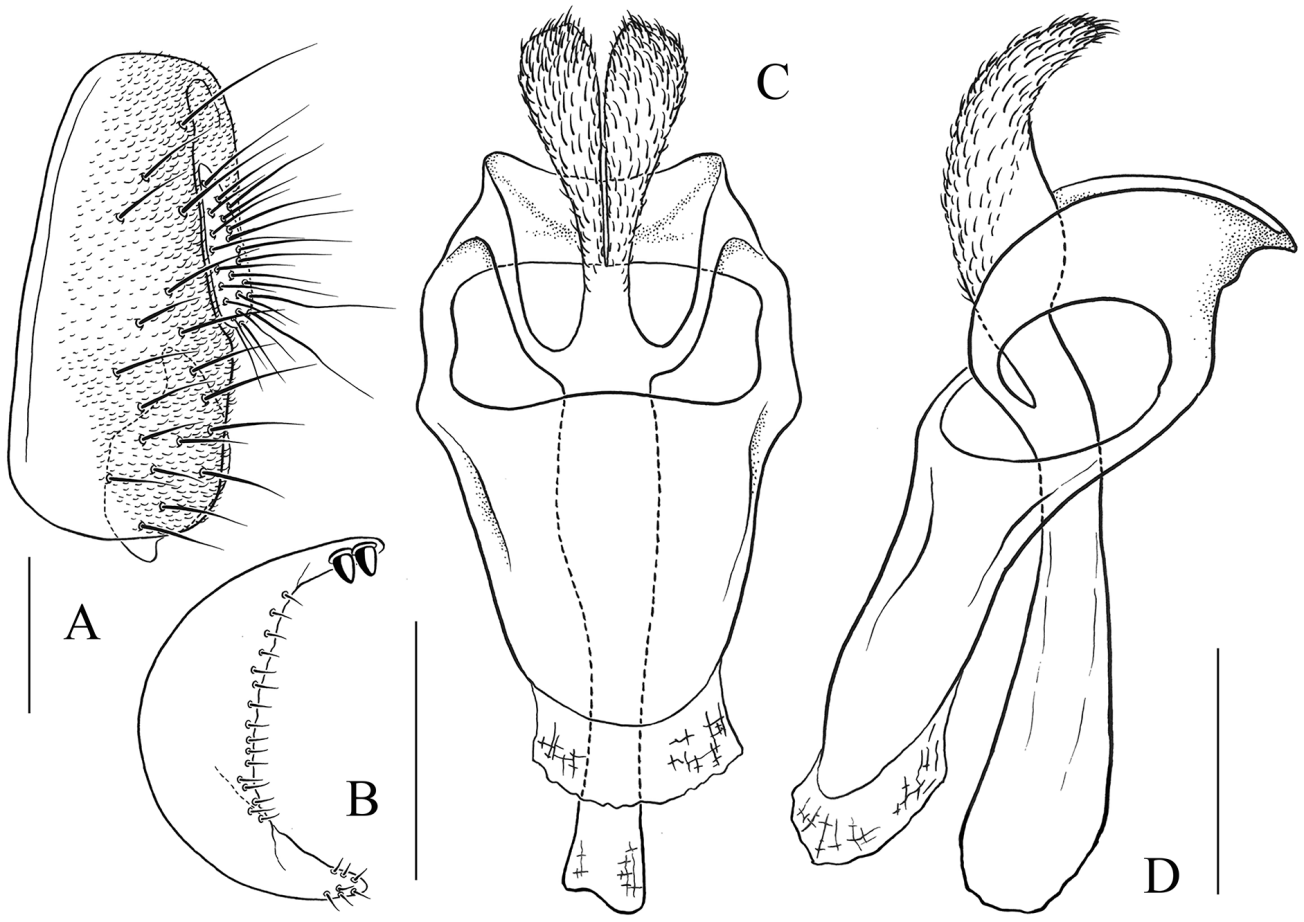
*Stegana* (*Oxyphortica*) *valleculata* Zhang & Chen, sp. nov. (A) Epandrium, surstylus and cercus (lateral view); (B) surstylus (ventral view); (C, D) hypandrial phragma, aedeagal sheaths, aedeagus and phallapodeme (ventral views). Scale bar = 0.1 mm. Drawing credit: Yuan Zhang.

**Figure 20:**
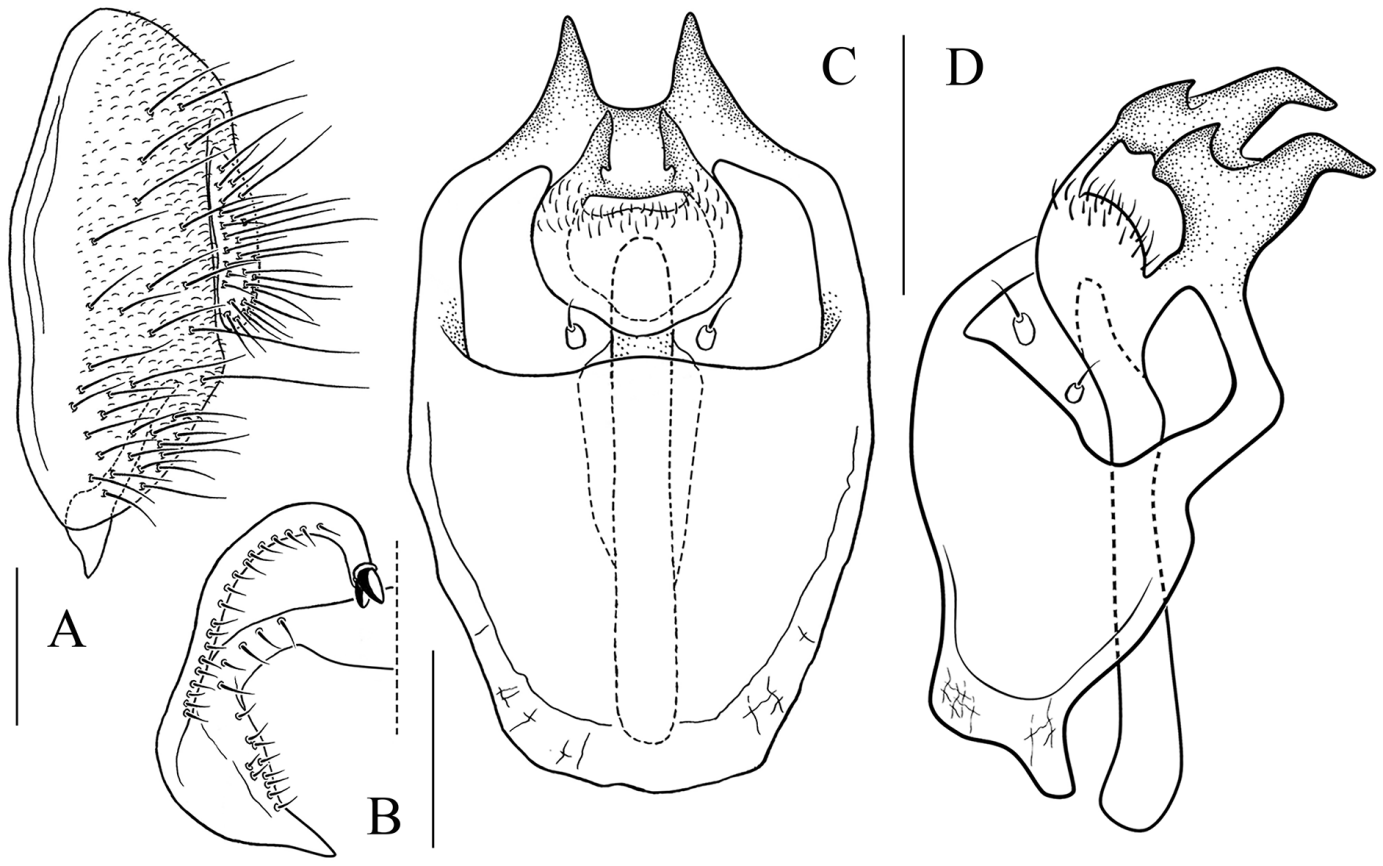
*Stegana* (*Oxyphortica*) *wanhei* Zhang & Chen, sp. nov. (A) Epandrium, surstylus and cercus (lateral view); (B) surstylus (ventral view); (C, D) hypandrial phragma, pregonites, aedeagal sheaths, aedeagus and phallapodeme (ventral views). Scale bar = 0.1 mm. Drawing credit: Yuan Zhang.

**Figure 21:**
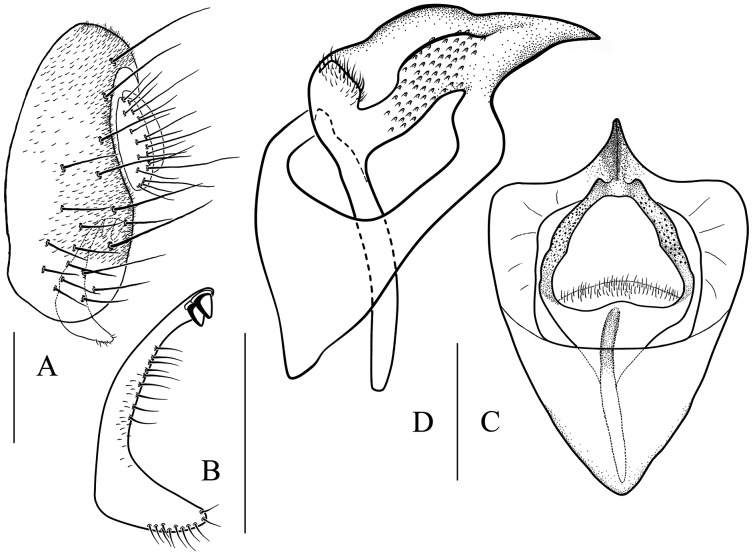
*Stegana* (*Oxyphortica*) *yangjin* Zhang & Chen, sp. nov. (A) Epandrium, surstylus and cercus (lateral view); (B) surstylus (ventral view); (C, D) hypandrial phragma, pregonites, aedeagal sheaths, aedeagus and phallapodeme (ventral views). Scale bar = 0.1 mm. Drawing credit: Yuan Zhang.

**Figure 22:**
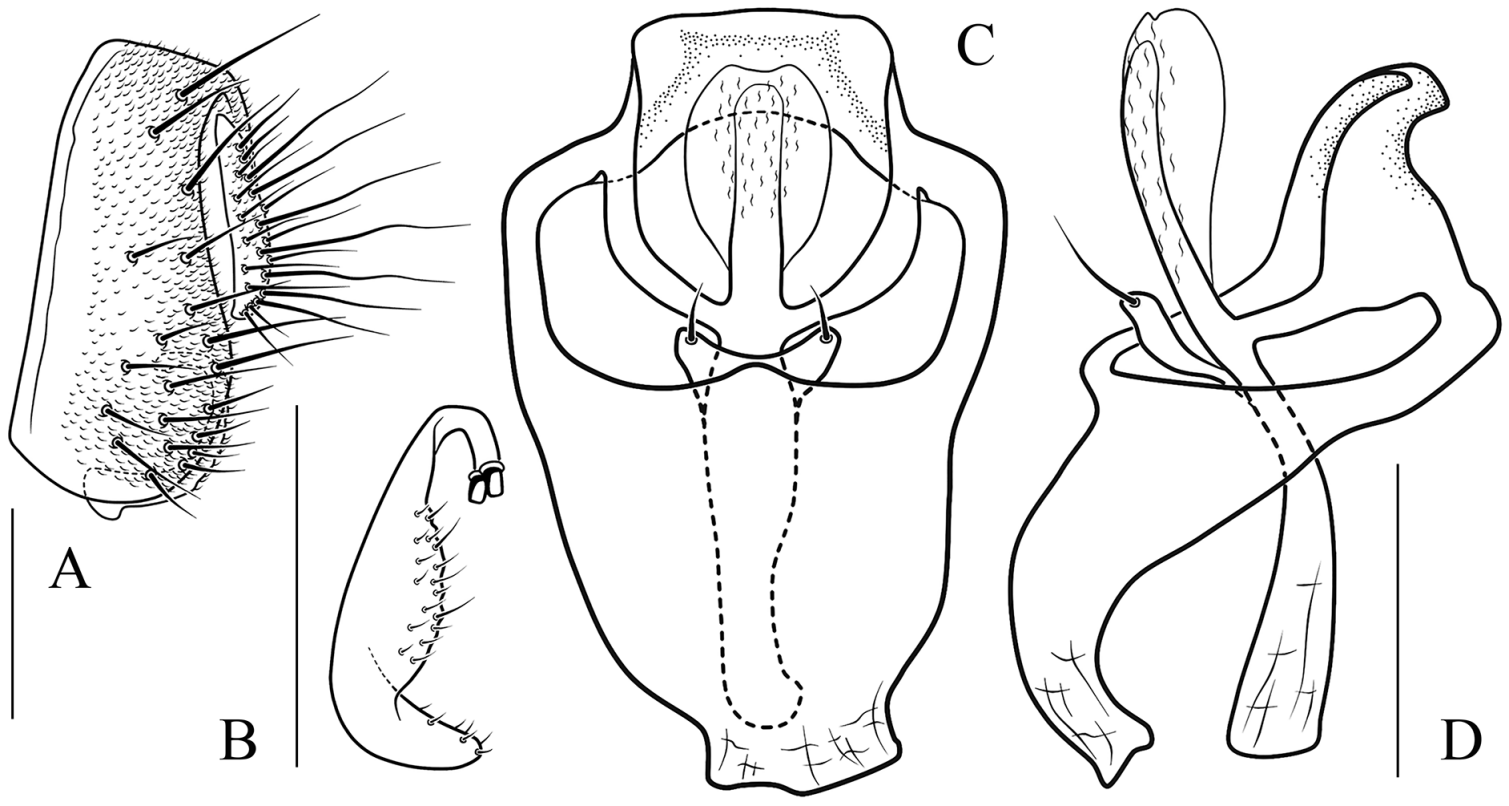
*Stegana* (*Oxyphortica*) *hypophaia* Zhang & Chen, sp. nov. (A) Epandrium, surstylus and cercus (lateral view); (B) surstylus (ventral view); (C, D) hypandrial phragma, pregonites, aedeagal sheaths, aedeagus and phallapodeme (ventral views). Scale bar = 0.1 mm. Drawing credit: Yuan Zhang.

***Stegana* (*Oxyphortica*) *amphigya* Wang & Chen, sp. nov.**

urn:lsid:zoobank.org:act:E6BE14A6-5E2B-4DAD-87D6-2819DFAE5202

(Figs 3D, 5D, 6)

*Type specimens. Holotype*, ♂ (SCAU, no. 111283), CHINA: Mengdong, Cangyuan, Yunnan, 23°100′8″N, 99°13′52″E, 1,320–1,400 m a.s.l. (above sea level), 6.v.2016, ex *tussock* (*J Huang*). *Paratypes*, CHINA: 5♂, 1♀ (SCAU, nos 111284–89), ex *tussocks and tree trunks*, same data as holotype (*Y. Liu*, *Y. Wang*, *L Zhu*); 1♂, 1♀ (KIZ, nos 0090651, 52), Baihualing, Baoshan, Yunnan, 25°46′02″N, 98°43′44″E, 1,400–1,600 m a.s.l., 23.viii.2013, ex *tussock* (*J. Gao*); 6♂, 8♀ (SCAU, nos 111290–303), Husa, Longchuan, Yunnan, 24°27′37″N, 97°45′9″E, 1,230–1,340 m a.s.l., 20.viii.2016, ex *tree trunks* (*H. Chen*, *L Gong*, *Y. Liu*).

*Diagnosis*. Foretarsi second to fourth subapically, each with one small, suberect, fringe-like seta on anterior surface (Fig. 3D). Surstylus acute ventrally, with six prensisetae on dorstal half (Fig. 6B); aedeagal sheaths submedially with two pairs of prensisetae and four pairs of setae, distolaterally strongly, elongated to semiring-shaped, with a few setae distally and one small prensisetae on one side (Figs. 6C, 6D).

*Description*. Arista with four to six dorsal and three to five ventral branches. Clypeus yellowish brown. Mesoscutum and scutellum yellow to yellowish brown. Katepisternum brown above, yellow below. Abdominal first tergite yellow; second to sixth tergites mostly dark brown, yellow medially, narrowly yellow bands on anterior margins on fourth and fifth tergites (Fig. 5D). Sternites mostly brown, yellow on second. Male terminalia (Fig. 6): Epandrium with *ca*. 20 setae on each posterior portion. Surstylus with a row of *ca*. 11 setae. Pregonites small and inconspicuous. Aedeagus with pubescence distally.

*Measurements*. Body length (BL) = 2.83 mm in holotype (5♂ and 5♀ paratypes: 2.16–2.67 in ♂, 2.53–3.20 in ♀), thorax length (THL) = 1.17 mm (0.93–1.17, 1.07–1.37), wing length (WL) = 2.33 mm (1.83–2.23, 2.17–2.70), wing width (WW) = 1.07 mm (0.93–1.07, 0.93–1.30).

*Etymology*. From the Greek word: *amphigyos*, referring to the surstylus pointed on both sides.

***Stegana* (*Oxyphortica*) *armillata* Wang & Chen, sp. nov.**

urn:lsid:zoobank.org:act:699B1E1E-35F2-4772-A36E-6BE7277C4575

(Figs. 3E, 5E, 7)

*Type specimens. Holotype*, ♂ (SCAU, no. 111276), CHINA: Dashuigou, Lüchun, Yunnan, 22°57′19″N, 101°57′04″E, 1,310 m a.s.l., 2.viii.2016, ex *tussock* (*H. Chen*). *Paratypes*, CHINA: 6♂, 3♀ (2♂, 1♀ in KIZ, nos 0090648–50; 4♂, 2♀ in SCAU, nos 111277–82), Sanmeng, Lüchun, Yunnan, 22°53′25″N, 102°19′00″E, 1,560 m a.s.l., 1.viii.2016, ex *tussocks* (*H. Chen*, *L Gong*, *Y. Liu*).

*Diagnosis*. This species is very similar to *S*. (*O*.) *amphigya* **sp. nov.** in male terminalia, but can be distinguished by the foretarsi second to fourth subapically lacking small, suberect, fringe-like seta on anterior surface (Fig. 3E); surstylus round ventrally, with seven small prensisetae on dorstal 3/4 (Fig. 7B).

*Description*. Arista with four to six dorsal and three to four ventral branches. Clypeus brown. Mesoscutum and scutellum yellow to brownish yellow. Katepisternum brownish above, yellow below. Abdominal first tergite yellow; second and third tergites yellow medially, brown laterally; fourth to sixth tergites mostly brown, yellow on anterior margins (Fig. 5E). Sternites yellow, with narrowly brown band on lateral margins of fourth to sixth. Male terminalia (Fig. 7): Epandrium with *ca*. 21 setae on each posterior portion. Surstylus with a row of *ca*. 12 setae. Aedeagal sheaths submedially with two pairs of prensisetae and five pairs of setae, elongated to semiring-shaped, distally with one small prensisetae and a few setae on each side. Pregonites small, inconspicuous. Aedeagus with pubescence distally.

*Measurements*. BL = 2.43 mm in holotype (5♂ and 3♀ paratypes: 2.40–3.33 in ♂, 2.60–3.38 in ♀), THL = 1.07 mm (0.93–1.33, 1.07–1.51), WL = 2.17 mm (1.87–2.57, 2.20–2.83), WW = 1.03 mm (0.87–1.23, 0.97–1.37).

*Etymology*. From the Latin word: *armillatus*, referring to the semiorbicular surstylus.

***Stegana* (*Oxyphortica*) *ashima* Zhang & Chen, sp. nov.**

urn:lsid:zoobank.org:act:5F7EECB0-12D6-419F-90BB-697D9BF2C455

(Figs. 3F, 5F, 8)

*Type specimens. Holotype*, ♂ (SCAU, no. 121350), CHINA: Cangshan, Yangbi, Yunnan, 25°38′22″N, 100°01′55″E, 1,800 m a.s.l., 26.viii.2011, ex *tussock* (*H. Chen*). *Paratypes*, CHINA: 4♂, 4♀ (2♂, 1♀ in KIZ, nos 0090645–47; 2♂, 3♀ in SCAU, nos 121351–55), same data as holotype (*H. Chen*, *J. Gao*).

*Diagnosis*. Epandrium with *ca*. six long and nine short setae on posterior portion per side (Fig. 8A); pregonites asymmetric (Fig. 8C); phallapodeme anterodistally developed, annularly fused and deeply concave posteromedially (Fig. 8C, phap); aedeagus with a row of setae on apical margin (Fig. 8C).

*Description*. Arista with four to six dorsal and three to four ventral branches. Clypeus black. Palpus brown. Mesoscutum brownish yellow to brown. Katepisternum brown, sometimes yellow below. Scutellum brown. Foretarsi first to third with *ca*. 12 valgus fringe-like setae on anterior surface (Fig. 3F). Abdominal first to third tergites yellow medially, brown to black laterally; fourth to sixth tergites mostly brown, yellow dorsally along anterior margins (Fig. 5E). Sternites brownish yellow. Male terminalia (Fig. 8): Surstylus with one stout prensisetae subdorsally, a row of *ca*. 10 setae medially and five setae ventrally. Hypandrial phragma narrowed anteriorly. Aedeagal sheath slender.

*Measurements*. BL = 2.30 mm in holotype (4♂ and 3♀ paratypes: 1.83–2.50 in ♂, 2.07–2.17 in ♀), THL = 0.93 mm (0.83–1.07, 0.83–0.90), WL = 2.00 mm (2.00–2.50, 2.00–2.30), WW = 0.83 mm (0.83–1.00, 0.83–0.87).

*Etymology*. Name of a beautiful woman from legend of the Yi ethnic group in Yunnan, China.

***Stegana* (*Oxyphortica*) *bawo* Zhang & Chen, sp. nov.**

urn:lsid:zoobank.org:act:7EF83FD7-92D3-4831-BBA6-3ACDF305E9D0

(Figs. 3G, 5G, 9)

*Type specimens. Holotype*, ♂ (SCAU, no. 121365), CHINA: Hutiaoxia, Xianggelila, Yunnan, 27°11′24″N, 100°02′54″E, 1,700 m a.s.l., 22.viii.2011, ex *tussock* (*H. Chen*). *Paratype*, CHINA: 1♀ (SCAU, no. 121366), Zhengxing, Jinggu, Yunnan, 23°19′54″N, 100°57′45″E, 1,100–1,300 m a.s.l., 24.vii.2009, ex *tussock* (*L Wang*).

*Diagnosis*. This species is very similar to *S*. (*O*.) *mouig* **sp. nov.** in the shape of phallapodeme anterodistally (Fig. 15C), but can be distinguished by the surstylus with one stout prensisetae dorsally (Fig. 9B) and aedeagus entirely membranous (Fig. 9C).

*Description*. Arista with seven to eight dorsal and four ventral branches. Clypeus dark brown. Mesoscutum mostly yellow, sometimes light brown laterally. Katepisternum brown above, yellow below. Scutellum brownish yellow. Foreleg first tarsus with *ca*. seven fringe-like setae; first to fourth tarsi with *ca*. 11 setae and subapically each with one valgus fringe-like setae on anterior surface (Fig. 3G). Abdominal first and second tergites mostly yellow, dark brown on second tergite posterior margin; third yellow medially, dark brown laterally; fourth to sixth dark brown to black (Fig. 5G). Sternites yellow on first to third, dark brown to black on fourth to sixth. Male terminalia (Fig. 9): Epandrium with *ca*. 22 setae on each posterior portion. Surstylus with two irregular rows of numerous setae medially and a few setae ventrally. Pregonites absent. Aedeagus distally plicated. Aedeagal sheath slender.

*Measurements*. BL = 3.20 mm in holotype (1♀ paratype: 3.00 mm), THL = 1.27 mm (1.27), WL = 2.80 mm (2.73), WW = 1.20 mm (1.27).

*Etymology*. The name denotes “dauntless” in Tibetan.

***Stegana* (*Oxyphortica*) *crypta* Wang & Chen, sp. nov.**

urn:lsid:zoobank.org:act:2CF4A9E7-38C9-47ED-97F4-7CFB849612C2

(Figs. 3H, 5H, 10)

*Type specimen. Holotype*, ♂ (SCAU, no. 111305), CHINA: Dashuigou, Lüchun, Yunnan, 22°57′19″N, 101°57′04″E, 1,310 m a.s.l., 2.viii.2016, ex *tussock* (*H. Chen*).

*Diagnosis*. This species differs from other species in having the surstylus very narrow, with two small prensisetae dorsally (Fig. 10C); aedeagus with numerous serrated processes on lateral margins (Figs. 10C, 10D).

*Description*. Arista with six dorsal and four ventral branches. Clypeus brown. Mesoscutum yellow, slightly brownish laterally and posteriorly. Scutellum brownish yellow. Katepisternum entirely yellow. Foretarsi second to fourth each with one small fringe-like seta on anterior surface (Fig. 3H). Abdominal tergites all yellow medially, brown laterally (Fig. 5H); sternites yellow. Male terminalia (Fig. 10): Epandrium with *ca*. 22 setae on posterior portion per side. Surstylus with two irregular rows of setae medially, and *ca*. six setae ventrally. Pregonites present. Aedeagal sheaths vertical, elongated distolaterally. Aedeagus with a row of setae apically.

*Measurements*. BL = 2.67 mm, THL = 1.10 mm, WL = 2.10 mm, WW = 1.00 mm.

*Etymology*. From the Greek word: *cryptos*, meaning that the fly hides in the mountains.

***Stegana* (*Oxyphortica*) *gelea* Zhang & Chen, sp. nov.**

urn:lsid:zoobank.org:act:91334019-DC7F-4922-9D95-25B8C7CD64C2

(Figs. 3I, 5I, 11)

*Type specimens. Holotype*, ♂ (SCAU, no. 121356), CHINA: Tongmai, Bomi, Xizang, 30°06′10″N, 95°04′48″E, 2,080 m a.s.l., 11.x.2010, ex *tussock* (*J. Gao*). *Paratypes*, CHINA: 3♂, 3♀ (1♂, 1♀ in KIZ, nos 0090653–54; 2♂, 2♀ in SCAU, nos 121357–60), 9.x.2010, same data as holotype (*J. Gao*); 2♂, 2♀ (TMNS), Beibeng, Motuo, Xizang, 29°14′36″N, 95°19′56″E, 1,100 m a.s.l., 27.ix.2010, ex *tussocks* (*Y. Su*, *L Wang*, *L Wu*).

*Diagnosis*. This species is very similar to *S*. (*O*.) *wanhei* **sp. nov.** in the male terminalia structure, but can be distinguished from the latter by the apical processes of aedeagus and aedeagal sheaths are nearly equilong (Figs. 11C, 11D), foreleg first tarsus subapically to apically with three recurvate fringe-like setae on anterior surface (Fig. 3I).

*Description*. Arista with six dorsal and four to five ventral branches. Clypeus black. Mesoscutum brownish yellow. Katepisternum brown anteriorly, yellow posteriorly. Scutellum brown, yellow along margin. Abdominal first tergite yellow; second to fourth tergites dark brown, yellow anteromedially; fifth and sixth tergites black, yellow on anterior margins (Fig. 5I). Sternites entirely yellow. Male terminalia (Fig. 11): Epandrium with *ca*. 30 setae on posterior portion per side. Surstylus with two pointed prensisetae dorsally and two irregular rows of numerous setae medially, elongated and curved ventrally. Pregonites small. Aedeagus with several setae medially, mediodorsally fused with aedeagal sheaths, bifurcated distally. Aedeagal sheaths bifurcated distally.

*Measurements*. BL = 3.34 mm in the holotype (4*♂* and 5♀ paratypes: 3.16–3.56 in ♂, 3.00–4.00 in ♀), THL = 1.64 mm (1.48–1.74, 1.48–1.88), WL = 3.56 mm (2.88–3.44, 2.88–3.80), WW = 1.76 mm (1.32–1.60, 1.28–2.90).

*Etymology*. The name means “lucky place” in Tibetan.

***Stegana* (*Oxyphortica*) *hengduanmontana* Zhang & Chen, sp. nov.**

urn:lsid:zoobank.org:act:C2BA7D62-428B-4FA7-9507-24AC574813E8

(Figs. 3J, 5J, 12)

*Type specimens. Holotype*, ♂ (SCAU, no. 122227), CHINA: Yixiang, Pu′er, Yunnan, 22°44′19″N, 101°07′01″E, 1,420 m a.s.l., 12.xii.2012, ex *tussock* (*J. Gao*). *Paratypes*, CHINA: 1♀ (SCAU, no. 122228), same data as holotype; 3♂ (SCAU, nos 111273–75), 28.iii.2013, same data as holotype; 1♂, 1♀ (SCAU, nos 111719, 20), Mengma, Menglian, Yunnan, 22°12′59″N, 99°22′51″E, 1,060 m a.s.l., 14.iv.2018, ex *tussock* (*L Gong*).

*Diagnosis*. This species differs from other species in having the foreleg first tarsus with *ca*. 12 small, fringe-like setae on anterior surface (Fig. 3J); aedeagus and aedeagal sheaths strongly sclerotized and glabrous (Figs. 12C, 12D).

*Description*. Arista with six dorsal and four ventral branches. Clypeus brown. Mesoscutum and scutellum brownish yellow. Katepisternum brown anteriorly, yellow posteriorly. Abdominal first tergite yellow; second and third tergites yellow medially, brown laterally; fourth to sixth mostly brown, with yellow anteromedially (Fig. 5J). Sternites yellow. Male terminalia (Fig. 12): Epandrium with two irregular rows of *ca*. 20 setae along each posterior margin. Cercus with pubescence. Surstylus with two stout prensisetae dorsally, pubescence medially, and *ca*. five setae ventrally. Pregonites present. Aedeagus apically bifurcated and curved. Aedeagal sheaths anterolaterally elongated, curved and fused with phallapodeme, posteriorly protruded.

*Measurements*. BL = 2.80 mm in holotype (1♀ paratype: 2.60 mm), THL = 1.13 mm (1.13), WL = 2.53 mm (2.47), WW = 1.00 mm (1.07).

*Etymology*. A combination of the type locality + the Latin word *montanus* (= montane), referring to the specimens were collected in Hengduan Mountains.

***Stegana* (*Oxyphortica*) *jinmingi* Zhang & Chen, sp. nov.**

urn:lsid:zoobank.org:act:D744D8E0-9475-474F-971F-8EDE39E9C756

(Figs. 4A, 5K, 13)

*Type specimens. Holotype*, ♂ (SCAU, no. 121251), CHINA: Caiyanghe Forest Park, Pu′er, Yunnan, 27°11′12″N, 100°02′59″E, 900–1,400 m a.s.l., 27.vii.2009, ex *tussock* (*L Wu*). *Paratypes*, CHINA: 1♂, 1♀ (SCAU, nos 121252, 53), same data as holotype; 2♀ (KIZ, nos 0090596, 97), Yixiang, Pu′er, Yunnan, 22°44′19″N, 101°07′01″E, 1,420 m a.s.l., 28.iii.2013, ex *tussock* (*J. Gao*); 2♂, 5♀ (SCAU, nos 121254, 55, 121495, 122706–09), Hesong, Menghai, Yunnan, 21°50′08″N, 100°05′51″E, 1,700–1,900 m a.s.l., 27.iii.2011, 12.v.2012, ex *tussocks* (*J. Gao*, *J. Lu*, *Y. Su*, *L Wang*, *L Wu*, *S. Yan*); 4♂ (SCAU, nos 122710–13), Husa, Longchuan, Yunnan, 24°27′37″N, 97°45′9″E, 1,230–1,340 m a.s.l., 20.viii.2016, ex *tussocks* (*H. Chen*, *L Gong*, *Y. Liu*); 1♂, 6♀ (SCAU, nos 111259–65), Qimaba, Lüchun, Yunnan, 22°49′03″N, 102°17″39″E, 1,280 m a.s.l., 30.x.2016, ex *tussocks* (*H. Chen*); 6♂ (SCAU, nos 111714, 15, 111017–20), CHINA: Botanic Garden, Ruili, Yunnan, 24°01′12″N, 97°51′33″E, 1,170 m a.s.l., 6.xi.2017, ex *tussocks* (*H. Chen*, *L Gong*, *B. Li*).

*Diagnosis*. This species is very similar to *S*. (*O*.) *wanhei* **sp. nov.** in the male terminalia, but can be distinguished from the latter by the apical processes of aedeagal sheaths slightly curved in lateral view (Fig. 13D); foretarsi first and second with two rows of *ca*. 10 long, recurvate fringe-like setae; first to fourth subapically each with one valgus fringe-like seta on anterior surface (Fig. 4A).

*Description*. Arista with six dorsal and four ventral branches. Clypeus brown. Mesoscutum yellow, sometimes light brown laterally. Katepisternum yellow, brown on anterior corner. Scutellum brownish yellow. Abdominal first tergite yellow; second and third tergites yellow medially, dark brown laterally; fourth and fifth mostly dark brown, yellow anteromedially; sixth dark brown (Fig. 5K). Sternites yellowish brown. Male terminalia (Fig. 13): Epandrium with *ca*. 27 setae on posterior portion per side. Surstylus with two pointed prensisetae dorsally and numerous setae ventrally, elongated and slightly curved ventrally. Pregonites absent. Aedeagus with several setae medially, mediodorsally fused with aedeagal sheaths, bifurcated distally. Aedeagal sheaths bifurcated distally.

*Measurements*. BL = 2.50 mm in holotype (1♂ paratype: 2.80 mm), THL = 1.34 mm (1.37), WL = 2.18 mm (2.33), WW = 1. 00 mm (1.13).

*Etymology*. Patronym after the collector Mr. Jinming Lu (SCAU).

***Stegana* (*Oxyphortica*) *mengbalanaxi* Zhang & Chen, sp. nov.**

urn:lsid:zoobank.org:act:35238DA6-11C8-49AC-8BA2-B4511D549F65

(Figs. 4B, 5L, 14)

*Type specimens. Holotype*, ♂ (SCAU, no. 121367), CHINA: Baihualing, Baoshan, Yunnan, 25°46′02″N, 98°43′44″E, 1,400–1,600 m a.s.l., 14.vi.2011, ex *tussock* (*J. Gao*). *Paratypes*, CHINA: 1♀ (SCAU, no. 121368), same data as holotype (*H. Chen*); 1♂, 1♀ (SCAU, nos 124776, 77), 21.vi.2013, same data as holotype (*Y. Wang*); 1♂ (SCAU, no. 124175), Husa, Longchuan, Yunnan, 24°27′37″N, 97°45′9″E, 1,230–1,340 m a.s.l., 20.viii.2016, ex *tree trunk* (*H. Chen*).

*Diagnosis*. This species somewhat resembles *S*. (*O*.) *yangjin* **sp. nov.** in the male terminalia (Figs. 14, 21), but can be distinguished from the latter by the foretarsi first and second with two to three rows of *ca*. 22 long, recurvate fringe-like setae; first to fourth apically each with one short, valgus fringe-like seta on anterior surface (Fig. 4B).

*Description*. Arista with seven to eight dorsal and four to five ventral branches. Clypeus black. Palpus brown. Mesoscutum brownish to brown. Katepisternum dark brown above, yellow below. Scutellum dark brown. Abdominal first tergite yellow; second to sixth tergites mostly dark brown, with yellow patches submedially (Fig. 5L). Sternites brown. Male terminalia (Fig. 14): Epandrium with *ca*. 24 setae on posterior portion per side. Surstylus with two prensisetae dorsally, pubescence and setae submedially and ventrally. Pregonites absent. Aedeagus with several setae medially and numerous minute serrated processes laterally, fused with aedeagal sheaths dorsomedially. Aedeagal sheaths curved posteriorly, acute apically.

*Measurements*. BL = 3.00 mm in holotype (1♂ paratype: 2.33 mm), THL = 1.20 mm (1.00), WL = 2.67 mm (2.00), WW = 1.40 mm (0.93).

*Etymology*. The name means “dreamlike homeland”, from the language of the Dai ethnic group in Xishuangbanna, Yunnan, China.

***Stegana* (*Oxyphortica*) *mouig* Zhang & Chen, sp. nov.**

urn:lsid:zoobank.org:act:7991CFC0-2FCC-426C-AEAE-5CA088F2EB46

(Figs. 4C, 5M, 15)

*Type specimens. Holotype*, ♂ (SCAU, no. 121369), CHINA: Yixiang, Pu′er, Yunnan, 22°44′19″N, 101°07′01″E, 1,420 m a.s.l., 2.x.2011, ex *tussock* (*H. Chen*). *Paratypes*, CHIAN: 1♂, 1♀ (SCAU, nos 111134, 121370), same data as holotype; 11♂, 4♀ (5♂, 2♀ in KIZ, nos 0090598–604; 6♂, 2♀ in SCAU, nos 111135–42), 28–30.iii.2013, 28.v.2015, ex *tussocks and tree trunks*, same data as holotype (*H. Chen*, *J. Gao*); 3♂ (SCAU, nos 122931, 123019, 20), Wuliangshan, Jingdong, Yunnan, 24°17′27″N, 100°19′48″E, 1,700–2,100 m a.s.l., 1.ix.2014, ex *tussock* (*J. Gao*); 35♂, 25♀ (10♂, 10♀ in KIZ, nos 0090605–24; 15♂, 15♀ in SCAU, nos 111143–69, 111192–201, 111721–23), Mengdong, Cangyuan, Yunnan, 23°100′8″N, 99°13′52″E, 1,320–1,400 m a.s.l., 6.v.2016, 19.iv.2019, ex *tussocks and tree trunks* (*H. Chen*, *L Gong*, *J Huang*, *Y Lin*, *Y. Liu*, *Y. Wang*, *L Zhu*); 14♂, 2♀ (SCAU, nos 111170–85), Dashuigou, Lüchun, Yunnan, 22°57′19″N, 101°57′04″E, 1,310 m a.s.l., 2.viii.2016, ex *tussocks and tree trunks* (*H. Chen*, *L Gong*, *Y. Liu*); 5♂ (SCAU, nos 111186–91), Husa, Longchuan, Yunnan, 24°27′37″N, 97°45′9″E, 1,230–1,340 m a.s.l., 20.viii.2016, ex *tussocks* (*H. Chen*, *L Gong*, *Y. Liu*).

*Diagnosis*. This species differs from *S*. (*O*.) *bawo* **sp. nov.** in having the surstylus with two stout prensisetae dorsally (Fig. 15B); aedeagus round distally, slightly sclerotized medially (Fig. 15C).

*Description*. Arista with four to six dorsal and three to four ventral branches. Clypeus brown. Mesoscutum yellow, with brown longitudinal stripes. Katepisternum yellow. Scutellum brown, yellow along margin. Foretarsi first to fourth with a row of *ca*. 13 long, recurvate fringe-like setae, and subapically each with one short, valgus fringe-like seta on anterior surface (Fig. 4C). Abdominal first tergite yellow; second tergite yellow medially, dark brown laterally; third tergite mostly dark brown, with one pair of yellow patches medially; fourth to sixth dark brown (Fig. 5M). Sternites yellow. Male terminalia (Fig. 15): Epandrium with *ca*. 19 setae on posterior portion per side. Surstylus with numerous setae submedially and ventrally. Aedeagal sheath slender.

*Measurements*. BL = 2.67 mm in holotype (5♂ and 5♀ paratypes: 2.33–3.00 in ♂, 2.27–3.33 in ♀), THL = 1.17 mm (1.00–1.33, 1.00–1.33), WL = 2.67 mm (2.27–2.83, 2.33–3.17), WW = 1.07 mm (0.90–1.13, 1.00–1.27).

*Etymology*. The name means “the Almighty” in the language of the Va ethnic group in Yunnan.

***Stegana* (*Oxyphortica*) *setipes* Wang & Chen, sp. nov.**

urn:lsid:zoobank.org:act:57CE9B92-7CF3-4CD6-89A0-1E1C38CC0E9D

(Figs. 4D, 5N, 16)

*Type specimen. Holotype*, ♂ (SCAU, no. 111304), CHINA: Hesong, Menghai, Yunnan, 21°50′08″N, 100°05′51″E, 1,700–1,900 m a.s.l., 27.iii.2011, ex *tussock* (*J. Lu*).

*Diagnosis*. This species somewhat resembles *S*. (*O*.) *tsauri* **sp. nov.** in the male terminalia, but can be distinguished from the latter by the foretarsi first and second with two rows of *ca*. 30 long, recurvate fringe-like setae on anterior surface (Fig. 4D); abdominal tergites yellow medially, light brown laterally (Fig. 5N).

*Description*. Arista with five dorsal and three ventral branches. Clypeus brown. Mesoscutum and scutellum yellow. Katepisternum brownish above, yellow below. Male terminalia (Fig. 16): Epandrium with *ca*. 25 setae on posterior portion per side. Surstylus with two prensisetae dorsally, and numerous setae submedially and ventrally. Pregonites present. Aedeagus with several setae medially and numerous minute serrated processes laterally, fused with aedeagal sheaths dorsomedially. Aedeagal sheaths curved posteriorly, acute apically.

*Measurements*. BL = 2.33 mm in holotype, THL = 1.00 mm, WL = 2.03 mm, WW = 0.97 mm.

*Etymology*. A combination of the Latin words: *seta* + *pes*, referring to the foretarsi with long, fringe-like setae.

***Stegana* (*Oxyphortica*) *shangrila* Zhang & Chen, sp. nov.**

urn:lsid:zoobank.org:act:3A1E56C9-47B2-481E-86D7-59827175DE19

(Figs. 4E, 5O, 17)

*Type specimens. Holotype*, ♂ (SCAU, no. 121372), CHINA: Hutiaoxia, Xianggelila, Yunnan, 27°11′24″N, 100°02′54″E, 1,700 m a.s.l., 22.viii.2011, ex *tussock* (*H. Chen*, *J. Gao*). *Paratypes*, CHINA: 2♂, 3♀ (1♂, 1♀ in KIZ, 0090655, 56; 1♂, 2♀ in SCAU, nos 121373–75), same data as holotype.

*Diagnosis*. Foretarsi first to third with a row of *ca*. 10 long, slightly valgus fringe-like setae on anterior surface (Fig. 4E). Aedeagus distally bifurcated, with numerous long and short setae (Figs. 17C, 17D); aedeagal sheaths distally triangular, strongly protruded posteriorly, plicated laterally, anterolaterally elongated, curved and fused with phallapodeme (Figs. 17C, 17D).

*Description*. Arista with seven to eight dorsal and three to five ventral branches. Clypeus brown. Mesoscutum, katepisternum and scutellum yellow. Abdominal first to fourth tergites yellow medially, brown to dark brown laterally; fifth and sixth dark brown, with yellow anteromedially (Fig. 5O). Sternites entirely yellow. Male terminalia (Fig. 17): Epandrium with *ca*. 19 setae on posterior portion per side. Cercus with pubescence dorsally. Surstylus with one stout prensiseta dorsally. Pregonites absent. Aedeagal sheaths elongated distolaterally, fused with phallapodeme.

*Measurements*. BL = 2.67 mm in holotype (2♂ and 3♀ paratypes: 2.53 in ♂, 3.00–3.13 in ♀), THL = 1.07 mm (1.07, 1.20–1.27), WL = 2.20 mm (2.13, 2.53–2.87), WW = 1.07 mm (1.07, 1.20–1.60).

*Etymology*. From the Tibetan lection, meaning an enchanting place.

***Stegana* (*Oxyphortica*) *tsauri* Zhang & Chen, sp. nov.**

urn:lsid:zoobank.org:act:420D7B97-DDCC-4C46-B937-380DEECA2DF5

(Figs. 4F, 5P, 18)

*Type specimens. Holotype*, ♂ (SCAU, no. 122714), CHINA: Dabang, Chiayi, Taiwan, 23°28′47″N, 120°39′11″E, 630–800 m a.s.l., 15.x.2012, ex *tree trunk* (*H. Chen*). *Paratypes*, CHINA: 1♂, 1♀ (SCAU, nos 122715, 16), same data as holotype.

*Diagnosis*. This species differs from *S*. (*O*.) *mengbalanaxi* **sp. nov.** in having the foretarsi first and second with two to three rows of *ca*. 28 long, recurvate fringe-like setae, and first to fourth subapically each with one short, valgus fringe-like seta on anterior surface (Fig. 4F); abdominal first tergite brown; second medially brown, laterally black; third mostly black, brown along anterior margin; fourth to sixth black (Fig. 5P).

*Description*. Arista with five dorsal and three to four ventral branches. Clypeus black. Mesoscutum and scutellum brown. Katepisternum brownish. Abdominal sternites nearly brown. Male terminalia (Fig. 18): Epandrium with *ca*. 29 setae on posterior portion per side. Surstylus with one ventral and two dorsal prensisetae, and numerous setae submedially and ventrally. Pregonites absent. Aedeagus with several setae medially and numerous minute serrated processes laterally, fused with aedeagal sheaths dorsomedially. Aedeagal sheaths curved posteriorly, acute apically.

*Measurements*. BL = 2.73 mm in holotype (1♂ and 1♀ paratypes: 2.47 in ♂, 2.80 in ♀), THL = 1.13 mm (1.07, 1.13), WL = 2.67 mm (2.60, 3.00), WW = 1.07 mm (0.93, 1.13).

*Etymology*. The species is named in honor of Dr. Shun-Chern Tsaur (National Taiwan University) for helping HW Chen′s research in Taiwan.

***Stegana* (*Oxyphortica*) *valleculata* Zhang & Chen, sp. nov.**

urn:lsid:zoobank.org:act:56D44D2D-0311-4A1A-B983-A0374959F113

(Figs. 4G, 5Q, 19)

*Type specimens. Holotype*, ♂ (SCAU, no. 123005), CHINA: Muyiji Park, Ximeng, Yunnan, 22°37′15″N, 99°35′42″E, 1,100–1,200 m a.s.l., 2.iv.2011, ex *tussock* (*J. Lu*). *Paratypes*, CHINA: 3♀ (SCAU, nos 123006–08), same data as holotype; 1♂, 1♀ (SCAU, nos 123009, 10), Yixiang, Pu′er, Yunnan, 22°44′19″N, 101°07′01″E, 1,420 m a.s.l., 12.xii.2012, ex *tussock* (*J. Gao*); 2♀ (SCAU, nos 111724, 25), Muyiji Park, Ximeng, Yunnan, 22°37′15″N, 99°35′42″E, 1,100–1,200 m a.s.l., 16.iv.2018, ex *tree trunk*, (*L Gong*); 1♂ (SCAU, no. 111437), Guanlei, Mengla, Yunnan, 22°06′26″N, 100°45′46″E, 650–870 m a.s.l., 24.iv.2016, ex *tussock* (*J Huang*).

*Diagnosis*. This species resembles *S*. (*O*.) *shangrila* **sp. nov.** in the aedeagus bifurcated, with short setae (Figs. 19C, 19D), but can be distinguished from the latter by the foretarsi first to third with two rows of *ca*. 27 long, slightly curved fringe-like setae on anterior surface (Fig. 4G), and surstylus with two stout prensisetae dorsally (Fig. 19B).

*Description*. Arista with five to six dorsal and three to four ventral branches. Clypeus brown. Mesoscutum and scutellum yellow. Katepisternum yellowish. Abdominal first tergite yellow; second and third tergites yellow medially, brown laterally; fourth and fifth yellow medially and brown along posterior margins and laterally in male, nearly brown to dark in female; sixth brown to dark brown (Fig. 5Q). Sternites yellow. Male terminalia (Fig. 19): Epandrium with *ca*. 19 setae on posterior portion per side. Surstylus with a row of numerous setae submedially and *ca*. seven setae ventrally. Pregonites absent. Aedeagal sheaths distolaterally elongated, curved and fused with phallapodeme.

*Measurements*. BL = 3.07 mm in holotype (1♂ and 4♀ paratypes: 2.97 in ♂, 2.73–3.17 in ♀), THL = 1.07 mm (1.10, 1.07–1.27), WL = 2.73 mm (2.90, 2.53–3.07), WW = 1.06 mm (1.10, 1.06–1.20).

*Etymology*. From the Latin word: *valleculatus*, referring to that the fly hides in the valley.

***Stegana* (*Oxyphortica*) *wanhei* Zhang & Chen, sp. nov.**

urn:lsid:zoobank.org:act:D00C2F60-7938-461C-A234-B48C76A40FB2

(Figs. 4H, 5R, 20)

*Type specimens. Holotype*, ♂ (SCAU, no. 111202), CHINA: Hutiaoxia, Xianggelila, Yunnan, 27°11′24″N, 100°02′54″E, 1,700 m a.s.l., 22.viii.2011, ex *tussock* (*H. Chen*). *Paratypes*, CHINA: 48♂, 28♀ (10♂, 10♀ in KIZ, nos 0090625–44; 38♂, 18♀ in SCAU, nos 111203–58), ex *tussocks and tree trunks*, same data as holotype (*H. Chen*, *J. Gao*); 4♀ (SCAU, nos 121376–79), Menglun, Mengla, Yunnan, 21°59′57″N, 101°02′50″E, 820–890 m a.s.l., 24–26.xii.2003, ex *tree trunks* (*H. Chen*); 2♀ (SCAU, nos 121380, 81), Niuluohe, Jiangcheng, Yunnan, 22°30′N, 101°53′E, 1,000 m a.s.l., 21.iv.2010, ex *tussock* (*L Wang*); 1♂, 1♀ (SCAU, nos 122931, 32), Wuliangshan, Jingdong, Yunnan, 24°17′27″N, 100°19′48″E, 1,700–2,100 m a.s.l., 1.ix.2014, ex *tussock* (*J. Gao*); 2♂, 1♀ (SCAU, nos 122933–35), Xianheping, Anlong, Guizhou, 24°59′36″N, 105°36′14″E, 1,500 m a.s.l., 16.vii.2014, ex *tussock* (*Y Zhang*); 1♀ (SCAU, no. 122936), Wuliangshan, Jingdong, Yunnan, 24°17′27″N, 100°19′48″E, 1,700–2,100 m a.s.l., 16.vii.2009, ex *tussock* (*L Wang*); 1♂, 6♀ (SCAU, nos 111259–65), Qimaba, Lüchun, Yunnan, 22°49′03″N, 102°17″39″E, 1,280 m a.s.l., 30.x.2016, ex *tussock* (*H. Chen*); 4♂, 2♀ (SCAU, nos 111431–36), Nanling, Lancang, Yunnan, 22°47′57″N, 99°55′11″E, 1,800 m a.s.l., 12.viii.2016, ex *tussock* (*H. Chen*, *L Gong*).

*Diagnosis*. This species differs from *S*. (*O*.) *gelea* **sp. nov.** and *S*. (*O*.) *jinmingi* **sp. nov.** in having the apical processes of aedeagal sheaths curved in lateral view (Fig. 20D), abdominal fifth and sixth tergites yellow medially, narrowly black along lateral margin (Fig. 5R), and foretarsi first subapically with two rows of *ca*. 10 recurvate fringe-like setae, second with two short fringe-like setae on anterior surface (Fig. 4H).

*Description*. Arista with seven to nine dorsal and four to five ventral branches. Clypeus brown. Mesoscutum and scutellum brownish yellow. Katepisternum brown anteriorly, yellow posteriorly. Abdominal first tergite yellow; second to fourth tergites yellow medially, black laterally (Fig. 5R). Sternites yellow. Male terminalia (Fig. 20): Epandrium with *ca*. 35 setae on posterior portion per side. Surstylus with two prensisetae dorsally and two irregular rows of numerous setae submedially, acute ventrally. Aedeagus with several setae medially, mediodorsally fused with aedeagal sheaths, bifurcated distally. Aedeagal sheaths bifurcated distally.

*Measurements*. BL = 3.00 mm in holotype (5♂ and 5♀ paratypes: 2.87–3.40 in ♂, 3.53–4.47 in ♀), THL = 1.40 mm (1.40–1.60, 1.33–1.67), WL = 2.67 mm (2.87–3.00, 3.00–3.40), WW = 1.27 mm (1.27–1.47, 1.40–1.60).

*Etymology*. The species is named in honor of Mr. Wanhe Zhang (KIZ), a very capable driver in our multi-sited fieldworks.

***Stegana* (*Oxyphortica*) *yangjin* Zhang & Chen, sp. nov.**

urn:lsid:zoobank.org:act:2316FD6E-5ED4-43EA-A3B3-1F48275BE2F8

(Figs. 4I, 5S, 21)

*Type specimen. Holotype*, ♂ (SCAU, no. 121371), CHINA: Beibeng, Motuo, Xizang, 29°14′36″N, 95°19′56″E, 1,100 m a.s.l., 2.x.2010, ex *tussock* (*J. Gao*).

*Diagnosis*. This species differs from *S*. (*O*.) *mengbalanaxi* **sp. nov.** in having the foretarsi first and second with two rows of *ca*. 12 long, slightly recurvate fringe-like setae on anterior surface (Fig. 4I), and abdominal tergites brown on first to third, dark brown on fourth to sixth (Fig. 5S).

*Description*. Arista with five dorsal and four ventral branches. Clypeus brownish yellow. Mesoscutum and scutellum yellow. Katepisternum yellow, slightly brown anteriorly. Abdominal sternites yellow. Male terminalia (Fig. 21): Epandrium with *ca*. 19 setae on posterior portion per side. Surstylus with two prensisetae dorsally, pubescence medially and setae submedially and ventrally. Pregonites absent. Aedeagus with several setae medially and numerous minute serrated processes laterally, fused with aedeagal sheaths dorsomedially. Aedeagal sheaths curved posteriorly, acute apically.

*Measurements*. BL = 2.84 mm in holotype, THL = 1.42 mm, WL = 2.34 mm, WW = 0.98 mm.

*Etymology*. A girl name from the Tibetan, meaning “her singing as though heavenly sounds in the full-moonlit starry sky”.

***Stegana* (*Oxyphortica*) *hypophaia* Zhang & Chen, sp. nov.**

urn:lsid:zoobank.org:act:072AD3E7-F24B-4605-83EF-1CF9E9FA41D8

(Figs. 4J; 5T; 22)

*Type specimens. Holotype*, ♂ (SCAU, no. 123021), CHINA: Xinling, Badong, Hubei, 31°02′24″N, 110°20′48″, 650 m a.s.l., 7.x.2014, ex *tussock* (*J. Gao*). *Paratypes*, CHINA: 1♂, 3♀ (SCAU, nos 123022–24, 111266), same data as holotype.

*Diagnosis*. This species somewhat resembles *S*. (*O*.) *crypta* **sp. nov.** in some characters of the male terminalia (Figs 10, 22), but can be distinguished by the foretarsi first to fourth subapically each with one short, valgus fringe-like seta on anterior surface (Fig. 4J), aedeagal sheaths anterolaterally fused to phallapodeme, and aedeagus distally oblong and plicated (Figs. 22C, 22D).

*Description*. Arista with six to eight dorsal and four to five ventral branches. Clypeus black. Mesoscutum yellow anteriorly, brownish yellow posteriorly, with three thin, brownish, obscure stripes medially and sublaterally. Katepisternum yellow. Scutellum brown. Abdominal first tergite yellow; second to sixth tergites yellow medially, brown to dark brown laterally and posteriorly (Fig. 5T). Sternites yellow. Male terminalia (Fig. 22): Epandrium with *ca*. 20 setae on posterior portion per side. Cercus with sparse pubescence. Surstylus with two stout prensisetae dorsally, numerous setae medially and a few setae ventrally. Pregonites present.

*Measurements*. BL = 3.17 mm in holotype (1♂ and 2♀ paratypes: 2.58 in ♂, 2.27–3.13 in ♀), THL = 1.20 mm (1.17, 0.87–1.25), WL = 2.23 mm (2.06, 1.90–2.37), WW =1.13 mm (0.93, 0.97–1.00).

*Etymology*. A combination of the Greek words: *hypos* (=under) and *phaios* (gray), referring to the gray wing.

## Discussion

In contrast to the claim that all supraspecific units should be monophyletic (Komarek & Beutel, 2006), the phylogenetic analyses conducted herein recovered the paraphyly of the subgenus *Oxyphortica* (Fig. 1), as described by Li et al. (2013). Within this context, the *nigripennis* and *convergens* species groups were defined as monophyletic in both concatenated data and each gene analyses (Figs 1, S1–S5), whereas Clades I and IV were not in the *COI* analysis (Figs S2, S3). Therefore, a combined dataset of mitochondrial and nuclear markers may help to obtain a reliable phylogenetic framework and plausibly reflect the subdivision of groups within this subgenus. Moreover, this study highlights the existence of high levels of cryptic diversity in the subgenus *Oxyphortica*, which is concordant with the ever-growing number of species. The diversity of this subgenus is highly concentrated around Southwest China, highlighting the importance of climatic and topographical temporal and spatial differences for species diversification.

Our results indicate that genetic divergence has not been accompanied by appreciable morphological changes. This occurs, for example, in the allopatric species pairs in Clade I, *S*. (*O*.) *chuanjiangi* Zhang & Chen, 2017 (southwestern Yunnan) and *S*. (*O*.) *dawa* Zhang & Chen, 2017 (Beibeng, Motuo in Xizang), which are distinguished by the aedeagus that is wider than it is long and not slightly bifid apically (Figs. 8D, 10D in Wang et al., 2017). This situation contrasts with the striking diversification in genitalia that occurs in other allopatric drosophilids (*e.g.*, the sibling species *Amiota cuii* Chen & Toda, 2001 and *A. nozawai* Chen & Watabe, 2005, *Pseudostegana bifasciata* Chen & Wang, 2005 and *Ps. acutifoliolata* Li, Gao & Chen, 2010), suggesting recent divergence, with insufficient time to accumulate substantial morphological differentiation (Struck et al., 2018; Fišer, Robinson & Malard, 2018). In fact, it is generally assumed that, in the early stages of speciation, natural selection primarily acts on traits that are closely associated with survival, such as physiological, immunological, or behavioural traits, rather than on morphology (Struck et al., 2018), particularly in extreme environments (Xu & Che, 2019).

Among the newly recognised species, significantly similar male genitalia structures are shared by the species of Clades IV-A, -B and -C (Fig. 1). In Clade IV-A, for example, *S*. (*O*.) *amphigya* **sp. nov.** and *S*. (*O*.) *armillata* **sp. nov.** are distinguished by the second to fourth subapical foretarsi, each having one small, suberect, fringe-like seta on the anterior surface (Figs. 3D, 3E) and a pattern of abdominal stripes (Figs. 5D, 5E). A similar situation occurs among the species of Clades IV-B and -C. Geographically, *S*. (*O*.) *amphigya* **sp. nov.** and *S*. (*O*.) *armillata* **sp. nov.** are allopatric species separated by <300 km (Mengdong, Cangyuan to Dashuigou, Lüchun in Yunnan). Different river systems and the separation of mountains may have led to the emergence of the new species.

Nevertheless, the abdominal stripe sometimes varies within the same species. This occurs, for example, in *S*. (*O*.) *acutipenis* Xu, Gao & Chen, 2007, *S*. (*O*.) *setifrons* Sidorenko, 1997 and *S*. (*O*.) *zhulinae* Wang & Chen, 2018, which possess two or three abdominal stripe patterns (Figs. 5A, 5B in Wang et al., 2017; Figs. 4A–4C, 4F–4H in Huang et al., 2018). These variable abdominal stripe patterns easily lead to confusion in species identification (*e.g. S*. (*O*.) *xiaoyangae* Zhang & Chen, 2018 is similar to *S*. (*O*.) *setifrons* in terms of the male genitalia structure (Figs 5, 7 in Huang et al., 2018) and one pattern of abdominal stripes (Figs. 4B, 4E in Huang et al., 2018), and share one pattern of abdominal stripes with *S*. (*O*.) *zhulinae* (Fig. 4H in Huang et al., 2018)). However, *S*. (*O*.) *acutipenis* occurs only in Mengla, Yunan, whereas *S*. (*O*.) *zhulinae* spans about 300 km in the north–south direction (Xincheng, Yingjiang to Nanling, Lancang in Yunnan). These results further illustrate the rich species diversity of *Oxyphortica* that may occur even in narrow geographical areas.

Accurate species identification is a primary prerequisite for biological research. However, incongruence among gene trees and substitution patterns often occurs. The ABGD analyses suggested two MOTUs for *S*. (*O*.) *dainuo* by *COI* and one MOTU each for *S*. (*O*.) *dainuo* + *S*. (*O*.) *laohlie* and *S*. (*O*.) *aotsukai* + *S*. (*O*.) *nigripennis* by *ND2*. These discordances probably stem from different rates and patterns of molecular evolution. Nevertheless, the result of the BP&P analysis corroborated the species definition based on the morphological characteristics, suggesting the superiority of the concatenated genes in species identification.

In this study, all *Oxyphortica* species inhabit the Oriental Region, except three species, among which, *S*. (*O*.) *nigripennis* and *S*. (*O*.) *dendrobium* extend northward to the southern part of the Palaearctic Region (Kyushu, Japan) and *S*. (*O*.) *convergens* extends southward to the northern part of the Australian Region (New Guinea). These species are especially abundant in the high-altitude mountain valleys of Southwest China, which harbours 67.3% of extant *Oxyphortica* species (including new species), among which 83.8% are endemic. This high species diversity and rich endemism are largely attributed to the complex climate and the north–south longitudinal mountain and canyon landforms in the Hengduan Mountains, known as the ‘folded zone’. Positioned in the northern part of the Indo-Chinese Peninsula and in the eastern border of the Qinghai–Tibet Plateau, Yunnan is greatly influenced by the tropical and East Asian monsoons and by atmospheric circulation formed by the Himalayan and Qinghai–Tibetan climate. This creates great climatic diversity, including a tropical zone (river valley), a subtropical zone (mid-lying mountains), a temperate zone (sub-high mountains), a frigid zone and an alpine desert zone (everlasting snow zone at the highest point of the mountains) (Ji et al., 1999). The north–south longitudinal mountains facilitate the southward movement of northern species, whereas the longitudinal river valleys at low elevations stimulate the northward diffusion of southern species. Together, these conditions indicate that the Hengduan Mountains are a centre of species aggregation and differentiation.

Previous dating studies have shown that the origin of the subgenus *Oxyphortica* dates back to the late Oligocene to early Miocene (Li et al., 2013). The Miocene and subsequent Pliocene are known for rapid speciation and adaptive radiation of different taxa in Southwest China (Wen et al., 2014; Favre et al., 2015; Päckert et al., 2015; Lu et al., 2018; Ding et al., 2020), which resulted from the extensive uplift of both the Qinghai–Tibet Plateau and Hengduan Mountains (Wang et al., 2012). This rapid orogenic movement may have provided numerous ecological opportunities for the increase of parapatric speciation *via* niche differentiation without competitors (Moritz et al., 2000; Gavrilets & Vose, 2005; Favé et al., 2015; Hazzi et al., 2018). The high endemism and the short internode divergence suggest that *Oxyphortica* may have experienced rapid adaptive radiation in Southwest China, where 16 new species were recorded. This study underlines the huge biodiversity of this region and how poorly it is known.

## Supplemental Information

10.7717/peerj.12347/supp-1 Supplemental Information 1 Phylogenetic tree constructed from the maximum likelihood analysis based on the concatenated dataset. Numbers around the nodes indicate the ultrafast bootstrap (UFBP) values. Abbreviations: *L.*, genus *Leucophenga*; *Pa.*, genus *Parastegana*; *Ps.*, genus *Pseudostegana*; *S*., genus *Stegana*; s.s., *sensu* stricto; *St*., subgenus *Steganina*; *O*., subgenus *Oxyphortica*; *Or*., subgenus *Orthostegana*.

10.7717/peerj.12347/supp-2 Supplemental Information 2 Phylogenetic tree constructed from the Bayesian analysis based on the *COI* sequences. Numbers around the nodes indicate the Bayesian posterior probability (PP) values. Abbreviations: *L.*, genus *Leucophenga*; *Pa.*, genus *Parastegana*; *Ps.*, genus *Pseudostegana*; *S*., genus *Stegana*; s.s., *sensu* stricto; *St*., subgenus *Steganina*; *O*., subgenus *Oxyphortica*; *Or*., subgenus *Orthostegana*.

10.7717/peerj.12347/supp-3 Supplemental Information 3 Phylogenetic tree constructed from the maximum likelihood analysis based on the *COI* sequences. Numbers around the nodes indicate the ultrafast bootstrap (UFBP) values. Abbreviations: *L.*, genus *Leucophenga*; *Pa.*, genus *Parastegana*; *Ps.*, genus *Pseudostegana*; *S*., genus *Stegana*; s.s., *sensu* stricto; *St*., subgenus *Steganina*; *O*., subgenus *Oxyphortica*; *Or*., subgenus *Orthostegana*.

10.7717/peerj.12347/supp-4 Supplemental Information 4 Phylogenetic tree constructed from the Bayesian analysis based on the *ND2* sequences. Numbers around the nodes indicate the Bayesian posterior probability (PP) values. Abbreviations: *L.*, genus *Leucophenga*; *Pa.*, genus *Parastegana*; *Ps.*, genus *Pseudostegana*; *S*., genus *Stegana*; s.s., *sensu* stricto; *St*., subgenus *Steganina*; *O*., subgenus *Oxyphortica*; *Or*., subgenus *Orthostegana*.

10.7717/peerj.12347/supp-5 Supplemental Information 5 Phylogenetic tree constructed from the maximum likelihood analysis based on the *ND2* sequences. Numbers around the nodes indicate the ultrafast bootstrap (UFBP) values. Abbreviations: *L.*, genus *Leucophenga*; *Pa.*, genus *Parastegana*; *Ps.*, genus *Pseudostegana*; *S*., genus *Stegana*; s.s., *sensu* stricto; *St*., subgenus *Steganina*; *O*., subgenus *Oxyphortica*; *Or*., subgenus *Orthostegana*.

10.7717/peerj.12347/supp-6 Supplemental Information 6 The Bayesian Phylogenetics and Phylogeography (BP&P) analysis result based on the *COI* and *ND2* data set.

10.7717/peerj.12347/supp-7 Supplemental Information 7 Summary of genetic distances of *COI* gene.

10.7717/peerj.12347/supp-8 Supplemental Information 8 Summary of genetic distances of *ND2* gene.

## Additional Information and Declarations

### Data Availability

The following information was supplied regarding data availability: The raw data is available at NCBI GenBank: MH235736 to MH235747, MH235749 to MH235755, MH235759 to MH235791, MH372081 to MH372086, MH372103 to MH372184, MH372195 to MH372246, MH373118, MH373119, MH373186, MH373187, MH373224 Specimen information and the accession numbers are listed in Table 1.

### New Species Registration

The following information was supplied regarding the registration of a newly described species: Publication LSID: urn:lsid:zoobank.org:pub:1D19FB6F-DBF2-4982-9C9A-D8FBFC1F3F1E Species name: *Stegana (Oxyphortica) amphigya* Wang & Chen sp. nov. urn:lsid:zoobank.org:act:E6BE14A6-5E2B-4DAD-87D6-2819DFAE5202 Species name: *Stegana (Oxyphortica) armillata* Wang & Chen sp. nov. urn:lsid:zoobank.org:act:699B1E1E-35F2-4772-A36E-6BE7277C4575 Species name: *Stegana (Oxyphortica) ashima* Zhang & Chen sp. nov. urn:lsid:zoobank.org:act:5F7EECB0-12D6-419F-90BB-697D9BF2C455 Species name: *Stegana (Oxyphortica) bawo* Zhang & Chen sp. nov. urn:lsid:zoobank.org:act:7EF83FD7-92D3-4831-BBA6-3ACDF305E9D0 Species name: *Stegana (Oxyphortica) crypta* Wang & Chen sp. nov. urn:lsid:zoobank.org:act:2CF4A9E7-38C9-47ED-97F4-7CFB849612C2 Species name: *Stegana (Oxyphortica) gelea* Zhang & Chen sp. nov. urn:lsid:zoobank.org:act:91334019-DC7F-4922-9D95-25B8C7CD64C2 Species name: *Stegana (Oxyphortica) hengduanmontana* Zhang & Chen sp. nov. urn:lsid:zoobank.org:act:C2BA7D62-428B-4FA7-9507-24AC574813E8 Species name: *Stegana (Oxyphortica) jinmingi* Zhang & Chen sp. nov. urn:lsid:zoobank.org:act:D744D8E0-9475-474F-971F-8EDE39E9C756 Species name: *Stegana (Oxyphortica) mengbalanaxi* Zhang & Chen sp. nov. urn:lsid:zoobank.org:act:35238DA6-11C8-49AC-8BA2-B4511D549F65 Species name: *Stegana (Oxyphortica) mouig* Zhang & Chen sp. nov. urn:lsid:zoobank.org:act:7991CFC0-2FCC-426C-AEAE-5CA088F2EB46 Species name: *Stegana (Oxyphortica) setipes* Wang & Chen sp. nov. urn:lsid:zoobank.org:act:57CE9B92-7CF3-4CD6-89A0-1E1C38CC0E9D Species name: *Stegana (Oxyphortica) shangrila* Zhang & Chen sp. nov. urn:lsid:zoobank.org:act:3A1E56C9-47B2-481E-86D7-59827175DE19 Species name: *Stegana (Oxyphortica) tsauri* Zhang & Chen sp. nov. urn:lsid:zoobank.org:act:420D7B97-DDCC-4C46-B937-380DEECA2DF5 Species name: *Stegana (Oxyphortica) valleculata* Zhang & Chen sp. nov. urn:lsid:zoobank.org:act:56D44D2D-0311-4A1A-B983-A0374959F113 Species name: *Stegana (Oxyphortica) wanhei* Zhang & Chen sp. nov. urn:lsid:zoobank.org:act:D00C2F60-7938-461C-A234-B48C76A40FB2 Species name: *Stegana (Oxyphortica) yangjin* Zhang & Chen sp. nov. urn:lsid:zoobank.org:act:2316FD6E-5ED4-43EA-A3B3-1F48275BE2F8 Species name: *Stegana (Oxyphortica) hypophaia* Zhang & Chen sp. nov. urn:lsid:zoobank.org:act:072AD3E7-F24B-4605-83EF-1CF9E9FA41D8.
