# Supplementary figures and images for: Cryptic diversity in the subgenus Oxyphortica (Diptera, Drosophilidae, Stegana)
Source: PeerJ. 2021 Oct 29;9:e12347. doi: 10.7717/peerj.12347 (PMC8559608; doi:10.7717/peerj.12347)

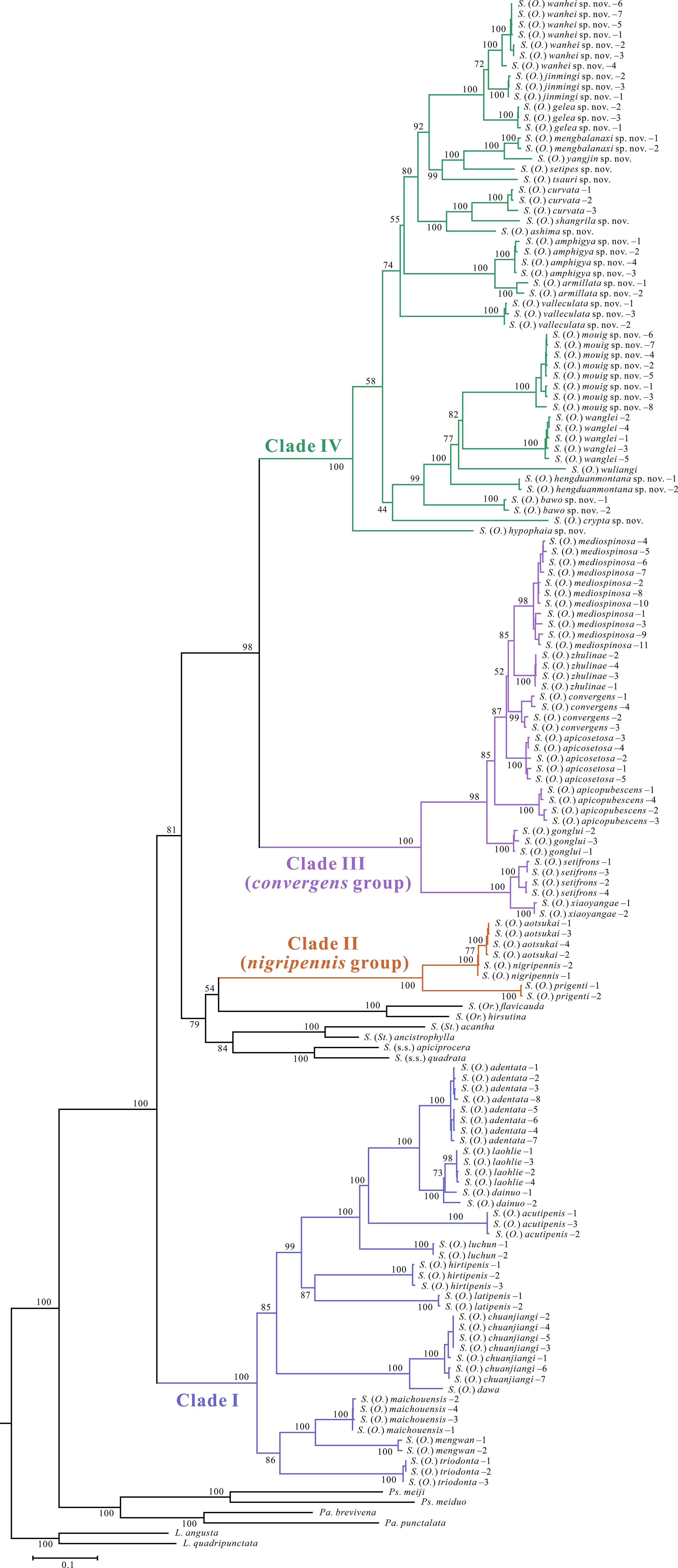

Supplement: Supplemental Information 1 — Numbers around the nodes indicate the ultrafast bootstrap (UFBP) values. Abbreviations: L., genus Leucophenga; Pa., genus Parastegana; Ps., genus Pseudostegana; S., genus Stegana; s.s., sensu stricto; St., subgenus Steganina; O., subgenus Oxyphortica; Or., subgenus Orthostegana. [file peerj-09-12347-s001.png]

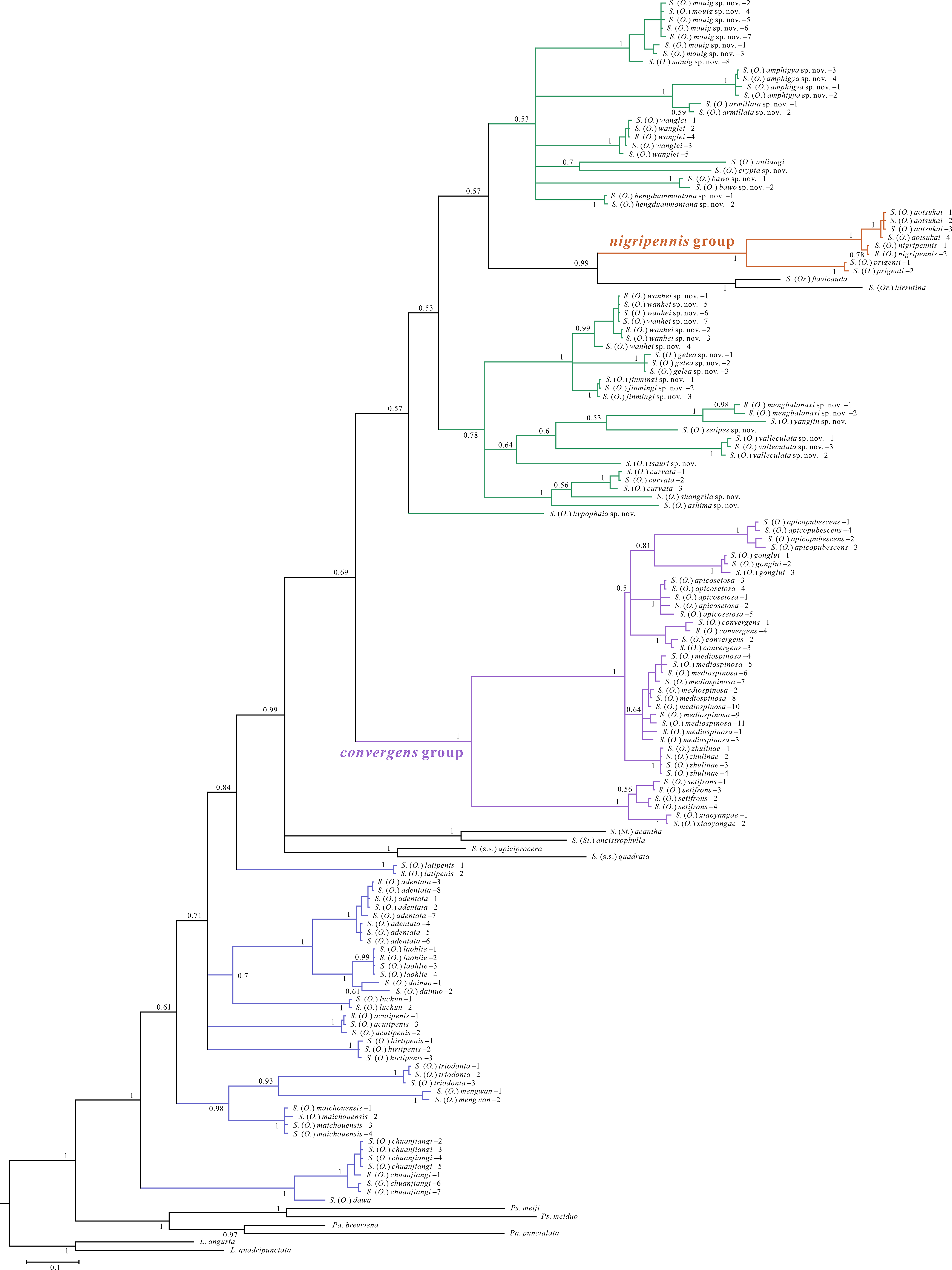

Supplement: Supplemental Information 2 — Numbers around the nodes indicate the Bayesian posterior probability (PP) values. Abbreviations: L., genus Leucophenga; Pa., genus Parastegana; Ps., genus Pseudostegana; S., genus Stegana; s.s., sensu stricto; St., subgenus Steganina; O., subgenus Oxyphortica; Or., subgenus Orthostegana. [file peerj-09-12347-s002.png]

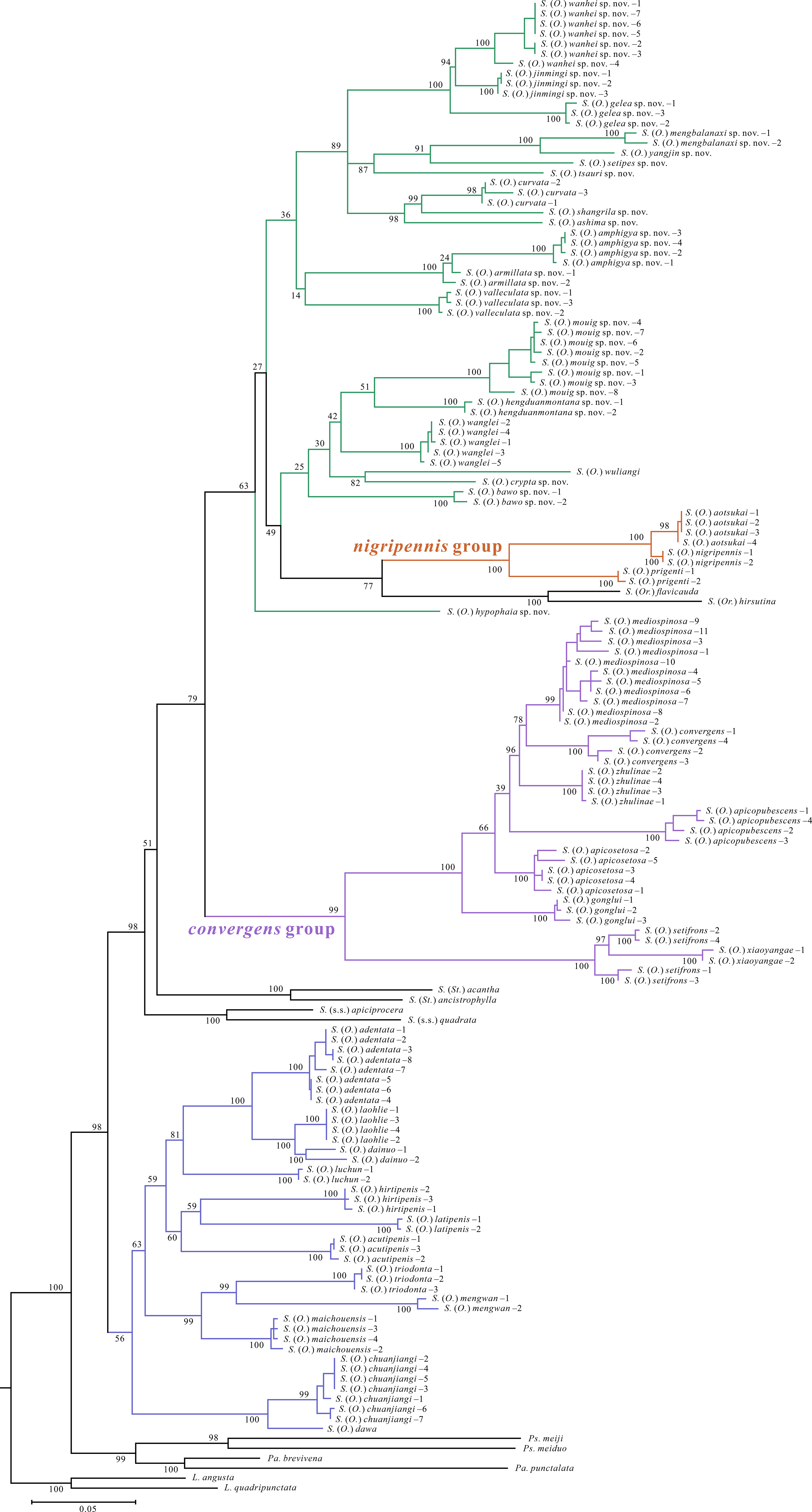

Supplement: Supplemental Information 3 — Numbers around the nodes indicate the ultrafast bootstrap (UFBP) values. Abbreviations: L., genus Leucophenga; Pa., genus Parastegana; Ps., genus Pseudostegana; S., genus Stegana; s.s., sensu stricto; St., subgenus Steganina; O., subgenus Oxyphortica; Or., subgenus Orthostegana. [file peerj-09-12347-s003.png]

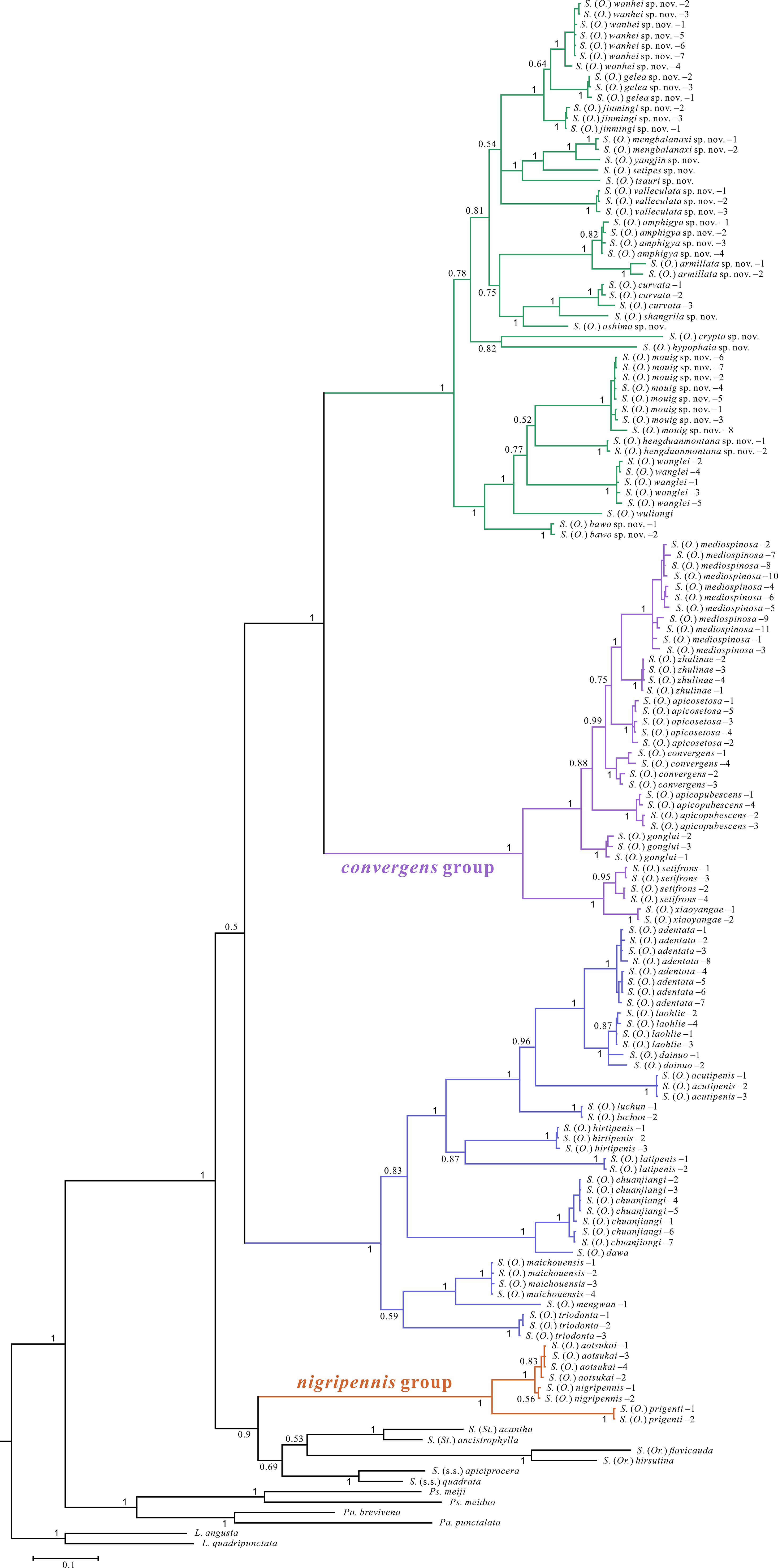

Supplement: Supplemental Information 4 — Numbers around the nodes indicate the Bayesian posterior probability (PP) values. Abbreviations: L., genus Leucophenga; Pa., genus Parastegana; Ps., genus Pseudostegana; S., genus Stegana; s.s., sensu stricto; St., subgenus Steganina; O., subgenus Oxyphortica; Or., subgenus Orthostegana. [file peerj-09-12347-s004.png]

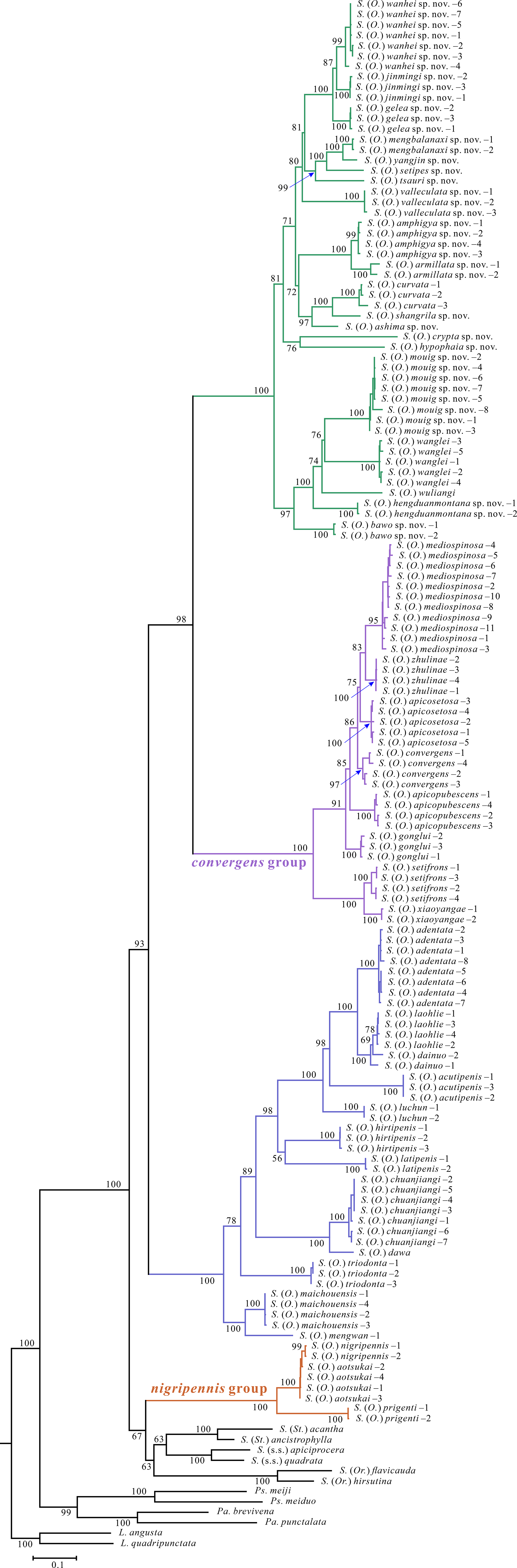

Supplement: Supplemental Information 5 — Numbers around the nodes indicate the ultrafast bootstrap (UFBP) values. Abbreviations: L., genus Leucophenga; Pa., genus Parastegana; Ps., genus Pseudostegana; S., genus Stegana; s.s., sensu stricto; St., subgenus Steganina; O., subgenus Oxyphortica; Or., subgenus Orthostegana. [file peerj-09-12347-s005.png]
