# Supplementary material for: Cryptic diversity in the subgenus Oxyphortica (Diptera, Drosophilidae, Stegana)
Source: PeerJ. 2021 Oct 29;9:e12347. doi: 10.7717/peerj.12347 (PMC8559608; doi:10.7717/peerj.12347)
Supplement: Supplemental Information 6 [file peerj-09-12347-s006.docx]

Table S1. The Bayesian Phylogenetics and Phylogeography (BP&P) analysis result based on the *COI* and *ND2* data set.

| Taxa | Taxon ID | pp1 | pp2 | pp3 | pp4 |
| --- | --- | --- | --- | --- | --- |
| Taxon 1 | *S.* (*O.*) *apicopubescens* (1–4) | 1.00 | 1.00 | 1.00 | 1.00 |
| Taxon 2 | *S.* (*O.*) *apicosetosa* (1–5) | 1.00 | 1.00 | 1.00 | 1.00 |
| Taxon 3 | *S.* (*O.*) *convergens* (1–4) | 1.00 | 1.00 | 1.00 | 1.00 |
| Taxon 4 | *S.* (*O.*) *gonglui* (1–3) | 1.00 | 1.00 | 1.00 | 1.00 |
| Taxon 5 | *S.* (*O.*) *mediospinosa* (1–11) | 1.00 | 1.00 | 1.00 | 1.00 |
| Taxon 6 | *S.* (*O.*) *setifrons* (1–4) | 1.00 | 1.00 | 1.00 | 1.00 |
| Taxon 7 | *S.* (*O.*) *xiaoyangae* (1–2) | 1.00 | 1.00 | 1.00 | 1.00 |
| Taxon 8 | *S.* (*O.*) *zhulinae* (1–4) | 1.00 | 1.00 | 1.00 | 1.00 |
| Taxon 9 | *S.* (*O.*) *aotsukai* (1–4) | 0.86 | 1.00 | 0.87 | 1.00 |
| Taxon 10 | *S.* (*O.*) *nigripennis* (1–2) | 0.86 | 1.00 | 0.87 | 1.00 |
| Taxon 11 | *S.* (*O.*) *prigenti* (1–2) | 1.00 | 1.00 | 1.00 | 1.00 |
| Taxon 12 | *S.* (*O.*) *acutipenis* (1–3) | 1.00 | 1.00 | 1.00 | 1.00 |
| Taxon 13 | *S.* (*O.*) *adentata* (1–8) | 1.00 | 1.00 | 1.00 | 1.00 |
| Taxon 14 | *S.* (*O.*) *chuanjiangi* (1–7) | 1.00 | 1.00 | 1.00 | 0.95 |
| Taxon 15 | *S.* (*O.*) *curvata* (1–3) | 0.99 | 1.00 | 0.99 | 1.00 |
| Taxon 16 | *S.* (*O.*) *dainuo* (1–2) | 1.00 | 1.00 | 1.00 | 1.00 |
| Taxon 17 | *S.* (*O.*) *dawa* | 1.00 | 1.00 | 0.99 | 0.95 |
| Taxon 18 | *S.* (*O.*) *hirtipenis* (1–3) | 1.00 | 1.00 | 1.00 | 1.00 |
| Taxon 19 | *S.* (*O.*) *laohlie* (1–4) | 1.00 | 1.00 | 1.00 | 1.00 |
| Taxon 20 | *S.* (*O.*) *latipenis* (1–2) | 1.00 | 1.00 | 1.00 | 1.00 |
| Taxon 21 | *S.* (*O.*) *luchun* (1–2) | 1.00 | 1.00 | 1.00 | 1.00 |
| Taxon 22 | *S.* (*O.*) *maichouensis* (1–4) | 1.00 | 1.00 | 1.00 | 0.95 |
| Taxon 23 | *S.* (*O.*) *mengwan* (1–2) | 1.00 | 1.00 | 0.99 | 0.95 |
| Taxon 24 | *S.* (*O.*) *triodonta* (1–3) | 1.00 | 1.00 | 1.00 | 0.95 |
| Taxon 25 | *S.* (*O.*) *wanglei* (1–5) | 1.00 | 1.00 | 1.00 | 1.00 |
| Taxon 26 | *S.* (*O.*) *wuliangi* | 0.91 | 1.00 | 0.93 | 1.00 |
| Taxon 27 | *S.* (*O.*) *amphigya* sp. nov. (1–4) | 1.00 | 1.00 | 1.00 | 1.00 |
| Taxon 28 | *S.* (*O.*) *armillata* sp. nov. (1–2) | 1.00 | 1.00 | 1.00 | 1.00 |
| Taxon 29 | *S.* (*O.*) *ashima* sp. nov. | 0.96 | 1.00 | 0.96 | 1.00 |
| Taxon 30 | *S.* (*O.*) *bawo* sp. nov. | 0.99 | 1.00 | 0.99 | 1.00 |
| Taxon 31 | *S.* (*O.*) *crypta* sp. nov. | 0.91 | 1.00 | 0.93 | 1.00 |
| Taxon 32 | *S.* (*O.) gelea* sp. nov. (1–3) | 1.00 | 1.00 | 1.00 | 1.00 |
| Taxon 33 | *S.* (*O.*) *hengduanmontana* sp. nov. (1–2) | 1.00 | 1.00 | 1.00 | 1.00 |
| Taxon 34 | *S.* (*O.*) *hypophaia* sp. nov. | 1.00 | 1.00 | 0.97 | 1.00 |
| Taxon 35 | *S.* (*O.*) *jinmingi* sp. nov. (1–3) | 1.00 | 1.00 | 1.00 | 1.00 |
| Taxon 36 | *S.* (*O.*) *mengbalanaxi* sp. nov. (1–2) | 0.93 | 1.00 | 0.95 | 1.00 |
| Taxon 37 | *S.* (*O.*) *mouig* sp. nov. (1–8) | 1.00 | 1.00 | 1.00 | 1.00 |
| Taxon 38 | *S.* (*O.*) *setipes* sp. nov. | 0.97 | 1.00 | 0.98 | 1.00 |
| Taxon 39 | *S.* (*O.*) *shangrila* sp. nov. | 0.96 | 1.00 | 0.96 | 1.00 |
| Taxon 40 | *S.* (*O.*) *tsauri* sp. nov. | 0.98 | 1.00 | 0.98 | 1.00 |
| Taxon 41 | *S.* (*O.*) *valleculata* sp. nov. (1–3) | 1.00 | 1.00 | 1.00 | 1.00 |
| Taxon 42 | *S.* (*O.*) *wanhei* sp. nov. (1–7) | 1.00 | 1.00 | 1.00 | 1.00 |
| Taxon 43 | *S.* (*O.*) *yangjin* sp. nov. | 0.93 | 1.00 | 0.95 | 1.00 |

pp1, pp2, pp3, pp4, speciation posterior probabilities under three prior settings: (1) large ancestral population sizes and deep divergences: θ ~ G (1, 10) and τ_0_ ~ G (1, 10); (2) small ancestral population sizes and shallow divergences: θ ~ G (2, 2000) and τ_0_ ~ G (2, 2000); (3) large ancestral population sizes and shallow divergences: θ ~ G (1, 10) and τ_0_ ~ G (2, 2000); (4) small ancestral population sizes and deep divergences: θ ~ G (2, 2000) and τ_0_ ~ G (1, 10).
