# Supplementary material for: Cryptic diversity in the subgenus Oxyphortica (Diptera, Drosophilidae, Stegana)
Source: PeerJ. 2021 Oct 29;9:e12347. doi: 10.7717/peerj.12347 (PMC8559608; doi:10.7717/peerj.12347)
Supplement: Supplemental Information 7 [file peerj-09-12347-s007.docx]

Table S2. Summary of genetic distances of *COI* gene.

| Species | N | Min.intra./Max.intra./Mean intra. ±SD | Min.inter./Max.inter./Mean inter. ±SD |
| --- | --- | --- | --- |
| *S. acutipenis* | 3 | 0.000/0.006/0.004±0.003 | 0.092/0.182/0.140±0.025 |
| *S. adentata* | 8 | 0.000/0.013/0.008±0.005 | 0.050/0.167/0.129±0.025 |
| *S. aotsukai* | 4 | 0.000/0.000/0.000±0.000 | 0.015/0.186/0.148±0.023 |
| *S. apicopubescens* | 4 | 0.004/0.017/0.013±0.005 | 0.079/0.190/0.152±0.033 |
| *S. apicosetosa* | 5 | 0.000/0.021/0.013±0.006 | 0.050/0.173/0.130±0.034 |
| *S. chuanjiangi* | 7 | 0.000/0.010/0.005±0.004 | 0.036/0.188/0.137±0.021 |
| *S. convergens* | 4 | 0.008/0.035/0.025±0.011 | 0.040/0.188/0.138±0.039 |
| *S. curvata* | 3 | 0.002/0.015/0.010/0.006 | 0.071/0.173/0.134±0.024 |
| *S. dainuo* | 2 | 0.029 | 0.031/0.171/0.128±0.031 |
| *S. dawa* | 1 | NA/NA/NA | 0.036/0.182/0.136±0.031 |
| *S. gonglui* | 3 | 0.002/0.012/0.008±0.004 | 0.061/0.177/0.136±0.033 |
| *S. hirtipenis* | 3 | 0.000/0.004/0.003±0.002 | 0.088/0.175/0.134±0.021 |
| *S. laohlie* | 4 | 0.000/0.000/0.000±0.000 | 0.031/0.167/0.128±0.029 |
| *S. latipenis* | 2 | 0.002 | 0.092/0.169/0.131±0.017 |
| *S. luchun* | 2 | 0.002 | 0.077/0.169/0.127±0.021 |
| *S. maichouensis* | 4 | 0.000/0.006/0.003±0.003 | 0.071/0.173/0.132±0.026 |
| *S. mediospinosa* | 11 | 0.002/0.035/0.019±0.009 | 0.040/0.175/0.133±0.033 |
| *S. mengwan* | 2 | 0.012 | 0.084/0.182/0.139±0.022 |
| *S. nigripennis* | 2 | 0.000 | 0.015/0.190/0.147±0.028 |
| *S. prigenti* | 2 | 0.004 | 0.081/0.188/0.142±0.021 |
| *S. setifrons* | 4 | 0.002/0.033/0.021±0.012 | 0.056/0.180/0.148±0.015 |
| *S. triodonta* | 3 | 0.000/0.004/0.003±0.002 | 0.071/0.192/0.142±0.025 |
| *S. wanglei* | 5 | 0.000/0.008/0.003±0.002 | 0.063/0.163/0.121±0.020 |
| *S. wuliangi* | 1 | NA/NA/NA | 0.094/0.177/0.147±0.021 |
| *S. xiaoyangae* | 2 | 0.006 | 0.056/0.192/0.158±0.020 |
| *S. zhulinae* | 4 | 0.000/0.002/0.001±0.001 | 0.044/0.171/0.128±0.035 |
| *S. amphigya* sp. nov. | 4 | 0.000/0.008/0.004±0.003 | 0.038/0.188/0.143±0.026 |
| *S. armillata* sp. nov. | 2 | 0.012 | 0.038/0.173/0.126±0.030 |
| *S. ashima* sp. nov. | 1 | NA/NA/NA | 0.073/0.182/0.138±0.024 |
| *S. bawo* sp. nov. | 2 | 0.012 | 0.086/0.155/0.126±0.016 |
| *S. crypta* sp. nov. | 1 | NA/NA/NA | 0.079/0.154/0.119±0.016 |
| *S. gelea* sp. nov. | 3 | 0.002/0.008/0.005/0.002 | 0.056/0.188/0.142±0.028 |
| *S. hengduanmontana* sp. nov. | 2 | 0.002 | 0.063/0.165/0.126±0.024 |
| *S. jinmingi* sp. nov. | 3 | 0.000/0.002/0.001±0.001 | 0.033/0.169/0.128±0.029 |
| *S. mengbalanaxi* sp. nov. | 2 | 0.013 | 0.060/0.186/0.145±0.023 |
| *S. mouig* sp. nov. | 8 | 0.002/0.031/0.016±0.010 | 0.071/0.165/0.132±0.018 |
| *S. setipes* sp. nov. | 1 | NA/NA/NA | 0.094/0.175/0.141±0.020 |
| *S. shangrila* sp. nov. | 1 | NA/NA/NA | 0.071/0.190/0.137±0.025 |
| *S. tsauri* sp. nov. | 1 | NA/NA/NA | 0.094/0.175/0.132±0.021 |
| *S. valleculata* sp. nov. | 3 | 0.004/0.008/0.006±0.002 | 0.084/0.175/0.127±0.018 |
| *S. wanhei* sp. nov. | 7 | 0.000/0.025/0.012±0.010 | 0.033/0.184/0.138±0.027 |
| *S. yangjin* sp. nov. | 1 | NA/NA/NA | 0.060/0.171/0.133±0.022 |
| *S. hypophaia* sp. nov. | 1 | NA/NA/NA | 0.092/0.167/0.130±0.017 |

N, Number of sequences; Min. intra., minimum intraspecific distance; Max. intra., maximum intraspecific distance; Mean intra., mean intraspecific distance (standard deviation, SD); Min. inter., minimum interspecific distance; Max. inter., maximum interspecific distance; Mean inter., mean interspecific distance (SD); NA, not applicable.
