# Supplementary material for: Cryptic diversity in the subgenus Oxyphortica (Diptera, Drosophilidae, Stegana)
Source: PeerJ. 2021 Oct 29;9:e12347. doi: 10.7717/peerj.12347 (PMC8559608; doi:10.7717/peerj.12347)
Supplement: Supplemental Information 8 [file peerj-09-12347-s008.docx]

Table S3. Summary of genetic distances of *ND2* gene.

| Species | N | Min.intra./Max.intra./Mean intra. ±SD | Min.inter./Max.inter./Mean inter. ±SD |
| --- | --- | --- | --- |
| *S. acutipenis* | 3 | 0.000/0.000/0.000±0.000 | 0.101/0.237/0.191±0.042 |
| *S. adentata* | 8 | 0.000/0.015/0.008±0.004 | 0.053/0.240/0.193±0.048 |
| *S. aotsukai* | 4 | 0.001/0.005/0.003±0.002 | 0.009/0.245/0.204±0.032 |
| *S. apicopubescens* | 4 | 0.003/0.017/0.011±0.006 | 0.070/0.254/0.186±0.056 |
| *S. apicosetosa* | 5 | 0.002/0.011/0.006±0.003 | 0.038/0.235/0.174±0.058 |
| *S. chuanjiangi* | 7 | 0.000/0.016/0.008±0.006 | 0.061/0.236/0.198±0.036 |
| *S. convergens* | 4 | 0.007/0.025/0.018±0.007 | 0.038/0.244/0.173±0.062 |
| *S. curvata* | 3 | 0.004/0.023/0.016/0.009 | 0.075/0.227/0.173±0.043 |
| *S. dainuo* | 2 | 0.031 | 0.024/0.245/0.190±0.057 |
| *S. dawa* | 1 | NA/NA/NA | 0.061/0.231/0.190±0.043 |
| *S. gonglui* | 3 | 0.010/0.012/0.010±0.001 | 0.058/0.244/0.180±0.059 |
| *S. hirtipenis* | 3 | 0.000/0.003/0.002±0.001 | 0.113/0.229/0.184±0.038 |
| *S. laohlie* | 4 | 0.000/0.005/0.003±0.002 | 0.024/0.241/0.191±0.054 |
| *S. latipenis* | 2 | 0.002 | 0.116/0.233/0.191±0.034 |
| *S. luchun* | 2 | 0.000 | 0.097/0.230/0.186±0.041 |
| *S. maichouensis* | 4 | 0.000/0.003/0.001±0.001 | 0.081/0.214/0.172±0.028 |
| *S. mediospinosa* | 11 | 0.003/0.028/0.016±0.007 | 0.052/0.242/0.180±0.053 |
| *S. mengwan* | 1 | NA/NA/NA | 0.081/0.226/0.187±0.031 |
| *S. nigripennis* | 2 | 0.003 | 0.009/0.247/0.203±0.040 |
| *S. prigenti* | 2 | 0.001 | 0.098/0.254/0.215±0.029 |
| *S. setifrons* | 4 | 0.001/0.023/0.015±0.010 | 0.060/0.240/0.193±0.041 |
| *S. triodonta* | 3 | 0.000/0.004/0.003±0.002 | 0.107/0.241/0.192±0.038 |
| *S. wanglei* | 5 | 0.002/0.009/0.006±0.002 | 0.092/0.225/0.178±0.040 |
| *S. wuliangi* | 1 | NA/NA/NA | 0.106/0.234/0.184±0.042 |
| *S. xiaoyangae* | 2 | 0.003 | 0.060/0.247/0.200±0.044 |
| *S. zhulinae* | 4 | 0.001/0.002/0.001±0.000 | 0.050/0.247/0.180±0.062 |
| *S. amphigya* sp. nov. | 4 | 0.003/0.009/0.006±0.002 | 0.047/0.222/0.170±0.042 |
| *S. armillata* sp. nov. | 2 | 0.026 | 0.047/0.230/0.178±0.043 |
| *S. ashima* sp. nov. | 1 | NA/NA/NA | 0.087/0.220/0.169±0.044 |
| *S. bawo* sp. nov. | 2 | 0.005 | 0.099/0.220/0.170±0.042 |
| *S. crypta* sp. nov. | 1 | NA/NA/NA | 0.120/0.215/0.176±0.031 |
| *S. gelea* sp. nov. | 3 | 0.001/0.006/0.004/0.002 | 0.054/0.236/0.180±0.051 |
| *S. hengduanmontana* sp. nov. | 2 | 0.002 | 0.092/0.224/0.173±0.042 |
| *S. jinmingi* sp. nov. | 3 | 0.001/0.004/0.003±0.001 | 0.047/0.231/0.176±0.053 |
| *S. mengbalanaxi* sp. nov. | 2 | 0.005 | 0.044/0.229/0.178±0.043 |
| *S. mouig* sp. nov. | 8 | 0.000/0.026/0.011±0.009 | 0.103/0.235/0.186±0.040 |
| *S. setipes* sp. nov. | 1 | NA/NA/NA | 0.090/0.235/0.182±0.045 |
| *S. shangrila* sp. nov. | 1 | NA/NA/NA | 0.075/0.234/0.180±0.044 |
| *S. tsauri* sp. nov. | 1 | NA/NA/NA | 0.102/0.231/0.182±0.042 |
| *S. valleculata* sp. nov. | 3 | 0.000/0.004/0.003±0.002 | 0.099/0.217/0.169±0.041 |
| *S. wanhei* sp. nov. | 7 | 0.000/0.021/0.008±0.007 | 0.047/0.228/0.173±0.049 |
| *S. yangjin* sp. nov. | 1 | NA/NA/NA | 0.044/0.219/0.168±0.043 |
| *S. hypophaia* sp. nov. | 1 | NA/NA/NA | 0.133/0.225/0.187±0.032 |

N, Number of sequences; Min. intra., minimum intraspecific distance; Max. intra., maximum intraspecific distance; Mean intra., mean intraspecific distance (standard deviation, SD); Min. inter., minimum interspecific distance; Max. inter., maximum interspecific distance; Mean inter., mean interspecific distance (SD); NA, not applicable.
